# Efficacy and pharmacodynamics of niraparib in BRCA-mutant and wild-type intracranial triple-negative breast cancer murine models

**DOI:** 10.1093/noajnl/vdz005

**Published:** 2019-06-04

**Authors:** Maria J Sambade, Amanda E D Van Swearingen, Marni B McClure, Allison M Deal, Charlene Santos, Kaiming Sun, Jing Wang, Keith Mikule, Carey K Anders

**Affiliations:** 1 Lineberger Comprehensive Cancer Center, University of North Carolina at Chapel Hill, Chapel Hill, North Carolina; 2 Tesaro, Inc., Durham, North Carolina; 3 Duke Cancer Institute, Duke University Health System, Durham, North Carolina

**Keywords:** blood–brain barrier, brain metastases, PARP inhibition, targeted therapy, triple-negative breast cancer

## Abstract

**Background:**

Despite the poor prognosis of triple-negative breast cancer (TNBC) brain metastases, there are no approved systemic therapies. We explored the DNA-damaging poly(ADP-ribose) polymerase inhibitor (PARPi) niraparib in intracranial mouse models of breast cancer susceptibility protein (BRCA)*-*mutant TNBC.

**Methods:**

Mice bearing intracranial human-derived TNBC cell lines (SUM149, MDA-MB-231Br, or MDA-MB-436) were treated with niraparib and monitored for survival; intracranial tissues were analyzed for PAR levels and niraparib concentration by mass spectrometry. RNASeq data of primary breast cancers using The Cancer Genome Atlas were analyzed for DNA damage signatures. Combined RAD51 and PARP inhibition in TNBC cell lines was assessed in vitro by colony-forming assays.

**Results:**

Daily niraparib increased median survival and decreased tumor burden in the *BRCA-*mutant MDA-MB-436 model, but not in the *BRCA-*mutant SUM149 or *BRCA-*wild-type MDA-MB-231Br models despite high concentrations in intracranial tumors. RAD51 inhibitor B02 was shown to sensitize all cell lines to PARP inhibition (PARPi). In the analysis of *BRCA-*mutant primary human TNBCs, gene expression predictors of PARPi sensitivity and DNA repair signatures demonstrate widespread heterogeneity, which may explain the differential response to PARPi. Interestingly, these signatures are significantly correlated to *RAD51* expression including PARPi sensitivity (*R*^2^ = 0.602, *R*^2^= 0.758).

**Conclusions:**

Niraparib penetrates intracranial tumor tissues in mouse models of TNBC with impressive single-agent efficacy in *BRCA*-mutant MDA-MB-436. Clinical evaluation of niraparib to treat TNBC brain metastases, an unmet clinical need desperate for improved therapies, is warranted. Further compromising DNA repair through RAD51 inhibition may further augment TNBC’s response to PARPi.

**Key Points**

1. Niraparib improved survival, reduced tumor burden, and was well tolerated in a mouse model of BRCA-mutant TNBC brain metastases.
2. Niraparib accumulated in intracranial tumor tissue at levels sufficient to inhibit PARP.
3. Inhibition of RAD51 with B02 enhanced the effects of niraparib treatment in TNBC cell lines.
4. Breast cancers exhibit heterogeneity in DNA damage and PARP inhibitor sensitivity signatures, which correlate with RAD51 expression.

**Importance of the Study**

No FDA-approved systemic treatments exist for triple-negative breast cancer (TNBC) brain metastases. This preclinical study supports evaluation of a brain-permeable poly (ADP-ribose) polymerase (PARP) inhibitor, niraparib, in future clinical studies for patients with TNBC brain metastases with deficient DNA repair capacity. In a sensitive *BRCA*-mutant intracranial TNBC model, niraparib as a single agent significantly improved survival compared with controls. Although niraparib did not prove effective in all models tested, concurrent RAD51 inhibition sensitized cell lines to PARP inhibition. We demonstrate this heterogeneity in PARP inhibitor sensitivity and *RAD51* expression also exists in human primary *BRCA-*mutant breast cancer. These results suggest a population of patients with *BRCA*-mutant TNBC brain metastases may benefit from systemic niraparib treatment, alone or as combination therapy. Given prior clinical trials demonstrating niraparib’s safety in humans, this strategy could be quickly translated to the design of TNBC brain metastases trials to address a significant unmet clinical need.

Triple-negative breast cancer (TNBC), defined as a lack of expression of the estrogen and progesterone receptors and HER2, is an aggressive subtype of breast cancer with high metastatic potential. Once metastatic, nearly half of patients with TNBC will develop central nervous system (CNS) disease.^1^ Survival following brain metastases arising from TNBC is less than 1 year.^2^ To date, the mainstay of therapy for TNBC brain metastases is cranial radiation and/or neurosurgical resection.^3^ In patients with brain metastases arising from breast cancer, systemic therapies markedly improve outcome after local therapy.^4,5^ As up to 80% of patients with TNBC brain metastases will have the progressive extracranial disease, systemic therapies capable of controlling both intracranial (IC) and extracranial disease are desperately needed.^2^

Although the incidence of a germ line mutation in *BRCA* is approximately 5% across all breast cancer subtypes, as many as 20% of TNBC harbor a *BRCA1 or BRCA2* mutation.^6^*BRCA1/2* loss of function, either through mutation or promoter methylation, compromises the double-strand DNA repair mechanism of homologous recombination, forcing more error-prone mechanisms such as nonhomologous end joining (NHEJ).^7^ Poly (ADP-ribose) polymerase (PARP) family proteins are known to function through a wide array of DNA repair mechanisms, including base excision, single-strand break DNA repair, NHEJ regulation, transcriptional regulation, chromatin modification, telomere length, and genomic stability.^8–10^ Consequently, PARP inhibition (PARPi), mainly through PARP1, also compromises cellular DNA repair capacity and genome integrity. The combination of deleterious *BRCA1/2* mutations in cancers like TNBC and therapeutic inhibition of PARP results in synthetic lethality in vitro and in preclinical models, which has borne out in clinical studies.^11^ On the basis of the results of the OLYMPIAD study, the PARP inhibitor, olaparib, was FDA approved in January 2018 as the first targeted therapy approved to treat patients with advanced, germ line *BRCA*-mutant breast cancer.^10,12^ More recently, another PARP inhibitor, talazoparib, was FDA approved in October 2018 based on results of the EMBRACA study illustrating improved response rates and progression-free survival compared with physician’s choice of therapy.^13^

Niraparib, a small molecule PARP inhibitor, was approved in 2017 as maintenance therapy for patients with recurrent epithelial ovarian, fallopian tube, or primary peritoneal cancer who are in complete or partial response to platinum-based chemotherapy based on the results of the Niraparib in OVArian cancer (NOVA) study.^14^ Efficacy of niraparib has also been shown preclinically as a single agent in both breast and ovarian cancer xenograft models,^15^ as well as clinically, in combination with the programmed death (PD-1) checkpoint inhibitor, pembrolizumab, in the setting of both TNBC and ovarian cancer.^16,17^ Specific to metastatic TNBC, the results of the TOPACIO study illustrated a 28% objective response rate and progression-free survival of 8.3 months for those whose tumors harbored a *BRCA* mutation. Interestingly, responses were also seen in patients with *BRCA*-wild-type advanced TNBC with and without other deficiencies in homologous recombination (HRD) DNA repair.

Advanced metastases, including brain metastases, compared with primary breast cancers, are genomically less stable with increased HRD that may sensitize some advanced metastases to PARPi.^18–22^ Notably, breast cancer brain metastases have higher HRD signatures compared with matched primary breast tumors.^18,20^ Several PARPis, including niraparib, cross the blood–brain barrier, making PARPi a promising therapeutic for patients with advanced TNBC and brain metastases.^23–25^ Thus, we chose to evaluate niraparib in IC murine models of TNBC, including efficacy, pharmacodynamics, and pharmacokinetics to support its future clinical investigation alone or in combination with DNA damaging or repair-inhibiting agents for patients with advanced TNBC and brain metastases.

## Materials and Methods

### Cell Lines and Cell Culture

The human-derived TNBC cell lines SUM149 (Asterand; basal-like *BRCA1*-mutant, PTEN−), MDA-MB-231Br (a brain trophic subclone of the MDA-MB-231 parental line created by Dr. Toshiyuki Yoneda; claudin-low *BRCA1*-wild-type, PTEN-proficient, *KRAS*-mutant, *BRAF*-mutant), and MDA-MB-436 (ATCC; claudin-low *BRCA1*-mutant, *PTEN*−mutant) were transfected with luciferase vector under control of a cytomegalovirus promoter as described previously,^23,26^ were confirmed mycoplasma-free (September 2015), and were verified by gene expression analysis.^23,26^ Cell lines were cultured in Invitrogen media with antibiotic–antimycotic additive and maintained at 37°C with 5% CO_2_: SUM149 in HuMEC + supplements + 5% fetal bovine serum (FBS), MDA-MB-231Br and MDA-MB-436 in high glucose Dulbecco’s modified Eagle’s medium + 10% FBS.

### Drug Formulations

Niraparib was provided by Tesaro for the use in these experiments. For in vitro studies, niraparib and RAD51 inhibitor B02 (Selleck #S8434) were resuspended in tissue culture-grade dimethyl sulfoxide (DMSO). For in vivo studies, weekly formulations of niraparib were made with 0.5% methylcellulose (Sigma M0262-100G, viscosity 400 cP) as follows. Water was heated in a 125-ml flask with stir bar to reach near boil (98°C), then placed on a new room temperature stir plate. Methylcellulose was added to 0.5% and stirred at room temperature for 45 min. Niraparib was added to a final concentration of 10 mg/ml to an amber bottle. One milliliter of the 0.5% suspended methylcellulose was added to the niraparib and mixed with a disposable spatula. The rest of the methylcellulose was then added, and the niraparib-methylcellulose solution was mixed overnight on a stir plate at room temperature.

### IC Mouse Orthotopic Model Efficacy and Pharmacodynamic Studies

All animal studies protocols were approved by the University of North Carolina (UNC), Chapel Hill Institutional Animal Care and Use Committee, executed by the UNC Animal Studies Core, and performed as described previously.^23,27^ For IC injections, cells were harvested in log-phase growth and suspended in Hank's Balanced Salt Solution + 0.5% FBS. Eight- to 10-week Foxn1 *nu/nu* female mice (UNC Animal Studies Core) were implanted with 200 000 cells/5 µl of suspension through stereotactic IC injection in the right striatum. Animal weights were collected from injection to death 3 times weekly. To determine tumor volumes, mice were anesthetized, injected intraperitoneally with d-luciferin dissolved in phosphate-buffered saline (150 mg/kg; Caliper Life Sciences, Hopkinton, MA), and then imaged by IVIS Lumina camera (Caliper Life Sciences). Images were analyzed with Living Image 4.0 software (Caliper Life Sciences). All values were corrected for background and recorded as photons/s.

For efficacy studies, treatment started at day 7 for MDA-MB-231Br, day 14 for SUM149, and day 14 for MDA-MB-436. In each case, mice were grouped by treatment, ensuring all groups had the same average tumor volume by bioluminescence. For efficacy studies, mice were given a daily oral gavage of 50 mg/kg niraparib or vehicle (0.5% methylcellulose). To determine median survival, animals were sacrificed when weights decreased to 20% of an individual mouse’s maximum weight. For pharmacodynamic and pharmacokinetic studies, mice were grouped and treated as in efficacy studies for 2 weeks. At 4 h from last niraparib dose, IC tumor, peri-tumoral tissue (2 mm around the perimeter of a tumor), and contralateral brain were dissected, snap frozen, and stored frozen for each mouse and stored at –80°C.

### Pharmacokinetic Determination of Niraparib in IC Tissues

Nine milliliters of 80:20 (v:v) water:acetonitrile was added for every gram of tissue from tumor-bearing mice and homogenized using a hand-held homogenizer. The sample was diluted by another 5-fold with blank mouse plasma and kept on ice. Niraparib reference and quality control standards (1 mg/ml stock in DMSO) were diluted to appropriate concentrations in blank mouse plasma. Samples, reference, and quality control standards were precipitated in a 96-well format using acetonitrile. Supernatant was removed for the analysis of niraparib content, evaporated in nitrogen at 40°C and reconstituted in 90:10 (v:v) water:acetonitrile. Samples, niraparib reference, and quality control standards were analyzed by liquid chromatography with positive electron spray ion mass spectrometry, monitored in multiple-reaction scan mode. Niraparib was quantified using the MS/MS transition masses Q1/Q3 at 321.09/304.10 *m*/*z*. The software used to acquire and process the data was Analyst^®^, version 1.6 (Applied Biosystems Sciex).

### Pharmacodynamic Analysis of Niraparib in IC Tissues

To confirm that niraparib inhibited PARP in IC tumors, tumor-bearing mice were treated as described earlier with niraparib or vehicle for 2 weeks. 25–50 mg of tumor tissue were homogenized per protocol, and lysates were used to quantify PAR (pg/ml) using a chemiluminescent enzyme-linked immunosorbent assay (ELISA) in comparison to provided standards (Trevigen, HT PARP in vivo Pharmacodynamic Assay II, #4520-096-K). PAR values were normalized across models to 100 µg of lysate.

### In Vitro Drug Studies

The RAD51 inhibitor B02 IC50s in these studies were defined as the drug concentration that decreased proliferation by 50% as measured by the conversion of MTT to formazan after 72 h. Cells were plated to early log phase, and drug added 24 h later to final DMSO concentration of 0.05%. After 72 h, MTT (3-(4,5-dimethylthazolk-2-yl)-2,5-diphenyl tetrazolium bromide, Sigma#5655) was added to cell media to 1 mg/ml and incubated for 2 h at 37°C. Formazan was extracted with 0.1% NP40 in isopropanol, pH 2, and measured at 570 nm using a Synergy spectrophotometer.

For colony-forming assays (CFAs), cells were plated to 900 cells for MDA-MB-436 and SUM149 and 600 cells for MDA-MB-231Br per 60 mm plate to reach a maximum density of 300 colony forming units in vehicle controls. Drugs were added 24 h later to a final DMSO concentration of 0.1%. After 72 h, media was changed to drug-free media, and cells were left to grow for 12–21 days. Colony-forming units were counted and normalized to plating efficiency of vehicle control.

### Data Analysis and Statistical Methods

All data analysis and statistical methods for the in vivo and in vitro analyses were performed as described previously.^23,27^ A 2-tailed *P* < .05 was considered significant.

#### Pharmacokinetic and pharmacodynamic analysis.

A 2-way ANOVA with Tukey’s test and *P*-value correction for multiple comparisons was used to compare tissue levels of niraparib (pharmacokinetics) or PAR (pharmacodynamics). A *t*-test was used to compare PAR levels in MDA-MB-436 cells treated in vitro (GraphPad Prism, v7.04)

#### Efficacy.

The Kaplan–Meier method and log-rank test were used to estimate and compare median survival between treatment groups. Median survivals, along with their 95% confidence intervals, and log-rank test *P*-values were reported (SAS, v9.3).

#### Tumor volume and weight change analysis.

Both fold change in tumor volumes and weight over time were determined relative to treatment start date. Linear mixed models, with a random intercept and slope, were used to evaluate changes over time, overall and between groups (SAS, v9.3)

#### Colony-forming assays.

Kruskal–Wallis nonparametric 1-way ANOVA was used for the analysis of in vitro CFAs testing PARP and RAD51 inhibition (GraphPad Prism, v7.04). Dunn’s multiple comparisons test was used to compare differences between groups.

#### Bioinformatic analysis.

RNA upper quantile normalized counts from the 2015 TCGA breast cancer dataset^28,29^ were downloaded from Genomic Data Commons. Of the 1091 available BRCA tumors, clinical data, *BRCA* mutational status, and PAM50 subtype for 941 tumors were available and thus included in the analysis regardless of hormone receptor expression. DNA damage gene expression signatures’ gene lists were downloaded from MsigDb, v6.2^30^. For each tumor, the average expression of the genes in each gene signature list was taken as that gene signature’s score. Pearson correlation was performed with cor.test (rad51, gene.signatures[i]) from R, v.3.5.1^31^ in Rstudio, v1.1.456^32^. *P-*values from Pearson correlation were adjusted with the Benjamini–Hochberg method using p.adjust (*P* values, method = BH) to correct for multiple testing. Adjusted *P-*values are reported in Supplementary Figure 1. Clustering was performed with heatmap.2 from ggplot2^33^.

## Ethical Standards and Approval

This article complies with all current laws of the country in which they were performed. All procedures performed in studies involving human participants were in accordance with the ethical standards of the institutional and/or national research committee and with the 1964 Declaration of Helsinki and its later amendments or comparable ethical standards.

## Results

Niraparib is a dual PARP1/2 inhibitor with in vitro IC50s in the 2–4 nM range and an in vitro CC50 in the 10–100 nM range for cells with *BRCA1*/*BRCA2* deficiencies.^9,34^ Previous studies demonstrated that Niraparib had a 5–7 day CC50 of 24 nM in SUM149 and 18 nM in MDA-MB-436^9,34^. Niraparib also inhibited MDA-MB-436 in vivo tumor growth in a subcutaneous flank model in female nude mice with 100 mg/kg once daily or 50 mg/kg twice daily.^9^

### Niraparib-Mediated PARP Inhibition Reduces Tumor Burden and Improves Survival in an IC BRCA-Mutant TNBC Model

**Fig. 1:**
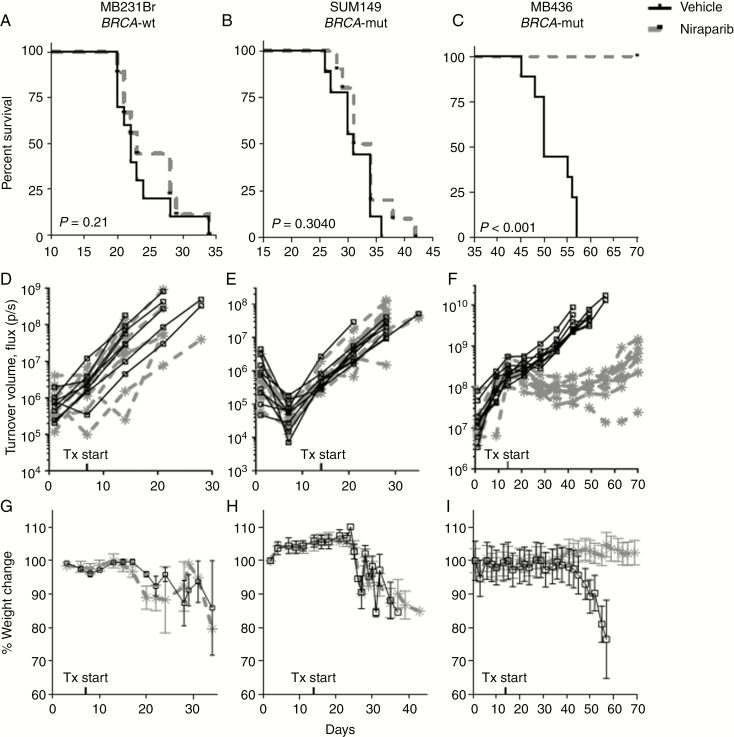
Niraparib treatment improved survival and reduced tumor burden in intracranial MDA-MB-436 model of triple-negative breast cancer (TNBC) brain metastases. Survival of mice stereotactically injected with (A) *BRCA1*-wild-type MDA-MB-231Br, (B) *BRCA1*-mutant SUM149, or (C) *BRCA1*-mutant MDA-MB-436 TNBC into the right caudate putamen and treated with vehicle or 50 mg/kg niraparib daily. Intracranial bioluminescent signal of mice stereotactically injected with (D) MDA-MB-231Br, (E) SUM149, or (F) MDA-MB-436 TNBC into the right caudate putamen and treated with vehicle or 50 mg/kg niraparib daily. Weight changes of mice with intracranial (G) MDA-MB-231Br, (H) SUM149, or (I) MDA-MB-436 TNBC treated with vehicle or 50 mg/kg niraparib daily. *x*-axes indicate the time in days since intracranial implantation of cells up to day 70 (end of study); *y*-axes indicate the percent of animals surviving (A–C), luciferin bioluminescent signal in photons/s (D–F) or the percent change in body weight relative to day 1 of the study (G–I). *n* = 9–10 per treatment group.

The effects of niraparib compared with vehicle were evaluated in 3 IC TNBC murine models, MDA-MB-231Br, a *BRCA1*-wild-type, SUM149 and MDA-MB-436, both *BRCA1*-mutant.^23,27^ Both MDA-MB-231Br and SUM149 IC orthotopic models were unresponsive to niraparib. As shown in Figure 1A and B, there was no statistically significant difference in median survival times between niraparib and vehicle control (22 vs. 23 days, *P* = .21 in MDA-MB-231Br; 34 vs. 33 days, and *P* = .30 in SUM149). Similarly, tumor volumes measured by luciferase activity (MDA-MB-231Br, Figure 1D, *P* = .7; SUM149, Figure 1E, *P* = .06) and weight changes (MDA-MB-231Br, Figure 1G, *P* = .21; SUM149, Figure 1H, *P* = .14) did not differ significantly between niraparib and vehicle control groups in the MDA-MB-231Br and SUM149 IC murine models, respectively.

Conversely, the MDA-MB-436 IC orthotopic model was highly responsive to treatment with niraparib. The median survival of the niraparib-treated group increased by at least 20 days as 100% of the niraparib-treated mice were alive at day 70 when the study ended, which was a more than 40% increase over the untreated median survival of 50 days (Figure 1C; *P* < .0001). Tumor bioluminescence was suppressed for the extent of the experiment by greater than 90% with a statistical difference between values at day 35, after 3 weeks of treatment (Figure 1F; control average = 1.15 × 10^9^ photons/s; niraparib average = 9.56 × 10^7^ photons/s; *P* < .0001). Similarly, mice treated with niraparib maintained normal weight throughout the experiment, whereas control mice lost weight as tumor burden increased (Figure 1I; *P* < .0001).

### Niraparib Is Detected in IC Tumors and Inhibits PARP in Tissues From TNBC Brain Metastases Models

**Fig. 2:**
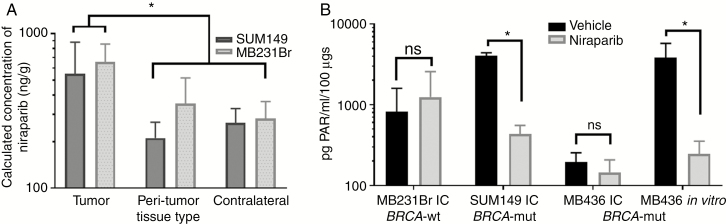
Niraparib accumulates in intracranial tissues and in some models inhibits PARP. (A) Niraparib concentration as nanogram per gram of tissue detected in tumor, peri-tumor, and contralateral normal brain tissues from mice with intracranial SUM149 and MDA-MB-231Br treated for 2 weeks with daily 50 mg/kg niraparib. *Tumor levels being significantly higher than levels in the peri-tumor (*P* = .021) and contralateral (*P* = 0.018) tissues. (B) PAR levels in intracranial (IC) tumors of mice injected with MDA-MB-231Br, SUM149, or MDA-MB-436 TNBC cells, or MDA-MB-436 in vitro, and treated with vehicle or niraparib. PAR levels as pg PAR/ml per 100-µg tissue. *PAR levels being significantly lower in niraparib treated tissue (*P* = .0027) or cells (*P* = .035) relative to vehicle treatment.

The pharmacokinetic profile of niraparib compared with vehicle control was evaluated in IC tumor, peri-tumor, and contralateral brain tissues in TNBC IC murine models. Niraparib concentrations differed significantly across IC tumor, peri-tumor (<2 mm from tumor), and contralateral brain for both SUM149 and MDA-MB-231Br (2-way ANOVA, tissue *P* < .02) but did not differ significantly between models (*P* = NS). Niraparib was detected in IC tumor tissues to a greater extent than peri-tumor (*P* < .03) and contralateral brain tissues (*P* < .02; Figure 2A and Table 1).

**Table 1.** Niraparib Concentrations in Intracranial SUM149 or MDA-MB-231Br Tumor, Peri-tumor (<2 mm away from tumor), and Contralateral Normal Brain Tissues in Mice Treated With Daily Niraparib by Oral Gavage for 2 Weeks

	Tumor		Peri-tumor		Contralateral normal	
Model	Mean	SD	Mean	SD	Mean	SD
SUM149	552.7*	328.5	211.0	56.5	265.3	63.1
MDA-MB-231Br	658.0*	198.1	353.7	164.0	282.0	81.9

Concentrations are normalized as nanogram niraparib per gram of tissue as assessed by HPLC with positive ESI MS.

*Tumor concentrations where significantly higher than peri-tumor (*P* < .03) and contralateral normal (*P* < .02) tissue, *n* = 3 per group.

To confirm pharmacodynamic inhibition of PARP in IC tumors, PAR levels were measured in IC tumor lysates using a chemiluminescent PAR ELISA (Figure 2B and Table 2). PAR levels significantly differed by treatment (*P* = .0125) and model (*P* = .0038), with a model × treatment interaction (*P* < .0015). PAR levels were higher in vehicle-treated SUM149 IC tumors than in vehicle-treated MDA-MB-231Br (*P* = .0035) or MDA-MB-436 (*P* = .0015) tumors. PARPi by niraparib was evident as reduced PAR levels in IC tumors from the SUM149 IC model (*P* = .0027), but PAR levels were not significantly altered by niraparib in the MDA-MB-231Br model (*P* = .96) or the MDA-MB-436 IC model (*P* = .99) compared with vehicle controls. To further test reduction of PAR levels in the MDA-MB-436 model, PAR analysis was performed in vitro and showed a significant reduction in PAR via niraparib treatment in this setting (unpaired *t*-test within model, vehicle vs. niraparib, *P* = .034; Figure 2B).

**Table 2.** PAR Levels in Intracranial MDA-MB-231Br, SUM149, or MDA-MB-436 Tumors From Mice Treated With Daily Vehicle or Niraparib by Oral Gavage for 2 Weeks, or MDA-MB-436 Cells Treated With Niraparib In Vitro

Model	In vivo or in vitro	Vehicle			Niraparib		
		Mean	SD	*n*	Mean	SD	*n*
MDA-MB-231Br	In vivo	821.5	777.3	5	1248.0	1327.2	6
SUM149	In vivo	4054.5	349.1	2	433.1*	122.5	3
MDA-MB-436	In vivo	195.2	59.6	3	145.7	61.9	3
	In vitro	3821.1	1955.4	3	248.2*	106.6	3

Concentrations are normalized as picogram PAR per microgram tissue as assessed by ELISA.

*Significantly lower PAR levels in tumors or cells treated with niraparib compared to controls.

### Inhibition of RAD51 Sensitizes *BRCA*-Wild-type and *BRCA*-Mutant Cell Lines to Niraparib

**Fig. 3:**
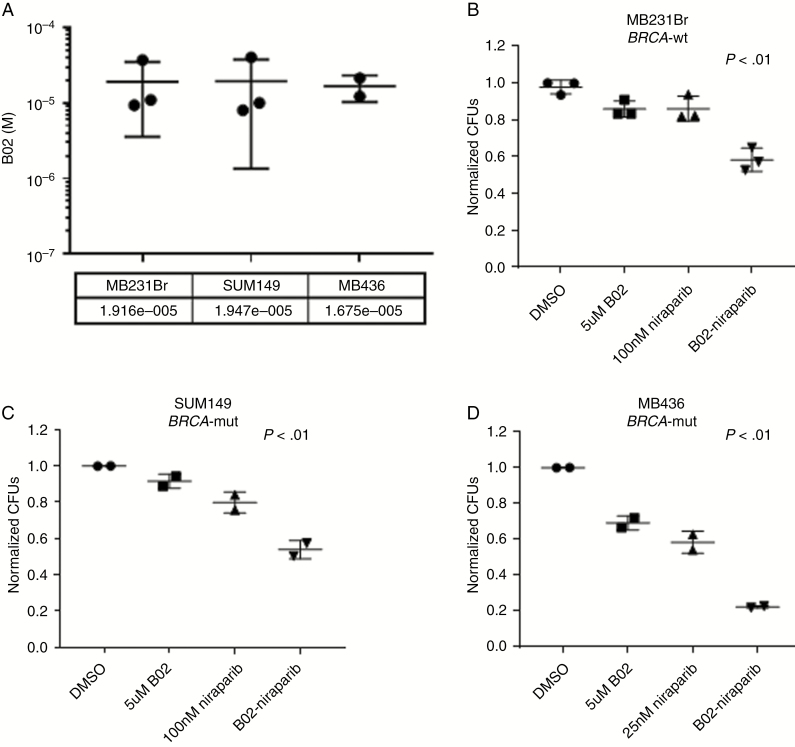
Niraparib and RAD51 inhibitor B02 decrease colony formation in TNBC cells in vitro. (A) IC50s of RAD51 inhibitor B02 in MD-MB-231Br, SUM149, and MD-MB-436 cells in vitro. Colony formation ability of MDA-MB-231Br (B), SUM149 (C), and MDA-MB-436 (D) cell in vitro after treatment with B02, Niraparib, or the combination relative to DMSO control. *n* = 2–3 experimental replicates for each line.

Given the previous literature demonstrating *RAD51* knockout sensitizing SUM149 and MDA-MB-231 to PARPi,^35,36^ we compared dual RAD51–PARPi by using the available RAD51 inhibitor B02 in combination with niraparib. All 3 cells lines had similar IC50s to the RAD51 inhibitor (Figure 3A). In both *BRCA-*wild-type (MDA-MB-231Br) and *BRCA-*mutant (SUM149, MDA-MB-436) backgrounds, RAD51 inhibition in combination with niraparib decreased colony formation compared with single-agent treatments (Figure 3B–D). The combination of RAD51 and PARPi resulted in more than 40% growth inhibition compared with vehicle control in SUM149 and MDA-MB-231Br, 20% greater than either single agent. Interestingly, the effect of the combination therapy was more than 75% in MB436 *BRCA*-mutant background.

### Heterogeneity of DNA Repair Mechanisms Across *BRCA-*Mutant TNBCs

**Fig. 4:**
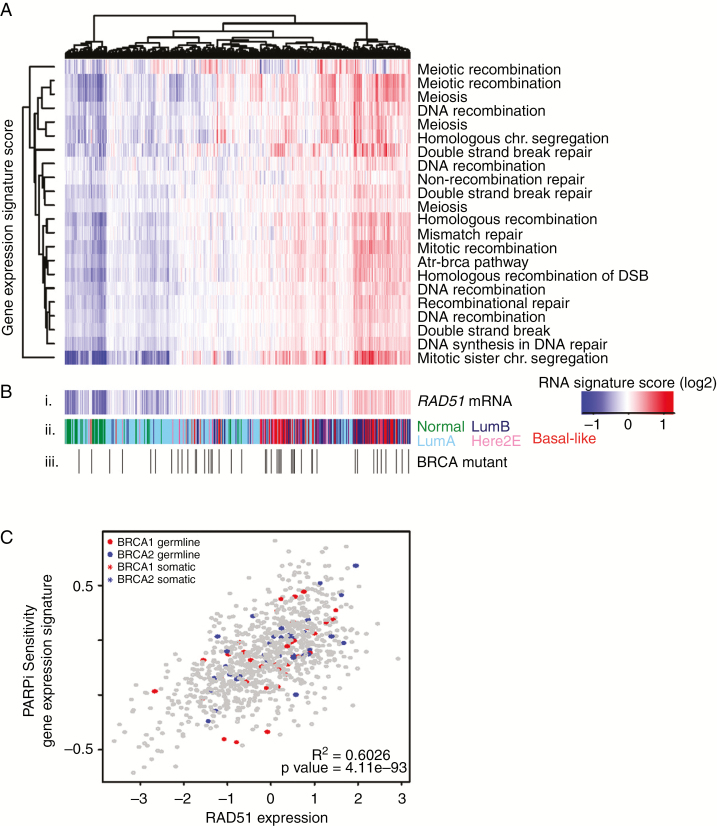
DNA damage heterogeneity across *BRCA-*mutant primary breast cancers. (A) DNA damage signatures from MSigDB demonstrate a wide range of expression across all 941 primary breast cancers from The Cancer Genome Atlas, regardless of hormone receptor status or *BRCA* mutation status, highly correlated to (B) i. *RAD51* gene expression regardless of ii. PAM50 subtype (green = normal breast; light blue = luminal A; dark blue = luminal B; pink = HER2-enriched; red = basal-like) or iii. *BRCA1/2* mutational status (white = wild-type, 861 tumors, black = germ line (26 tumors) or somatic mutant [44 tumors]). (C) *RAD51* gene expression significantly correlated with PARP inhibitor sensitivity signature regardless of *BRCA* status (*R*^2^ = 0.6026; *P* = 4.11e–93) in the same 941 tumors.

Given the differential response to PARPi in *BRCA-*mutant TNBC, consistent with previous work,^23^ we examined the heterogeneity of DNA repair through RNA-sequencing data in The Cancer Genome Atlas.^28^ To evaluate *BRCA-*mutant breast cancers, we highlight the *BRCA1* or *BRCA2* mutants on a background of the other primary tumors of all subtypes from the Breast Cancer cohort of the TCGA dataset. Across the 26 germ line-*BRCA* and 44 somatic-*BRCA* mutant tumors (Figure 4Biii), there is appreciable heterogeneity of DNA damage signatures including DNA recombination repair, double-stranded break, DNA recombination, ATR-BRCA pathway, and homologous recombination (Figure 4A).

Previous studies suggested that *RAD51* partly mediates both heterogeneity in DNA damage response of *BRCA*-mutants and differential response to PARPi. Therefore, we next examined *RAD51* expression across *BRCA-*mutants overlayed on a background of all breast cancers from the TCGA dataset (*n* = 941) and found not only significant heterogeneity across *BRCA-*mutants but that signatures associated with DNA damage repair are highly correlated with *RAD51* RNA expression (Figure 4B; Supplementary Figure 1). A publicly available gene expression signature predicting response to PARPi sensitivity based on olaparib treatment in breast cancer^37^ was used to predict PARPi sensitivity on the 941 TCGA samples, demonstrating significant positive correlation with *RAD51* RNA expression (Figure 4C, *R*^2^ = 0.6026, *P* = 4.11e–93).

## Discussion

In this study, we examined delivery and efficacy of the brain-penetrant PARP1/2 inhibitor niraparib in *BRCA-*mutant IC TNBC murine orthotopic models. Daily niraparib treatment significantly improved survival and reduced tumor burden as a single agent in *BRCA*-mutant MDA-MB-436, but not in *BRCA*-mutant SUM149 or *BRCA*-wild-type MDA-MB-231Br. Niraparib was well tolerated as mice showed no signs of overt toxicity (ie, weight loss) before succumbing to tumor burden. We further demonstrate the heterogeneity of the DNA damage response within *BRCA-*mutant TNBC in primary breast cancers, with significant correlations between these signatures and *RAD51* expression. Finally, we demonstrate in vitro that inhibition of RAD51 sensitizes SUM149, as well as 2 other TNBC cell lines, to niraparib-mediated PARPi. This work reports the first use of niraparib in a brain metastasis model and supports the utility of niraparib in *BRCA-*mutant TNBC brain metastases for which there are currently no available therapeutic options.

Consistent strides have been made demonstrating the clinical efficacy of next generation PARP inhibitors as monotherapy for patients with deleterious *BRCA1/2* mutations, demonstrating as much as 40% objective response.^6^ Interestingly, despite PAR levels being significantly reduced in SUM149 IC tumors, single agent niraparib did not yield survival benefits. This illustrates that although niraparib effectively crosses the BBB, the heterogeneous response of *BRCA-*mutant TNBC to PARPi remains an obstacle as a single-agent therapeutic approach. Examining DNA damage and HRD signatures in *BRCA-*mutant TNBC using The Cancer Genome Atlas dataset, we demonstrate significant heterogeneity within this relatively narrow subgroup of breast cancers. Using PARPi sensitivity signatures as well as DNA damage gene expression signatures, we demonstrate that these models accurately capture this diversity and replicate prior research showing that RAD51 inhibition increases sensitivity to PARPi across a variety of TNBC cell lines, offering a possible mechanism to sensitize *BRCA-*mutant TNBC to niraparib, which is certainly deserving of additional research in an expanded cell line panel.

Testing for PARP inhibitor sensitivity in the clinical setting may aid in stratifying TNBC patients that would benefit from niraparib treatment. TNBC often have significant genomic instability with widespread copy number alterations and mutations in critical genes such as *TP53, PTEN, ATM,* and *RAD51* leading to increased homologous recombination deficiency (HRD). Prior studies have demonstrated that TNBC brain metastases have even higher HRD signatures compared with matched primary tumors,^18–22^ with testing currently ongoing in TNBC brain metastases clinically (NCT02595905). Genomic mutational signatures of HRD, such as Myriad Genetics MyChoice HRD test, are also being used to correlate PARPi sensitivity to cancer’s genetic background in hopes of generating predictive markers of response.^7^

Ongoing clinical trials have been underway to combine PARPi with DNA damaging chemotherapies that induce DNA damage in complementary DNA repair pathways, including pairing niraparib with carboplatin (NCT03209401). Platinum-based chemotherapy in combination with PARPi has illustrated objective responses as high as 70% in patients with deleterious *BRCA* mutations,^6^ with previous preclinical work demonstrating effective IC penetration and response.^23^ Our analysis of primary TNBC demonstrates a significant correlation between *RAD51* expression and PARPi sensitivity, and further work shows augmentation of PARPi with concurrent RAD51 inhibition, consistent with previous research.^35,36,38,39^ Thus, among those with tumors overexpressing RAD51, a combination of RAD51 and PARPi may offer further opportunities for combinatorial therapy in advanced *BRCA-*mutant TNBC, which may otherwise be resistant to single-agent niraparib treatment.^38,39^

The results of this study should be interpreted in the context of its limitations. We compared the effects of niraparib in both *BRCA*-mutant and *BRCA*-wild-type orthotopic IC TNBC murine models and observed improved survival and tumor burden in only 1 of the 2 *BRCA*-mutant models. The lack of efficacy was not due to poor brain penetration or effective inhibition of target, as both unresponsive models showed detectable levels of niraparib and a significant reduction in PAR levels in IC tumor tissues. Thus, factors beyond pharmacokinetic and pharmacodynamics contribute to lack of efficacy and are deserving of continued research in an expanded panel of in vivo models of TNBC brain metastases providing greater generalizability to our findings across the genetic/genomic heterogeneity of TNBC. Moreover, and in comparison to the MDA-MB-231BR cell line, the SUM149 and MDA-MB-436 models were not serially passaged intracranially to select for a brain-specific phenotype; however, we believe these models are still valuable models to study the pharmacodynamics, pharmacokinetics, and efficacy as reproducible models of TNBC brain metastases.

At present, there are no approved systemic therapies for patients with TNBC brain metastases, a disease associated with limited survival, to control IC tumor burden concurrent with control of extracranial disease. As niraparib has shown safety and efficacy in advanced TNBC, both intracranially (this study) and extracranially,^15^ this body of preclinical work could be rapidly translated to the design of clinical trials that expand to use against brain metastases. In closing, brain-penetrant niraparib offers a promising “head-to-toe” systemic PARPi treatment strategy against metastatic TNBC and should strongly be considered, alone and in combination, for continued development in the clinical setting.

## Funding

This research was supported by Tesaro, Inc., as a grant to C.K.A, as well as NCI (K23157728 to C.K.A.; CA16086 to C.S.). The content is solely the responsibility of the authors.

## Supplementary Material

vdz005_suppl_Supplementary_Figure_1
