# Supplementary material for: Efficacy and pharmacodynamics of niraparib in BRCA-mutant and wild-type intracranial triple-negative breast cancer murine models
Source: Neurooncol Adv. 2019 Jun 4;1(1):vdz005. doi: 10.1093/noajnl/vdz005 (PMC7212882; doi:10.1093/noajnl/vdz005)
Supplement: vdz005_suppl_Supplementary_Figure_1 [file vdz005_suppl_supplementary_figure_1.pdf]

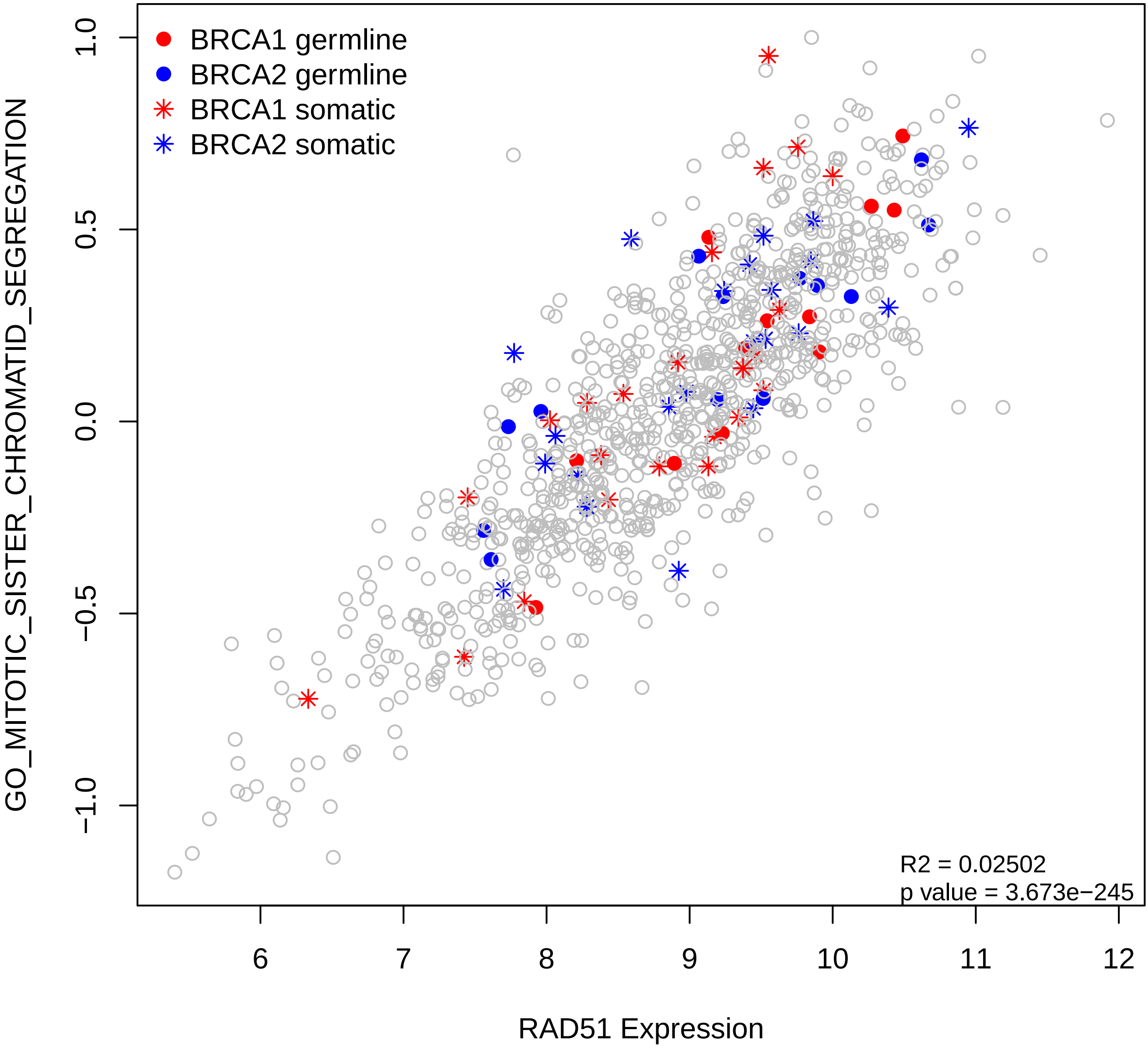
BRCA1 germline
BRCA2 germline
BRCA1 somatic
BRCA2 somatic
GO_MITOTIC_SISTER_CHROMATID_SEGREGATION
1.0
0.5
0.0
−0.5
−1.0
6
7
8
9
10
11
12
RAD51 Expression
R2 = 0.02502
p value = 3.673e−245

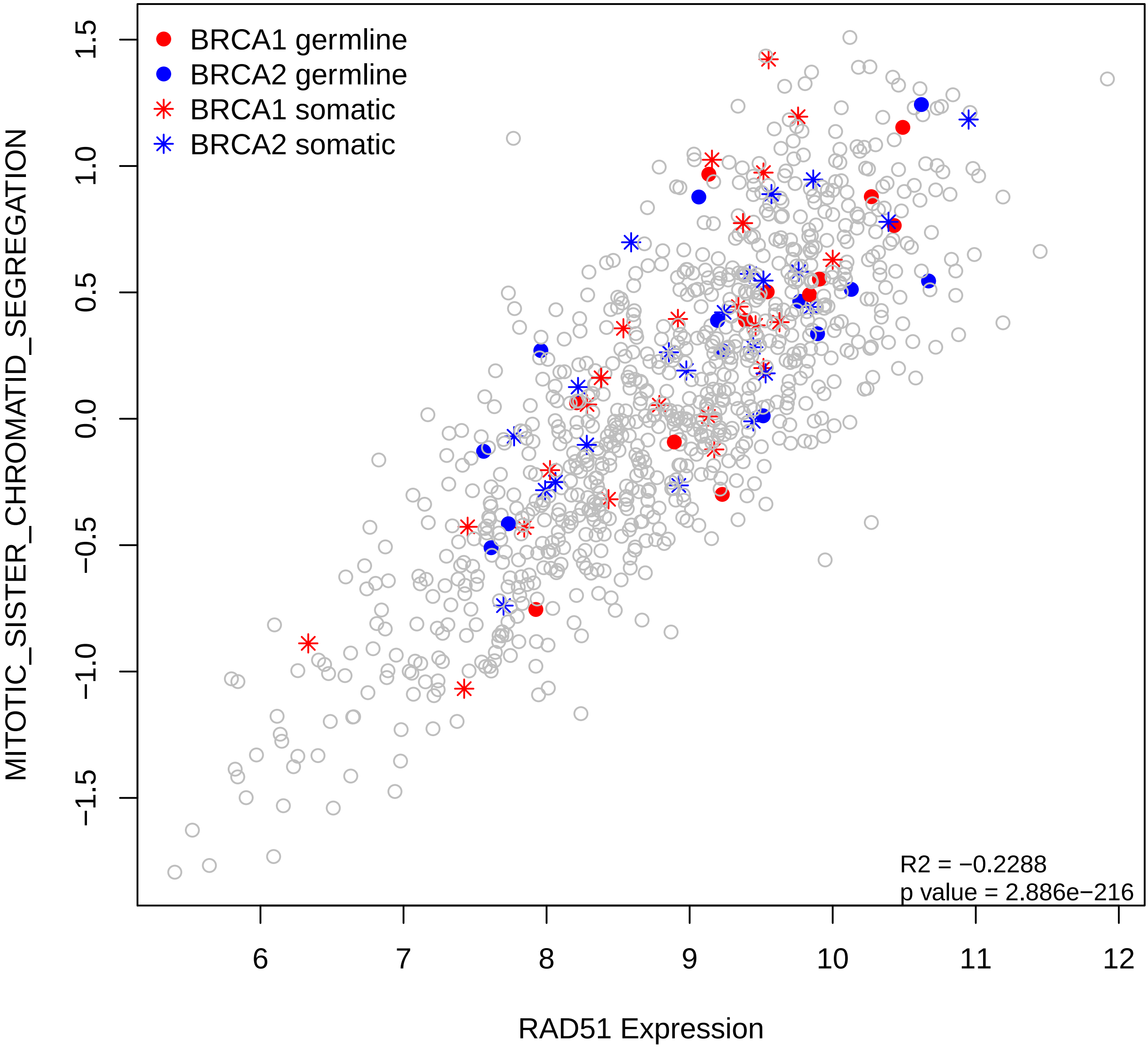

BRCA1 germline
BRCA2 germline
BRCA1 somatic
BRCA2 somatic
R2 = −0.2288
p value = 2.886e−216
MITOTIC_SISTER_CHROMATID_SEGREGATION
RAD51 Expression
1.5
1.0
0.5
0.0
−0.5
−1.0
−1.5
6
7
8
9
10
11
12

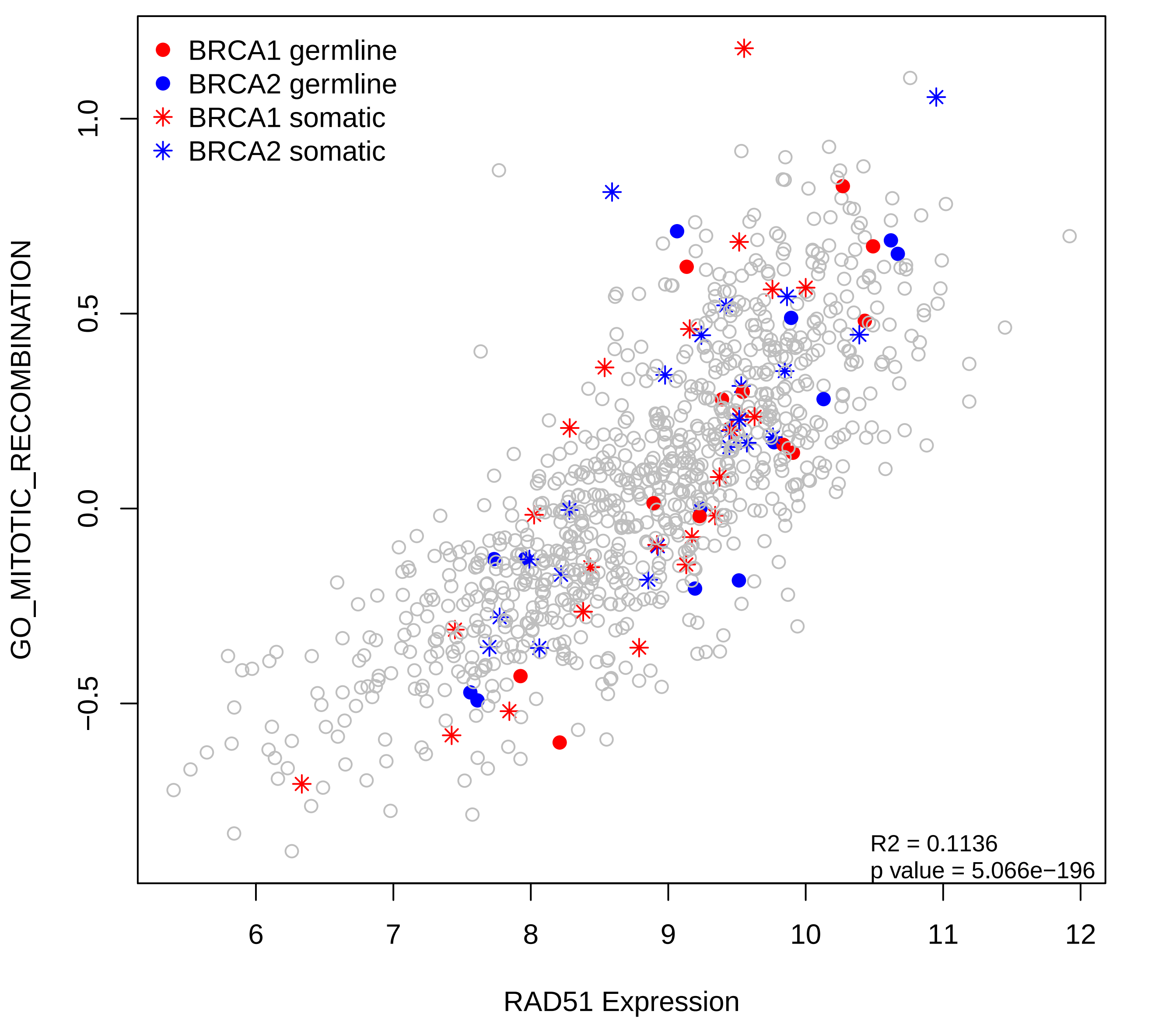
BRCA1 germline
BRCA2 germline
BRCA1 somatic
BRCA2 somatic
R2 = 0.1136
p value = 5.066e−196
GO_MITOTIC_RECOMBINATION
RAD51 Expression
−0.5
0.0
0.5
1.0
6
7
8
9
10
11
12

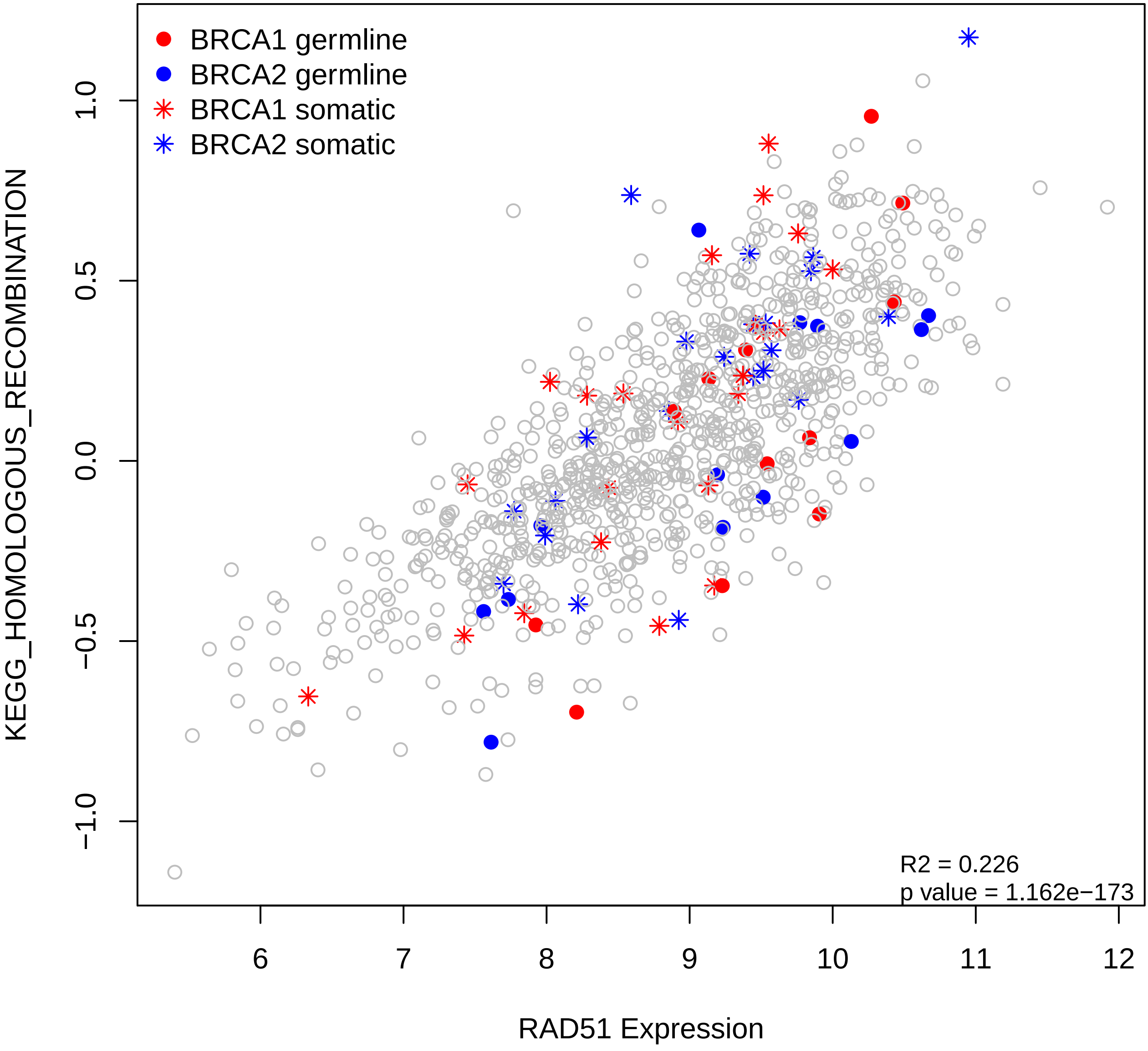

BRCA1 germline
BRCA2 germline
BRCA1 somatic
BRCA2 somatic
KEGG_HOMOLOGOUS_RECOMBINATION
RAD51 Expression
R2 = 0.226
p value = 1.162e−173
1.0
0.5
0.0
−0.5
−1.0
6
7
8
9
10
11
12

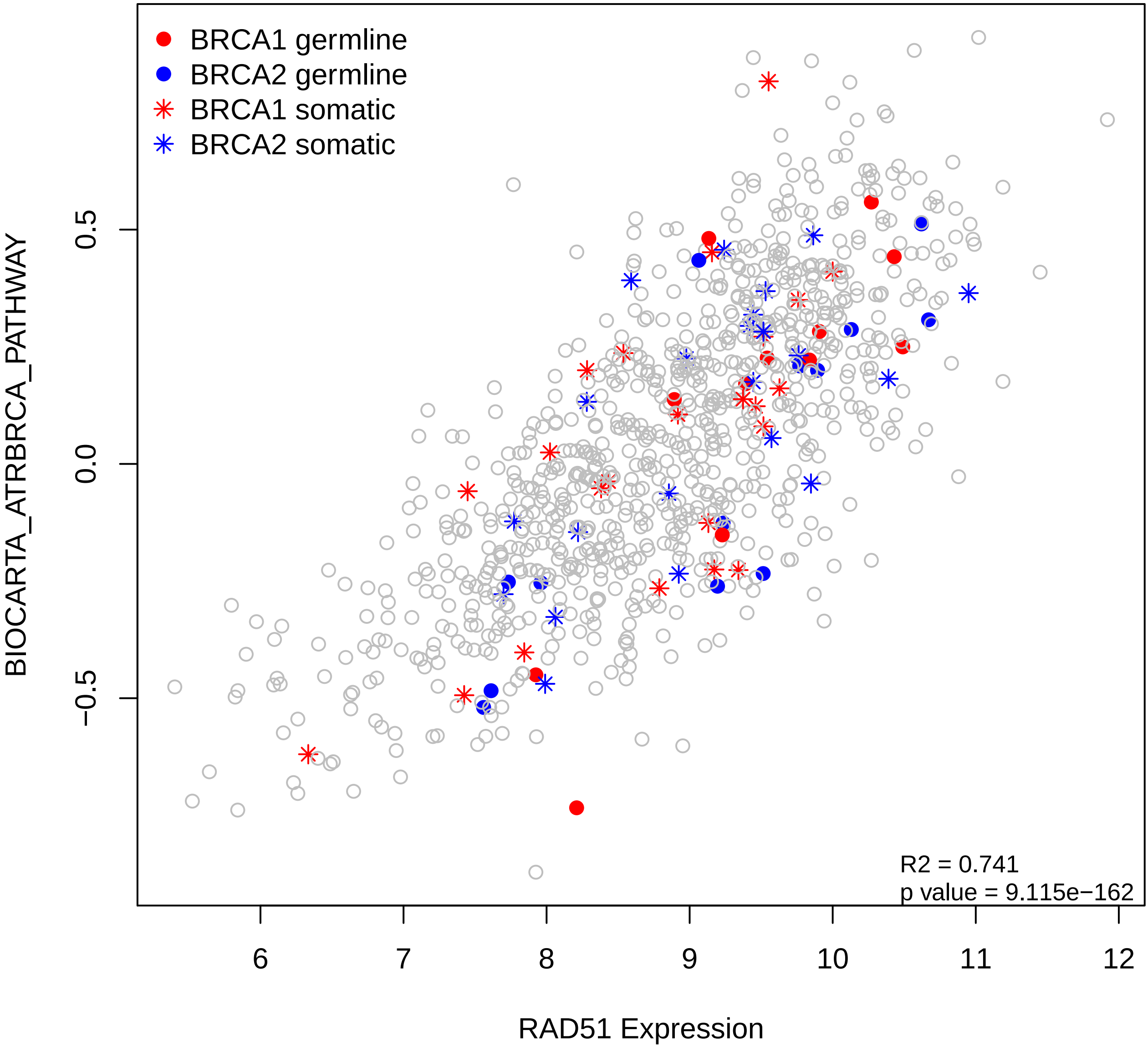

BRCA1 germline
BRCA2 germline
BRCA1 somatic
BRCA2 somatic
BIOCARTA_ATRBRCA_PATHWAY
−0.5
0.0
0.5
6
7
8
9
10
11
12
RAD51 Expression
R2 = 0.741
p value = 9.115e−162

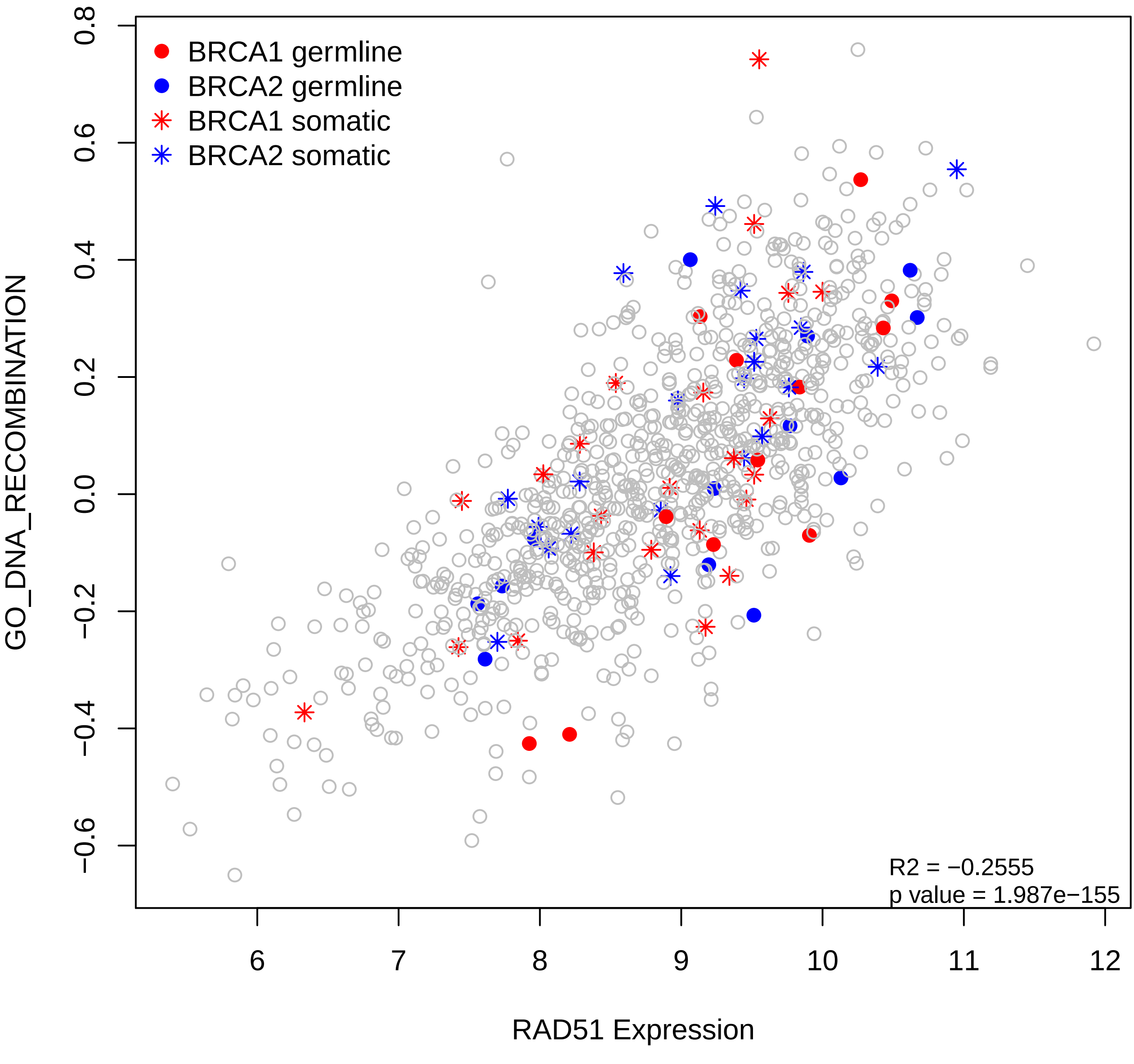

BRCA1 germline
BRCA2 germline
BRCA1 somatic
BRCA2 somatic
R2 = −0.2555
p value = 1.987e−155
GO_DNA_RECOMBINATION
RAD51 Expression
0.8
0.6
0.4
0.2
0.0
−0.2
−0.4
−0.6
6
7
8
9
10
11
12

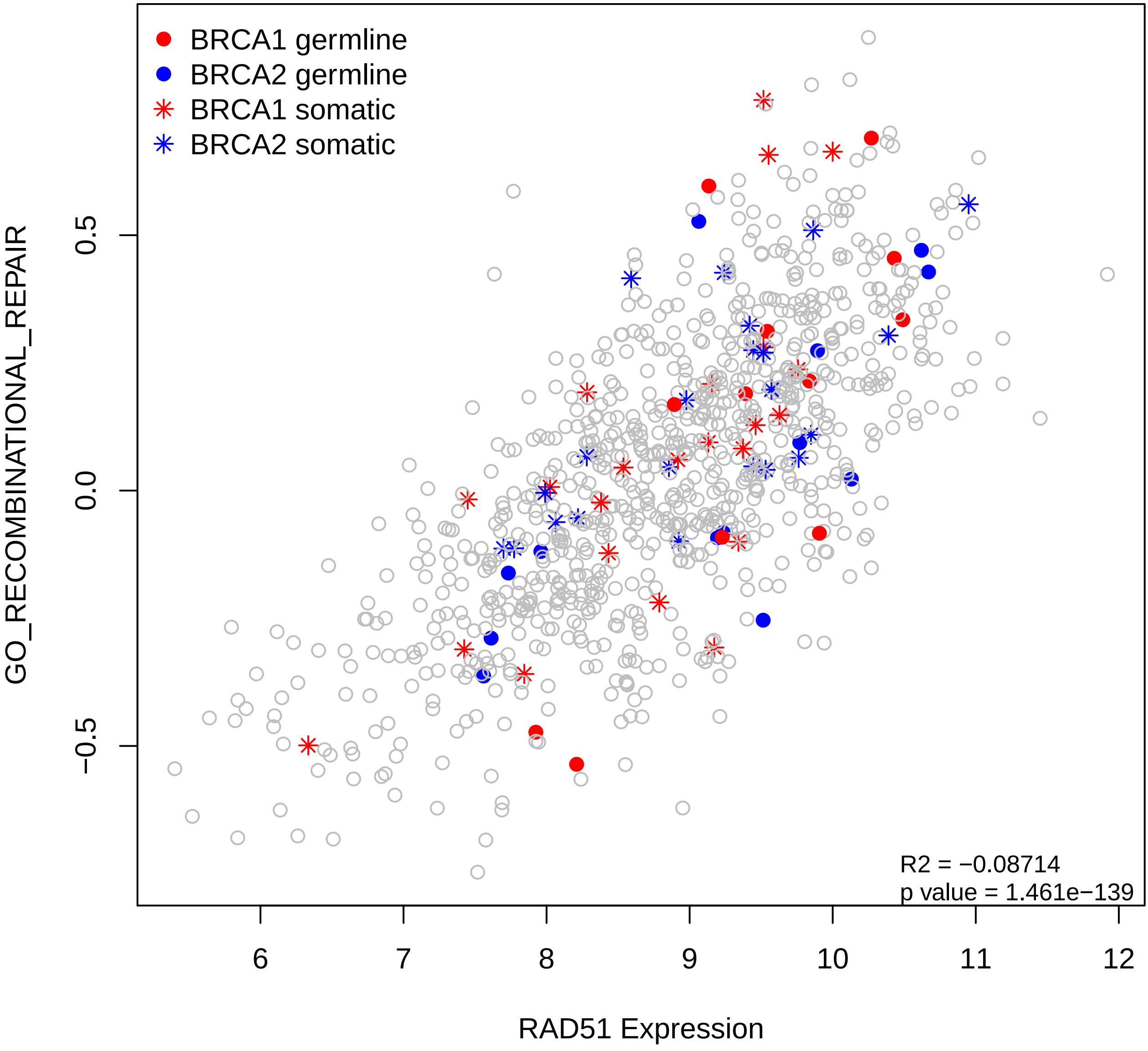
BRCA1 germline
BRCA2 germline
BRCA1 somatic
BRCA2 somatic
R2 = −0.08714
p value = 1.461e−139
GO_RECOMBINATIONAL_REPAIR
RAD51 Expression
0.5
0.0
−0.5
6
7
8
9
10
11
12

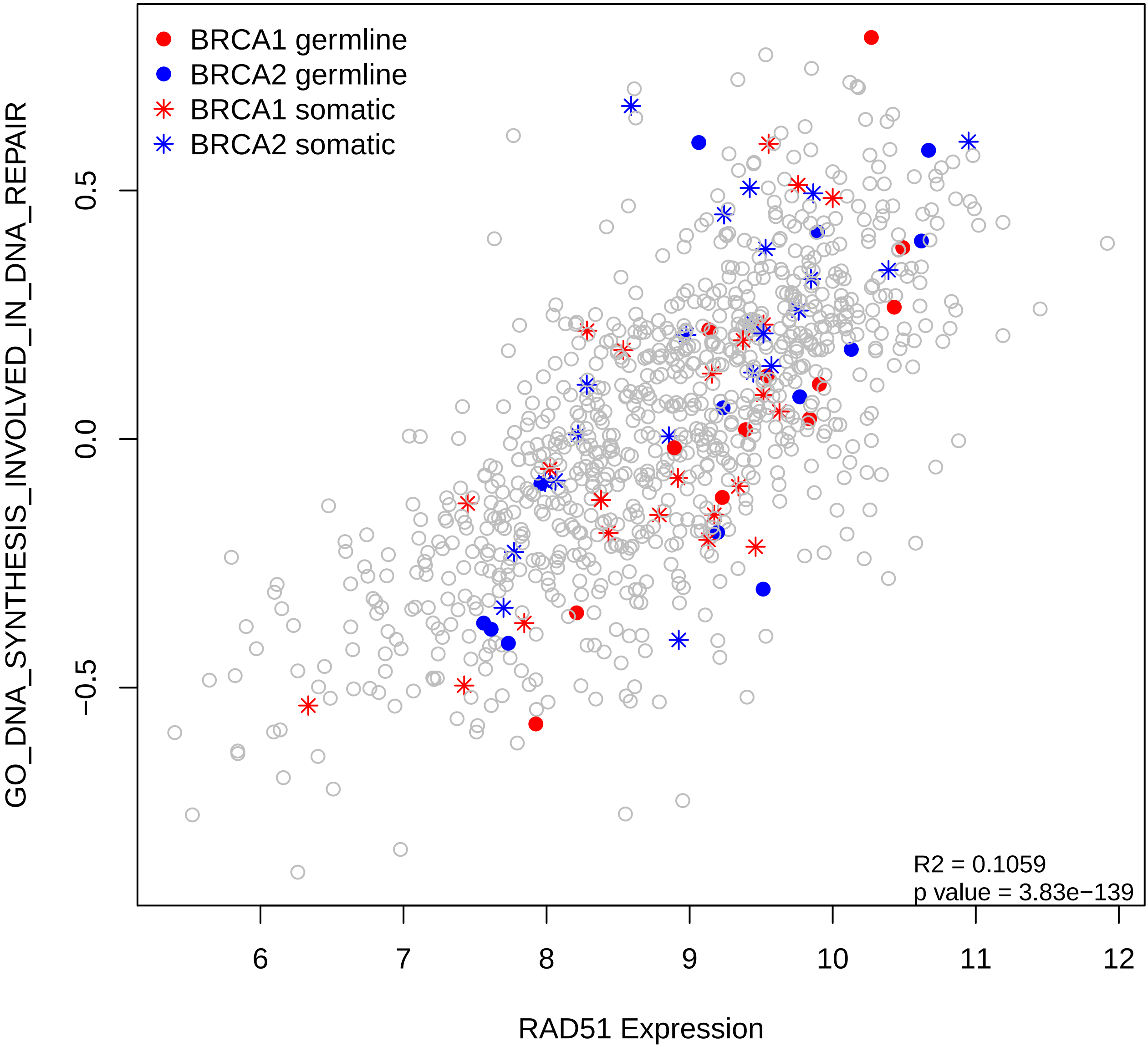

BRCA1 germline
BRCA2 germline
BRCA1 somatic
BRCA2 somatic
GO_DNA_SYNTHESIS_INVOLVED_IN_DNA_REPAIR
0.5
0.0
−0.5
R2 = 0.1059
p value = 3.83e−139
6
7
8
9
10
11
12
RAD51 Expression

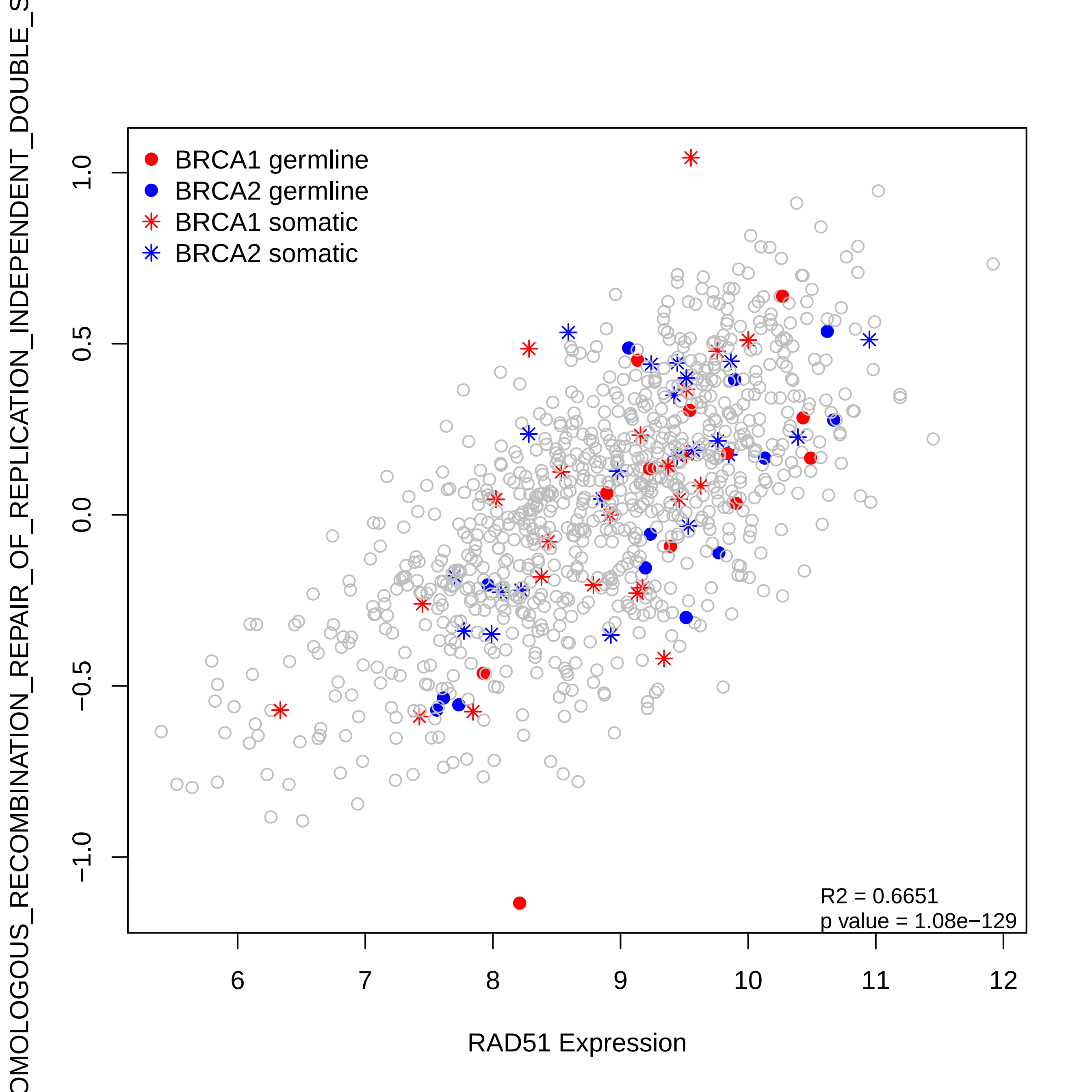
BRCA1 germline
BRCA2 germline
BRCA1 somatic
BRCA2 somatic
R2 = 0.6651
p value = 1.08e−129
RAD51 Expression
OMOLOGOUS_RECOMBINATION_REPAIR_OF_REPLICATION_INDEPENDENT_DOUBLE_S
6
7
8
9
10
11
12
−1.0
−0.5
0.0
0.5
1.0

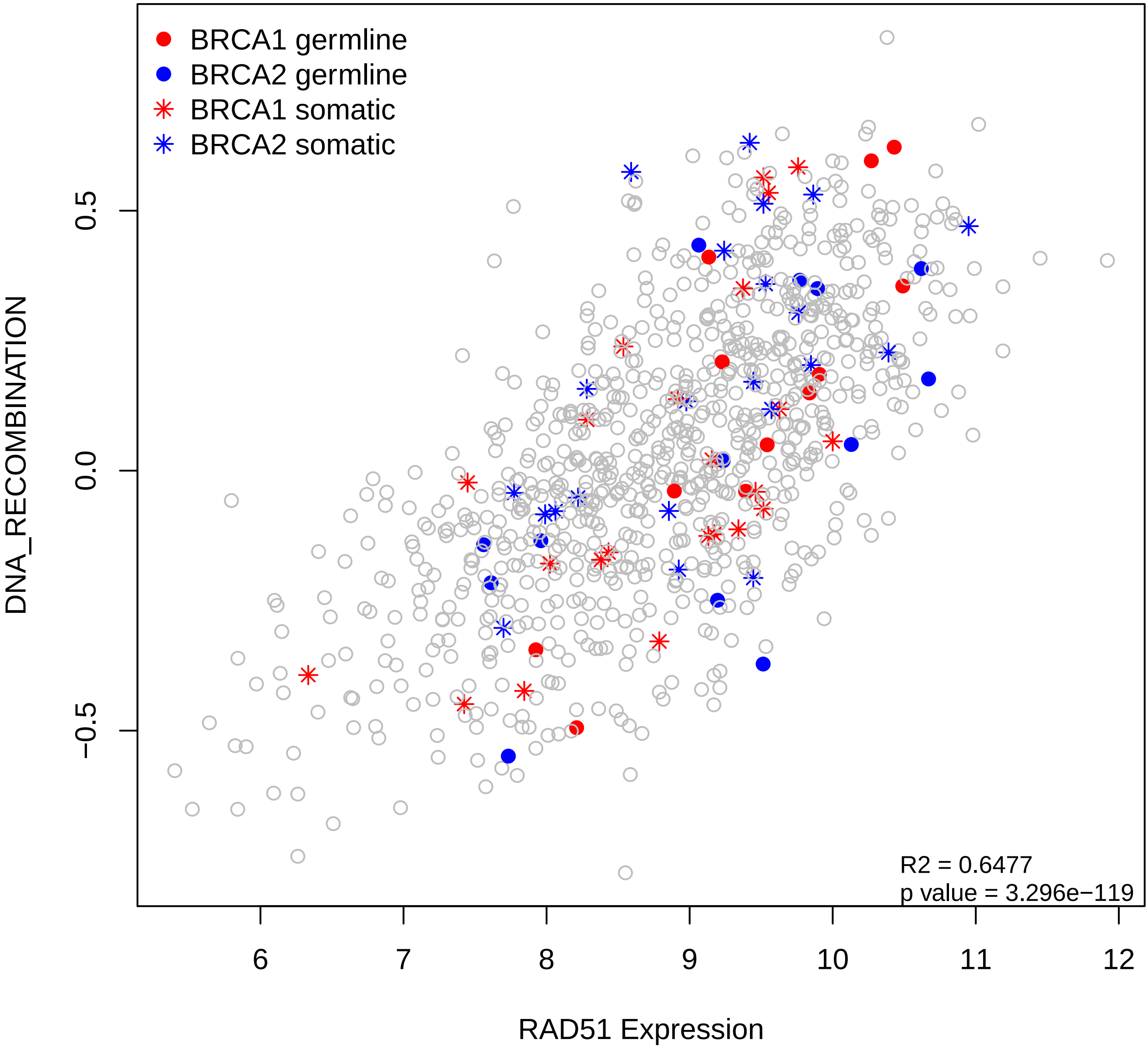
BRCA1 germline
BRCA2 germline
BRCA1 somatic
BRCA2 somatic
R2 = 0.6477
p value = 3.296e−119
0.5
0.0
−0.5
6
7
8
9
10
11
12
RAD51 Expression
DNA_RECOMBINATION

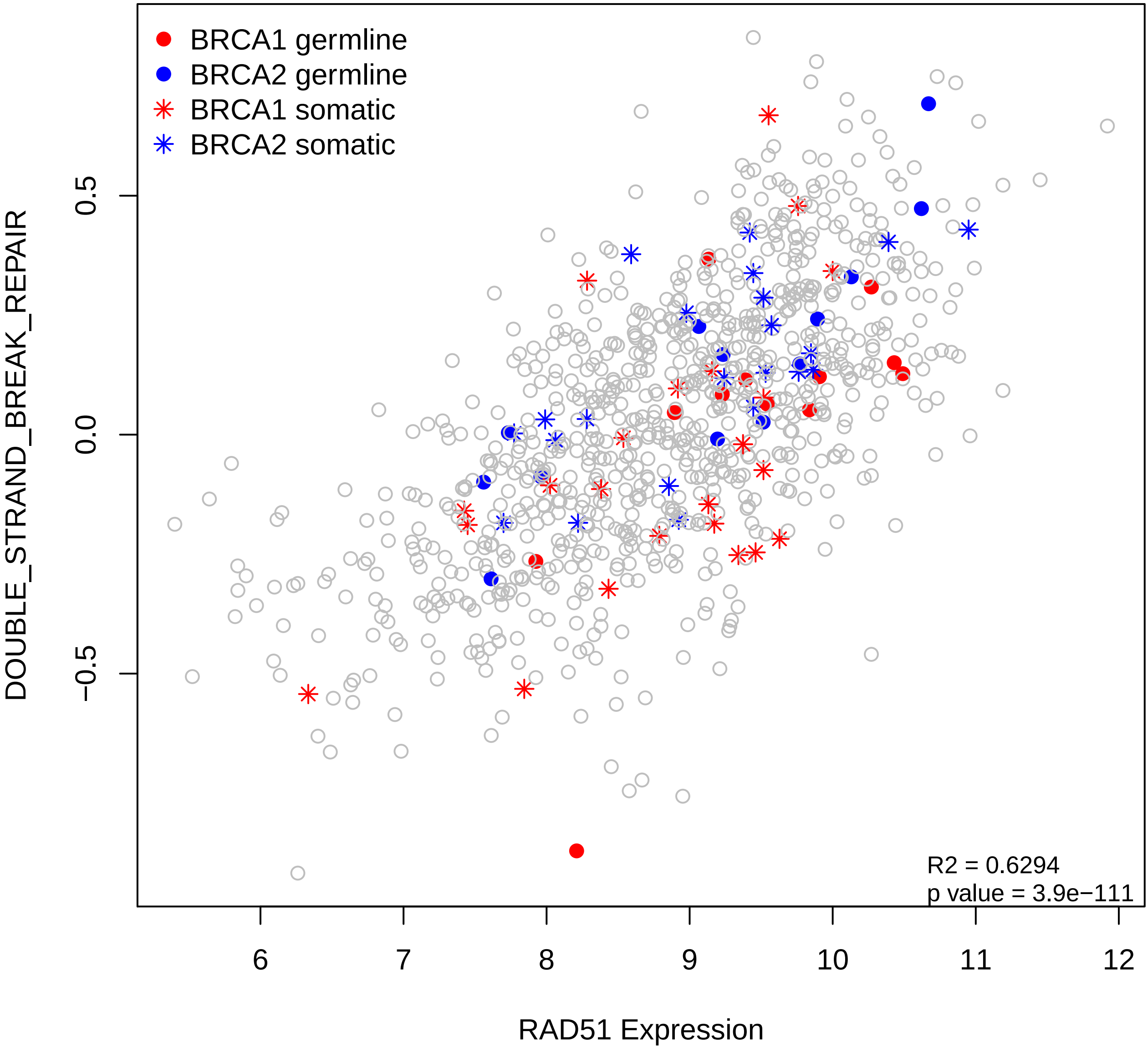

BRCA1 germline
BRCA2 germline
BRCA1 somatic
BRCA2 somatic
R2 = 0.6294
p value = 3.9e−111
DOUBLE_STRAND_BREAK_REPAIR
RAD51 Expression
0.5
0.0
−0.5
6
7
8
9
10
11
12

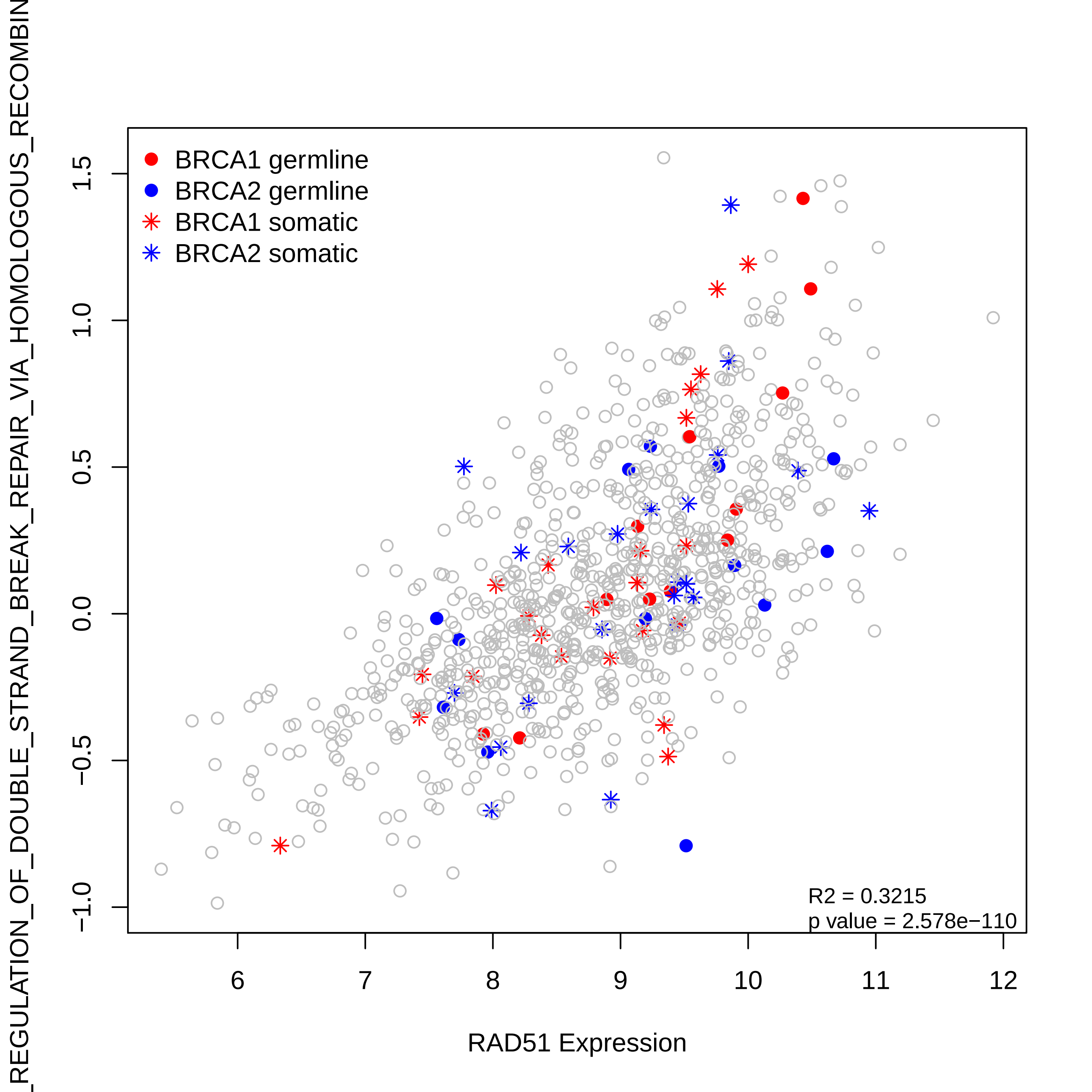
BRCA1 germline
BRCA2 germline
BRCA1 somatic
BRCA2 somatic
R2 = 0.3215
p value = 2.578e−110
RAD51 Expression
REGULATION_OF_DOUBLE_STRAND_BREAK_REPAIR_VIA_HOMOLOGOUS_RECOMBIN
6
7
8
9
10
11
12
−1.0
−0.5
0.0
0.5
1.0
1.5

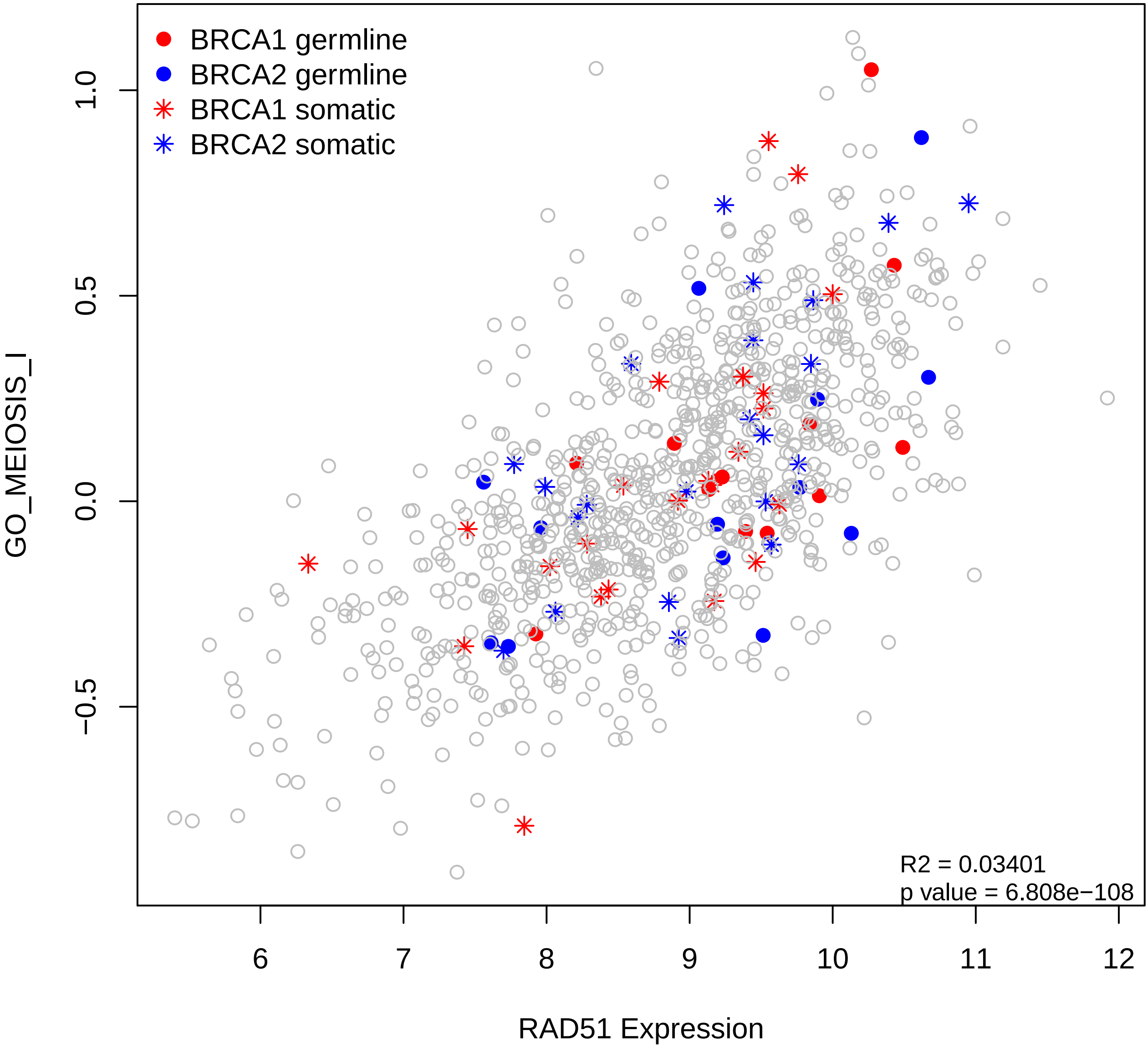

BRCA1 germline
BRCA2 germline
BRCA1 somatic
BRCA2 somatic
GO_MEIOSIS_I
RAD51 Expression
R2 = 0.03401
p value = 6.808e−108
1.0
0.5
0.0
−0.5
6
7
8
9
10
11
12

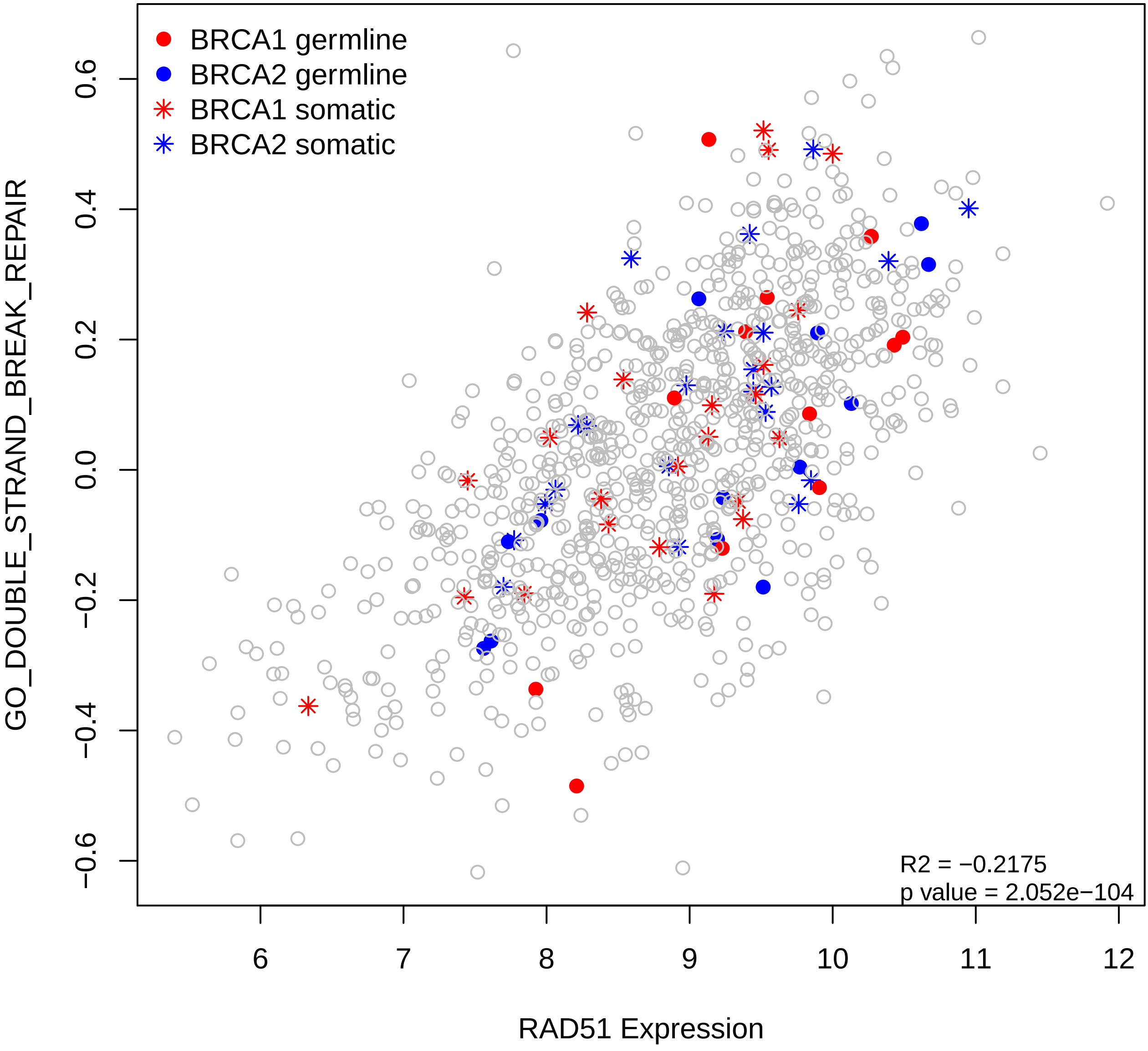
BRCA1 germline
BRCA2 germline
BRCA1 somatic
BRCA2 somatic
GO_DOUBLE_STRAND_BREAK_REPAIR
RAD51 Expression
R2 = −0.2175
p value = 2.052e−104
0.6
0.4
0.2
0.0
−0.2
−0.4
−0.6
6
7
8
9
10
11
12

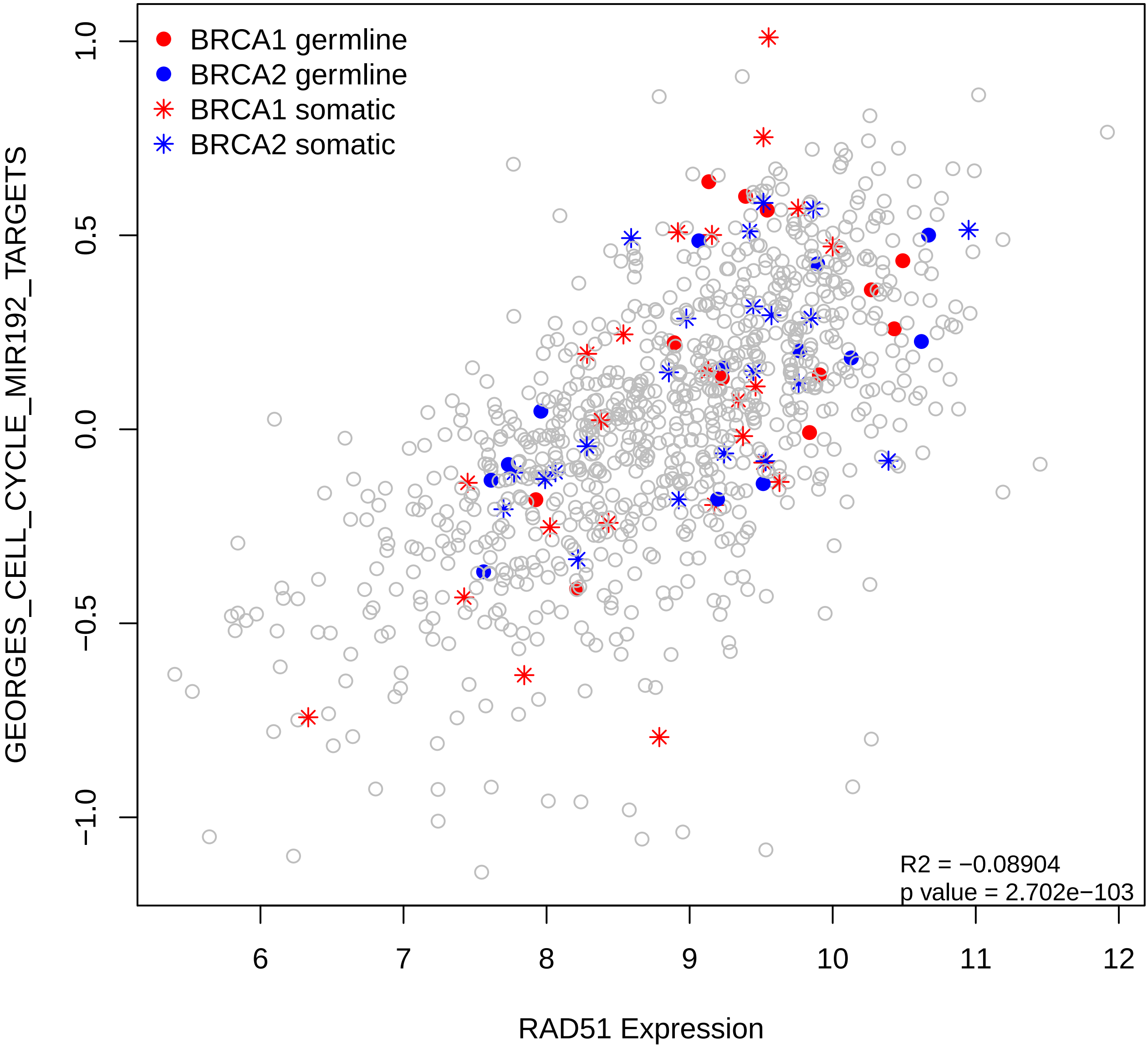
BRCA1 germline
BRCA2 germline
BRCA1 somatic
BRCA2 somatic
R2 = −0.08904
p value = 2.702e−103
GEORGES_CELL_CYCLE_MIR192_TARGETS
RAD51 Expression
1.0
0.5
0.0
−0.5
−1.0
6
7
8
9
10
11
12

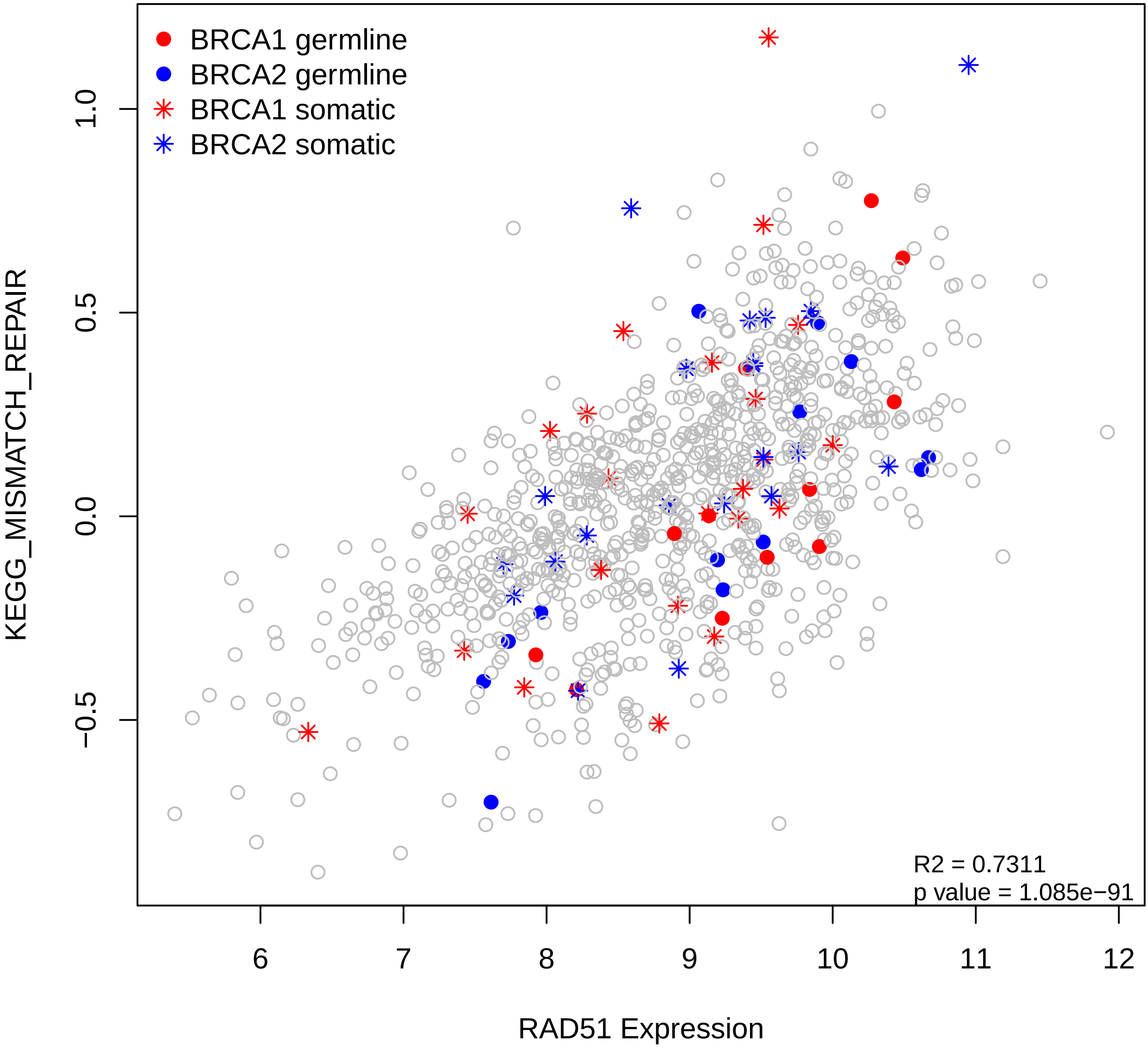

BRCA1 germline
BRCA2 germline
BRCA1 somatic
BRCA2 somatic
KEGG_MISMATCH_REPAIR
1.0
0.5
0.0
−0.5
6
7
8
9
10
11
12
RAD51 Expression
R2 = 0.7311
p value = 1.085e−91

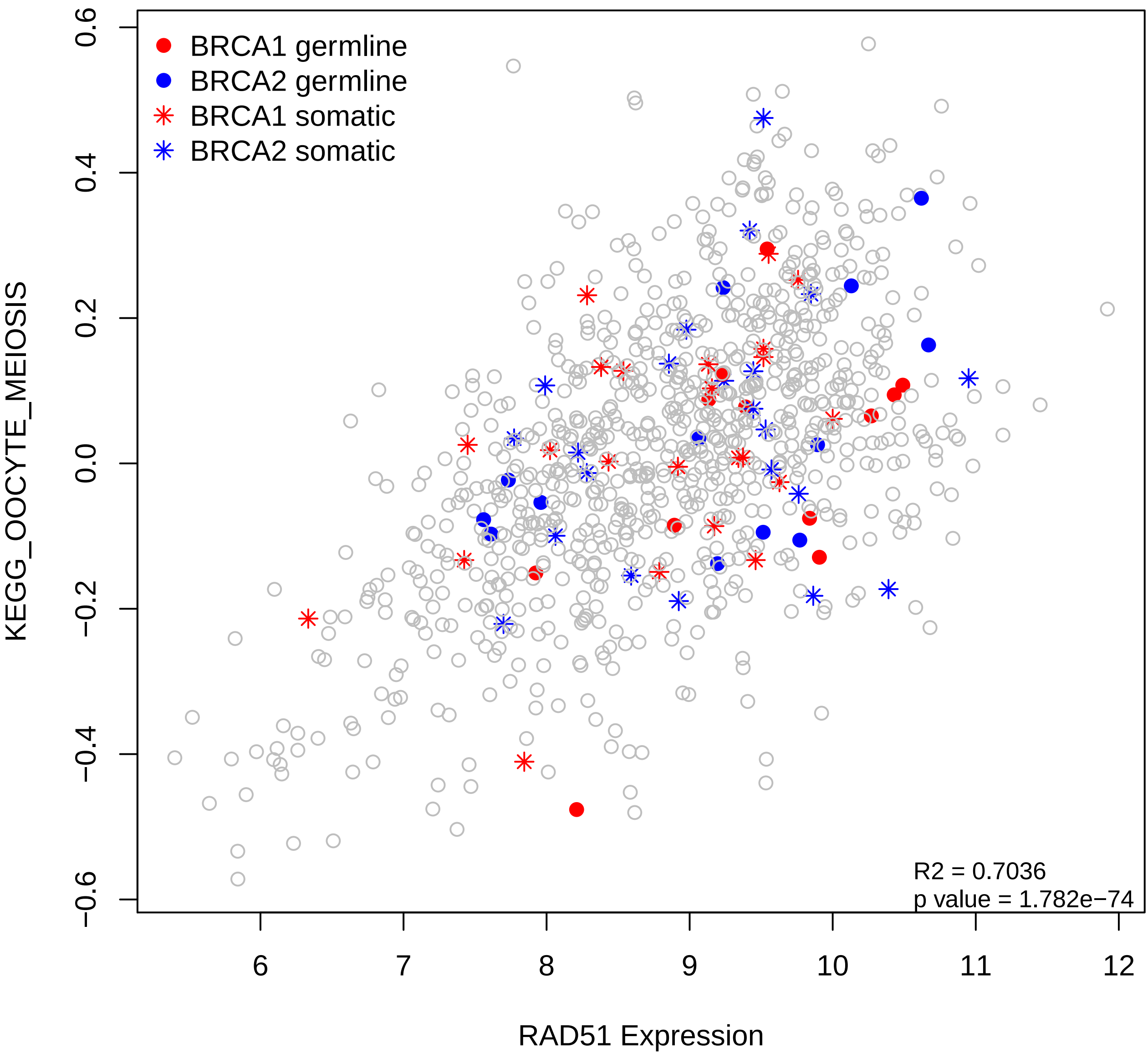
BRCA1 germline
BRCA2 germline
BRCA1 somatic
BRCA2 somatic
R2 = 0.7036
p value = 1.782e−74
KEGG_OOCYTE_MEIOSIS
RAD51 Expression
0.6
0.4
0.2
0.0
−0.2
−0.4
−0.6
6
7
8
9
10
11
12

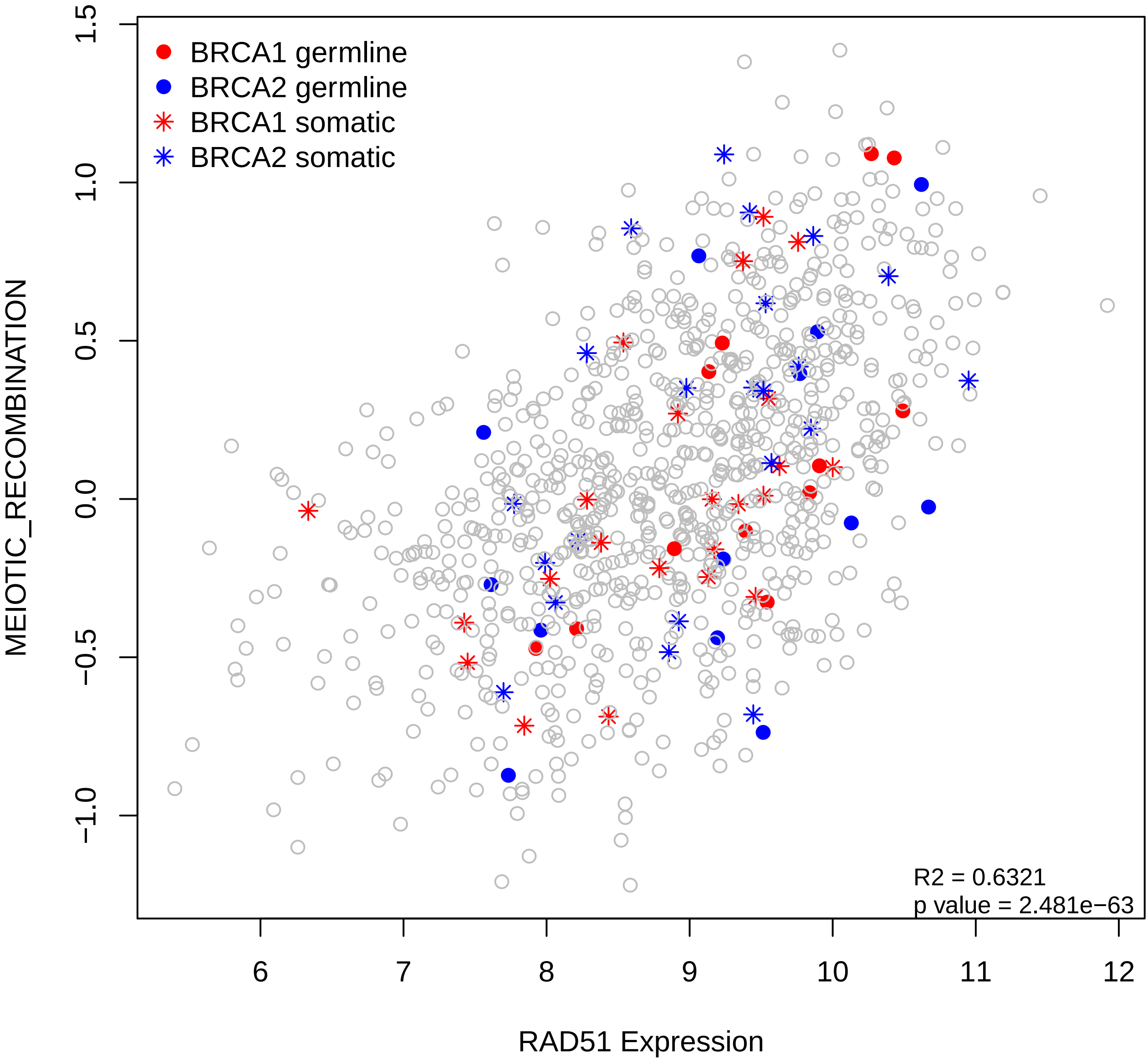
BRCA1 germline
BRCA2 germline
BRCA1 somatic
BRCA2 somatic
R2 = 0.6321
p value = 2.481e−63
MEIOTIC_RECOMBINATION
RAD51 Expression
1.5
1.0
0.5
0.0
−0.5
−1.0
6
7
8
9
10
11
12

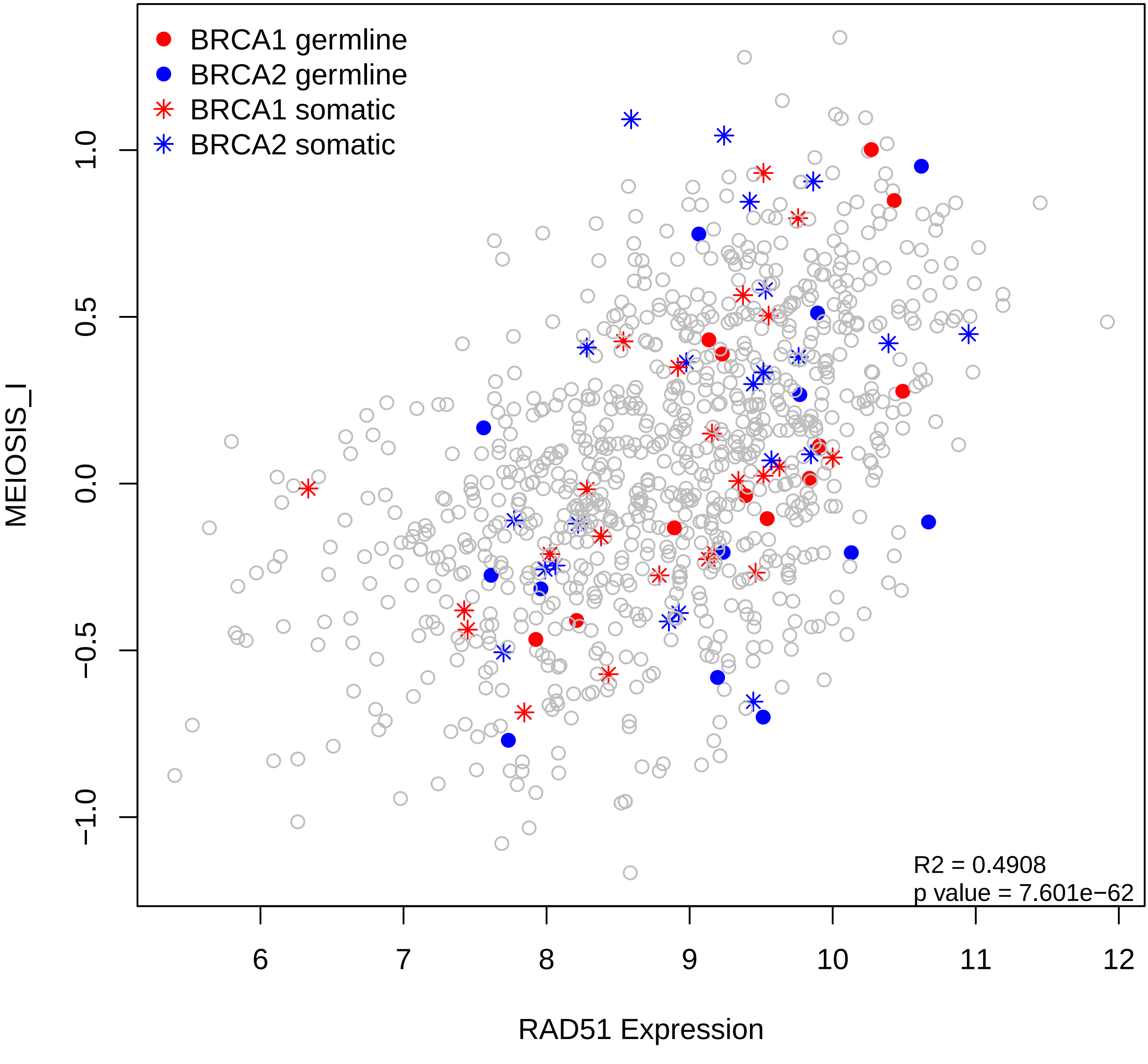

BRCA1 germline
BRCA2 germline
BRCA1 somatic
BRCA2 somatic
MEIOSIS_I
1.0
0.5
0.0
−0.5
−1.0
6
7
8
9
10
11
12
RAD51 Expression
R2 = 0.4908
p value = 7.601e−62

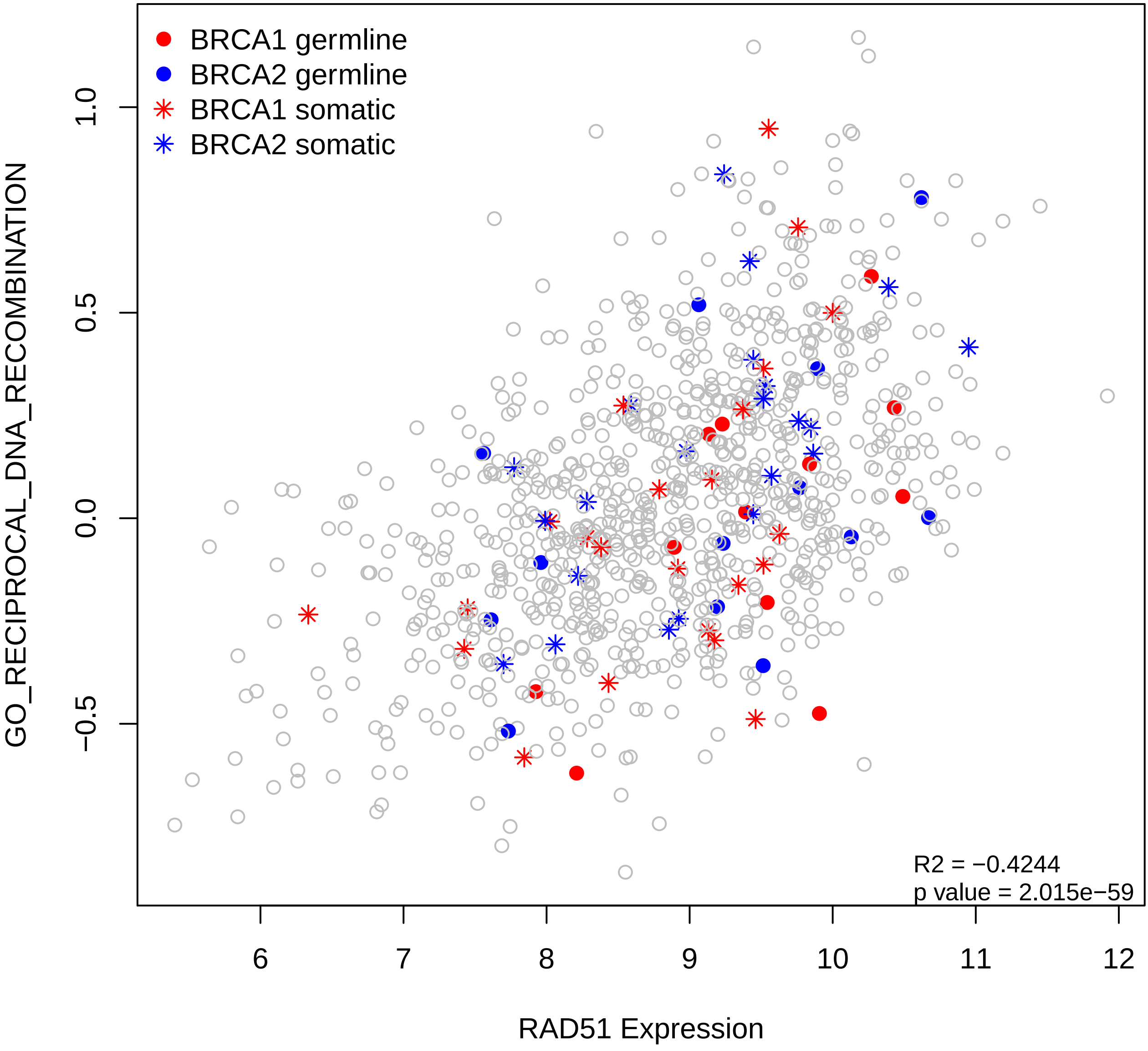

BRCA1 germline
BRCA2 germline
BRCA1 somatic
BRCA2 somatic
GO_RECIPROCAL_DNA_RECOMBINATION
1.0
0.5
0.0
−0.5
6
7
8
9
10
11
12
RAD51 Expression
R2 = −0.4244
p value = 2.015e−59

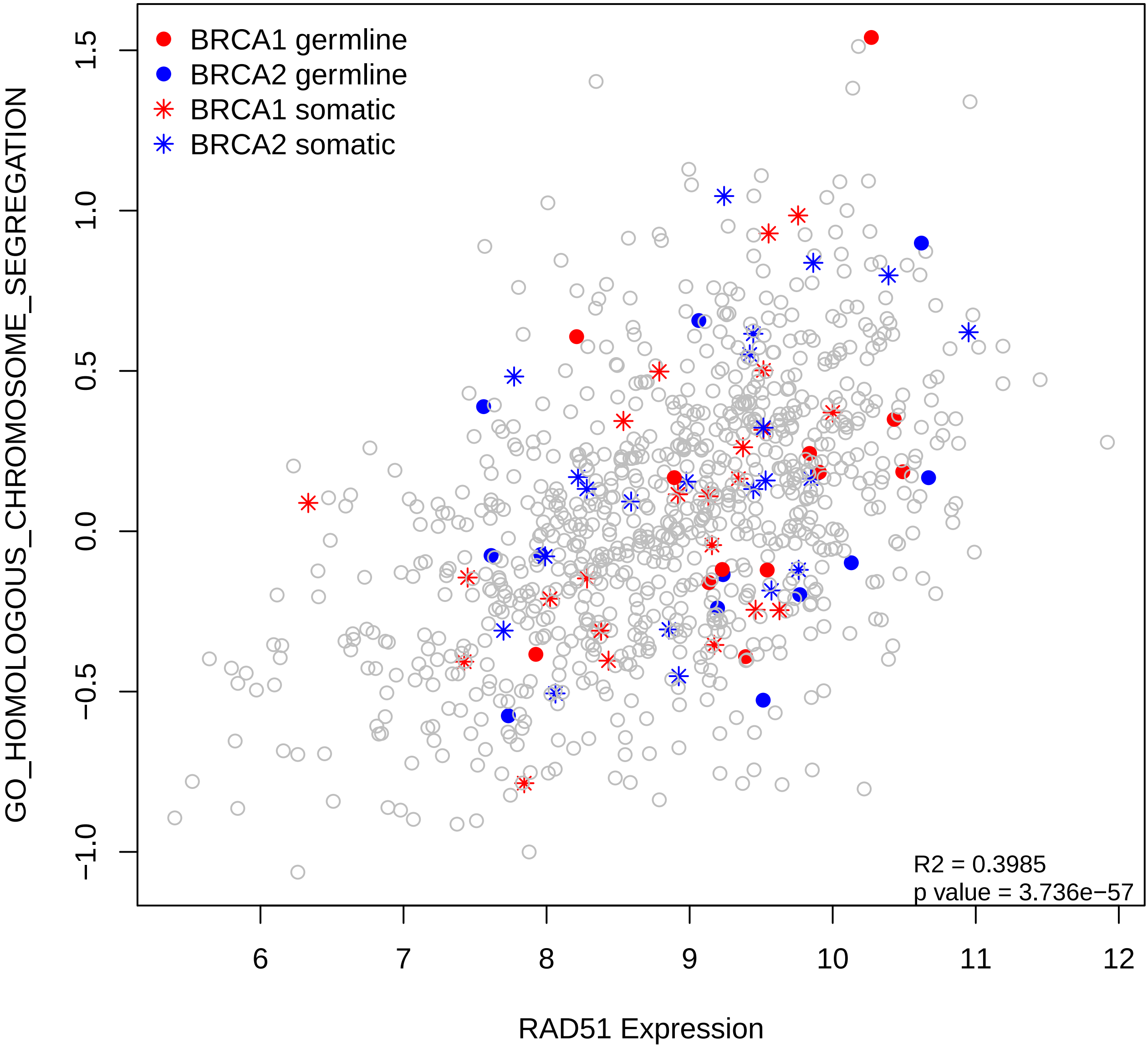

BRCA1 germline
BRCA2 germline
BRCA1 somatic
BRCA2 somatic
R2 = 0.3985
p value = 3.736e−57
GO_HOMOLOGOUS_CHROMOSOME_SEGREGATION
RAD51 Expression
1.5
1.0
0.5
0.0
−0.5
−1.0
6
7
8
9
10
11
12

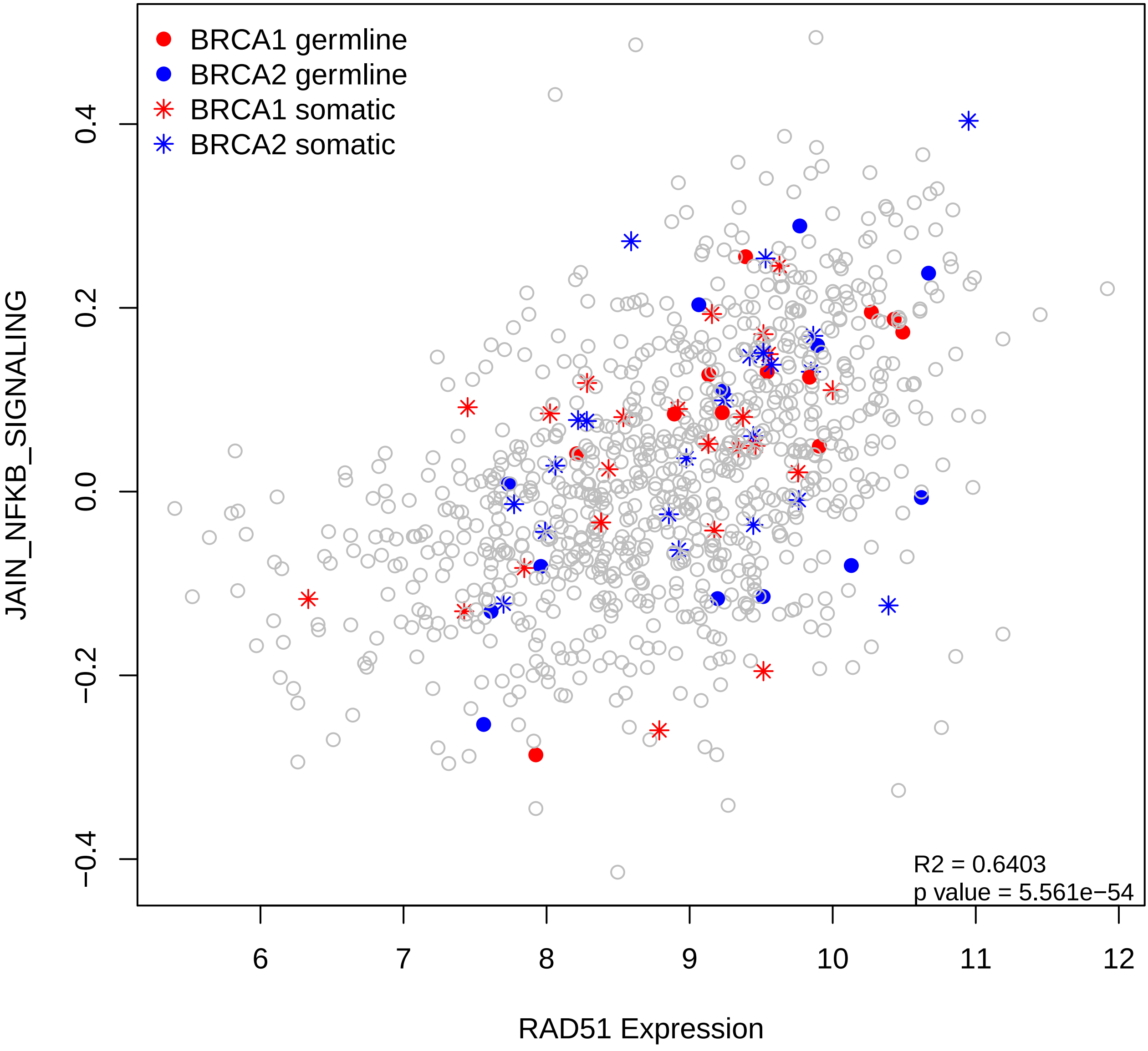

BRCA1 germline
BRCA2 germline
BRCA1 somatic
BRCA2 somatic
R2 = 0.6403
p value = 5.561e−54
JAIN_NFKB_SIGNALING
RAD51 Expression
0.4
0.2
0.0
−0.2
−0.4
6
7
8
9
10
11
12

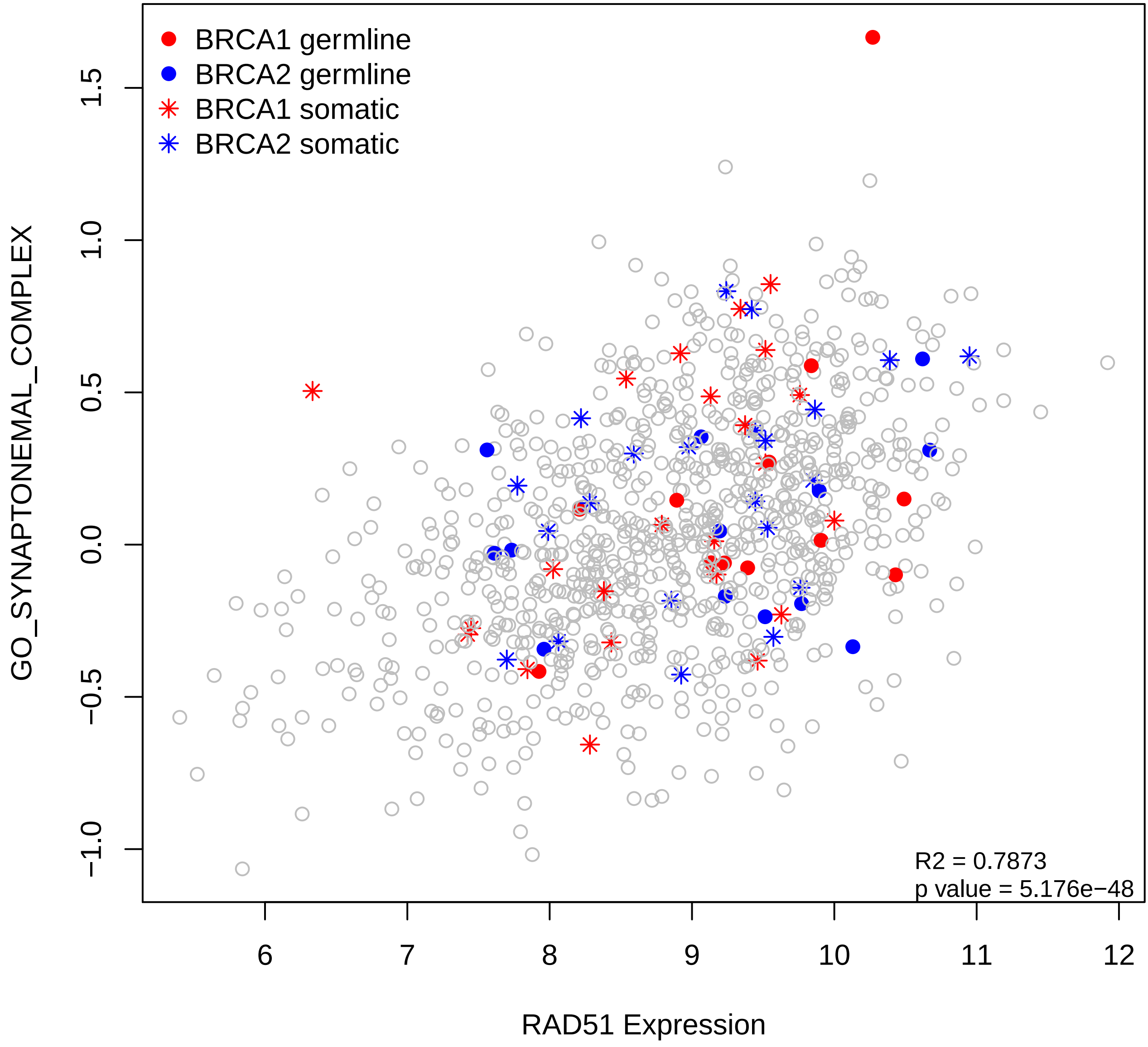

BRCA1 germline
BRCA2 germline
BRCA1 somatic
BRCA2 somatic
GO_SYNAPTONEMAL_COMPLEX
RAD51 Expression
R2 = 0.7873
p value = 5.176e−48
1.5
1.0
0.5
0.0
−0.5
−1.0
6
7
8
9
10
11
12

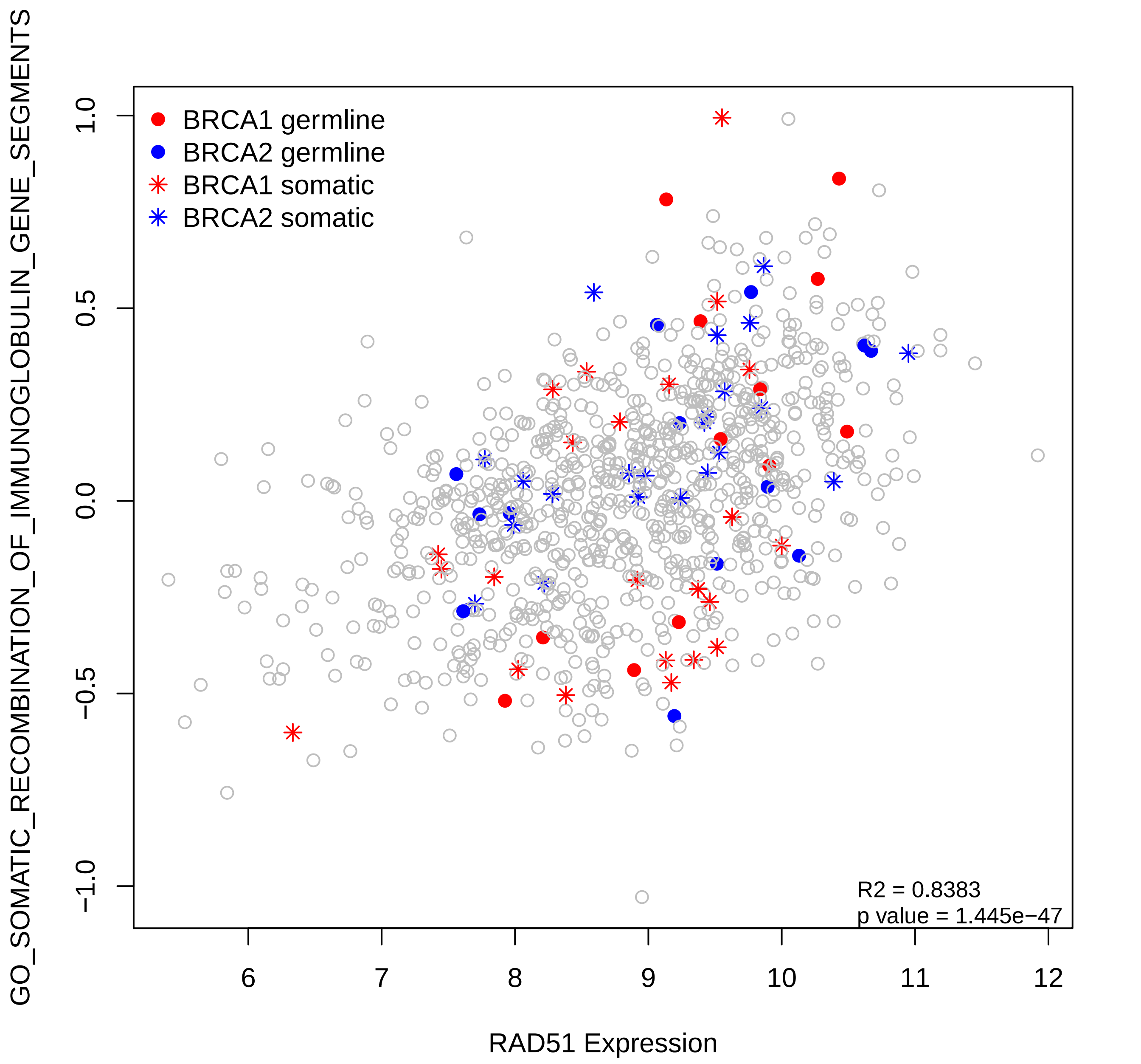

BRCA1 germline
BRCA2 germline
BRCA1 somatic
BRCA2 somatic
R2 = 0.8383
p value = 1.445e−47
GO_SOMATIC_RECOMBINATION_OF_IMMUNOGLOBULIN_GENE_SEGMENTS
RAD51 Expression
1.0
0.5
0.0
−0.5
−1.0
6
7
8
9
10
11
12

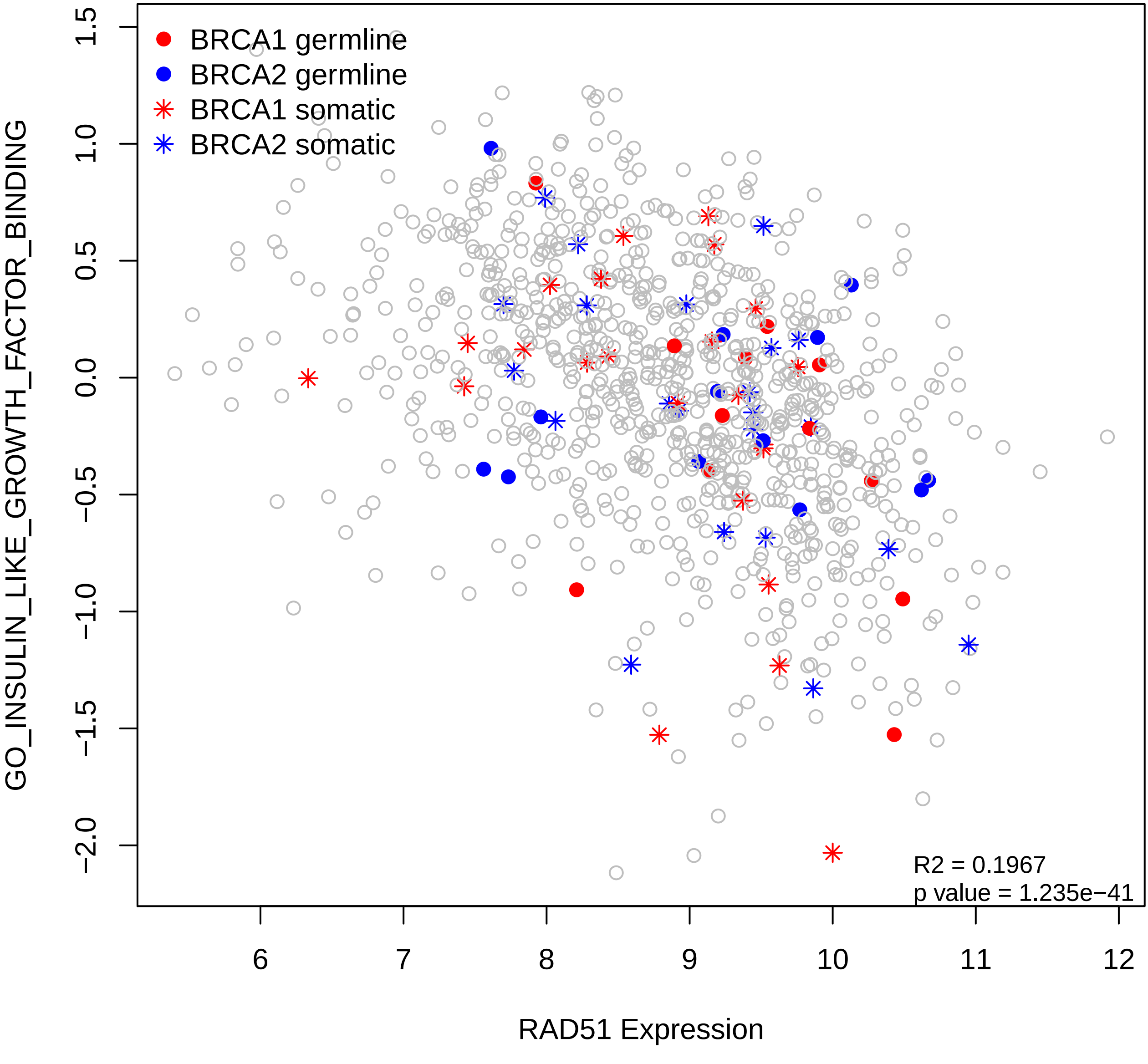

BRCA1 germline
BRCA2 germline
BRCA1 somatic
BRCA2 somatic
GO_INSULIN_LIKE_GROWTH_FACTOR_BINDING
RAD51 Expression
R2 = 0.1967
p value = 1.235e−41
1.5
1.0
0.5
0.0
−0.5
−1.0
−1.5
−2.0
6
7
8
9
10
11
12

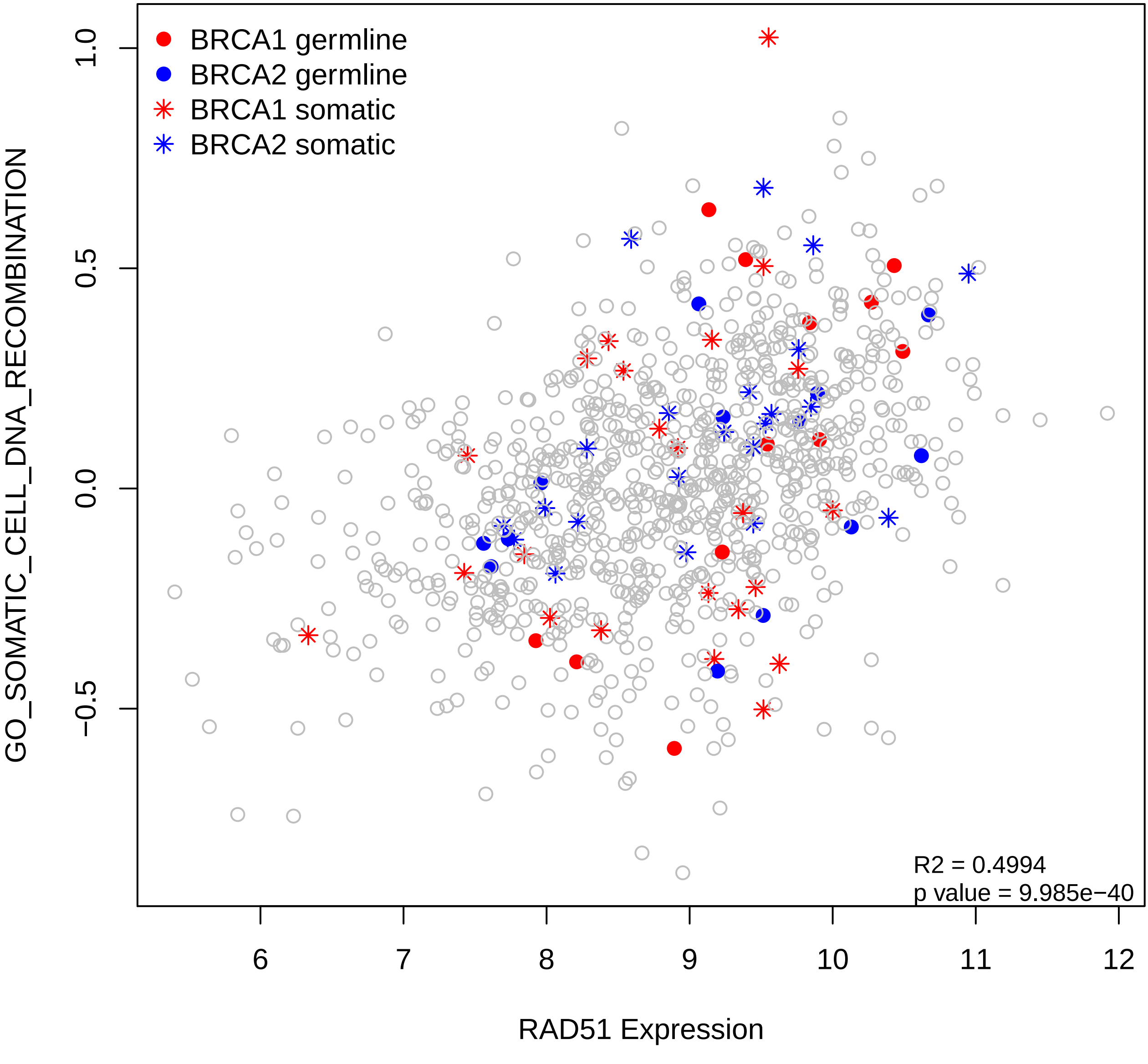

BRCA1 germline
BRCA2 germline
BRCA1 somatic
BRCA2 somatic
R2 = 0.4994
p value = 9.985e−40
GO_SOMATIC_CELL_DNA_RECOMBINATION
RAD51 Expression
−0.5
0.0
0.5
1.0
6
7
8
9
10
11
12

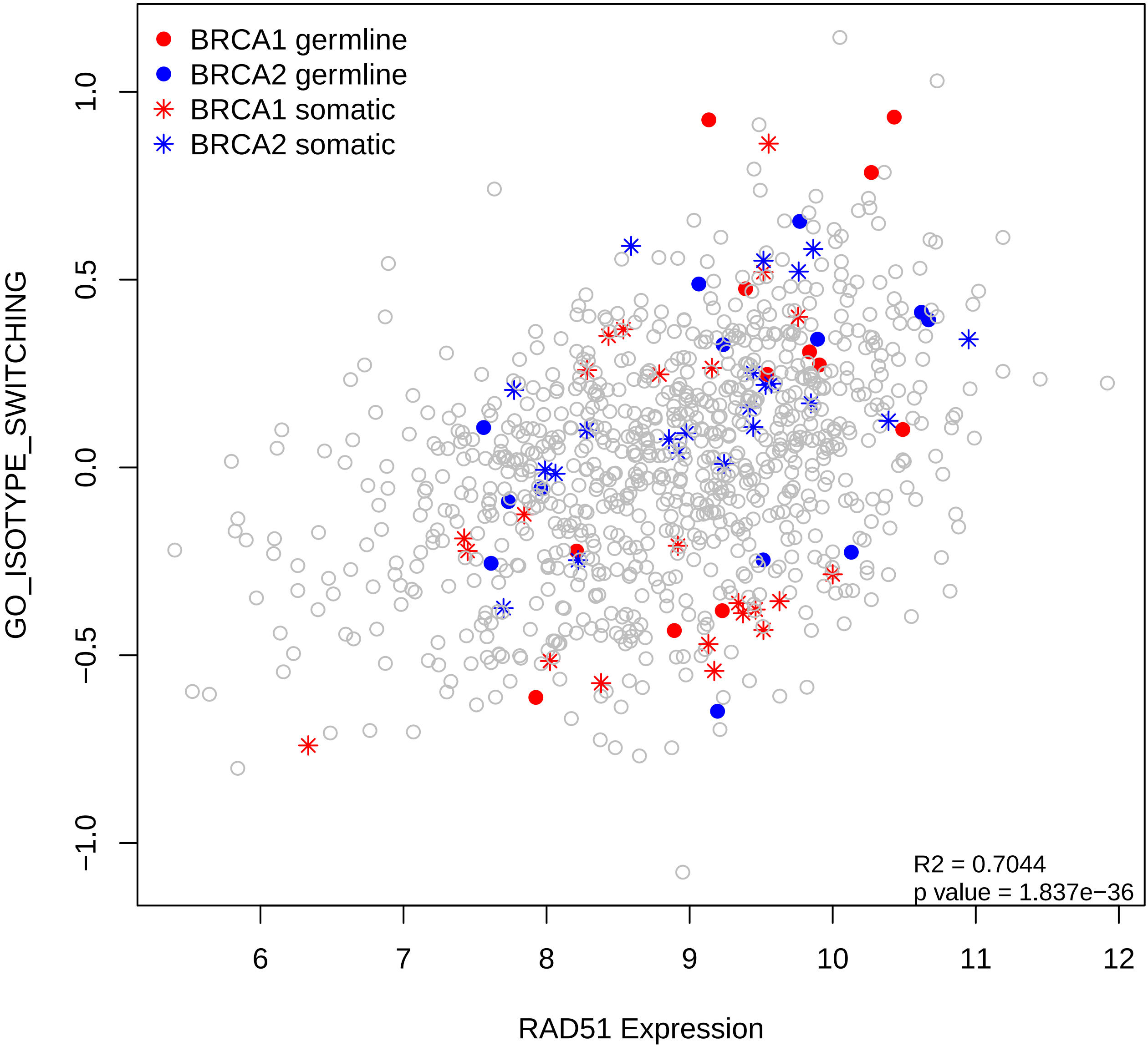

BRCA1 germline
BRCA2 germline
BRCA1 somatic
BRCA2 somatic
GO_ISOTYPE_SWITCHING
1.0
0.5
0.0
−0.5
−1.0
6
7
8
9
10
11
12
RAD51 Expression
R2 = 0.7044
p value = 1.837e−36

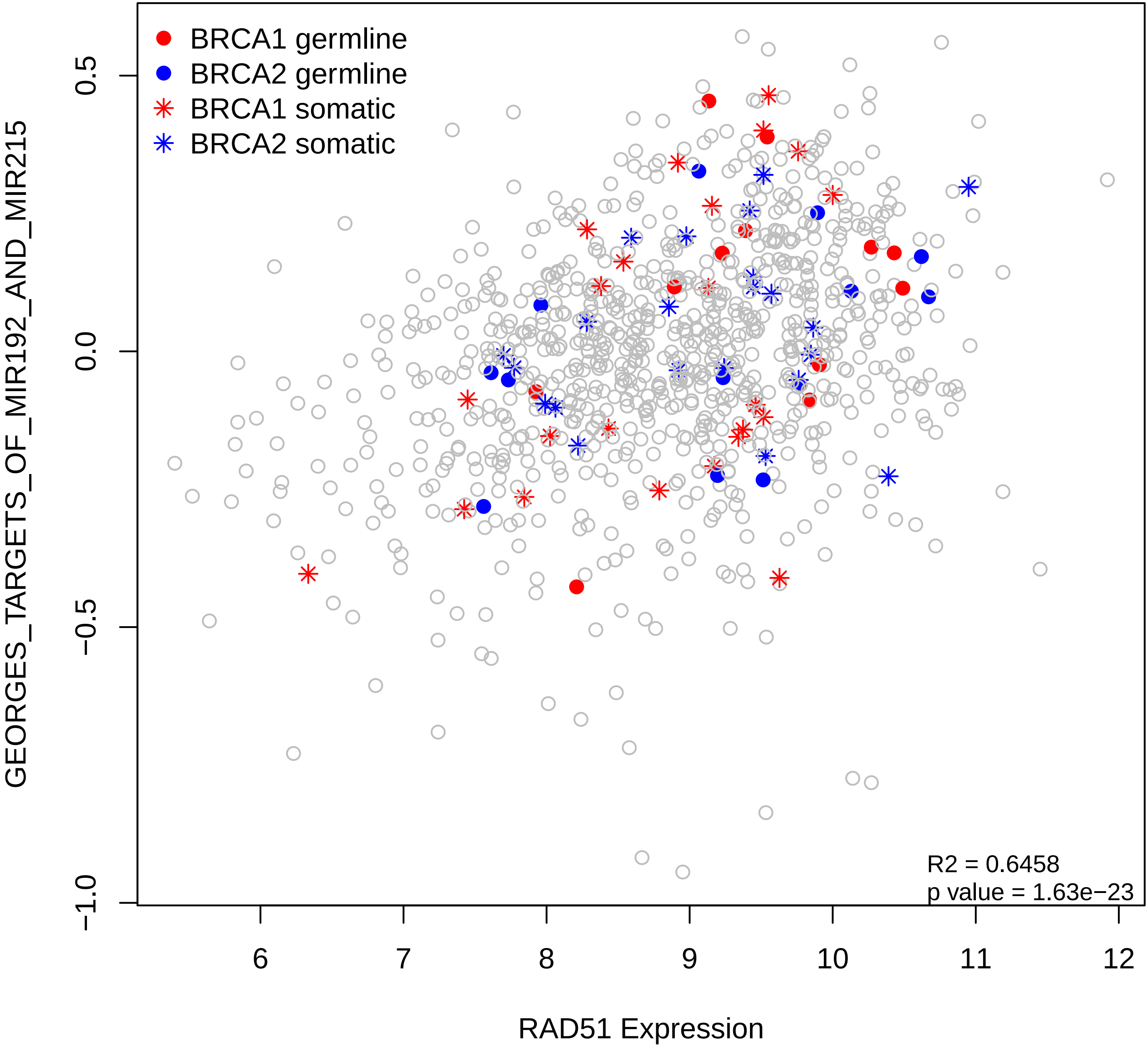

BRCA1 germline
BRCA2 germline
BRCA1 somatic
BRCA2 somatic
R2 = 0.6458
p value = 1.63e−23
GEORGES_TARGETS_OF_MIR192_AND_MIR215
RAD51 Expression
0.5
0.0
−0.5
−1.0
6
7
8
9
10
11
12

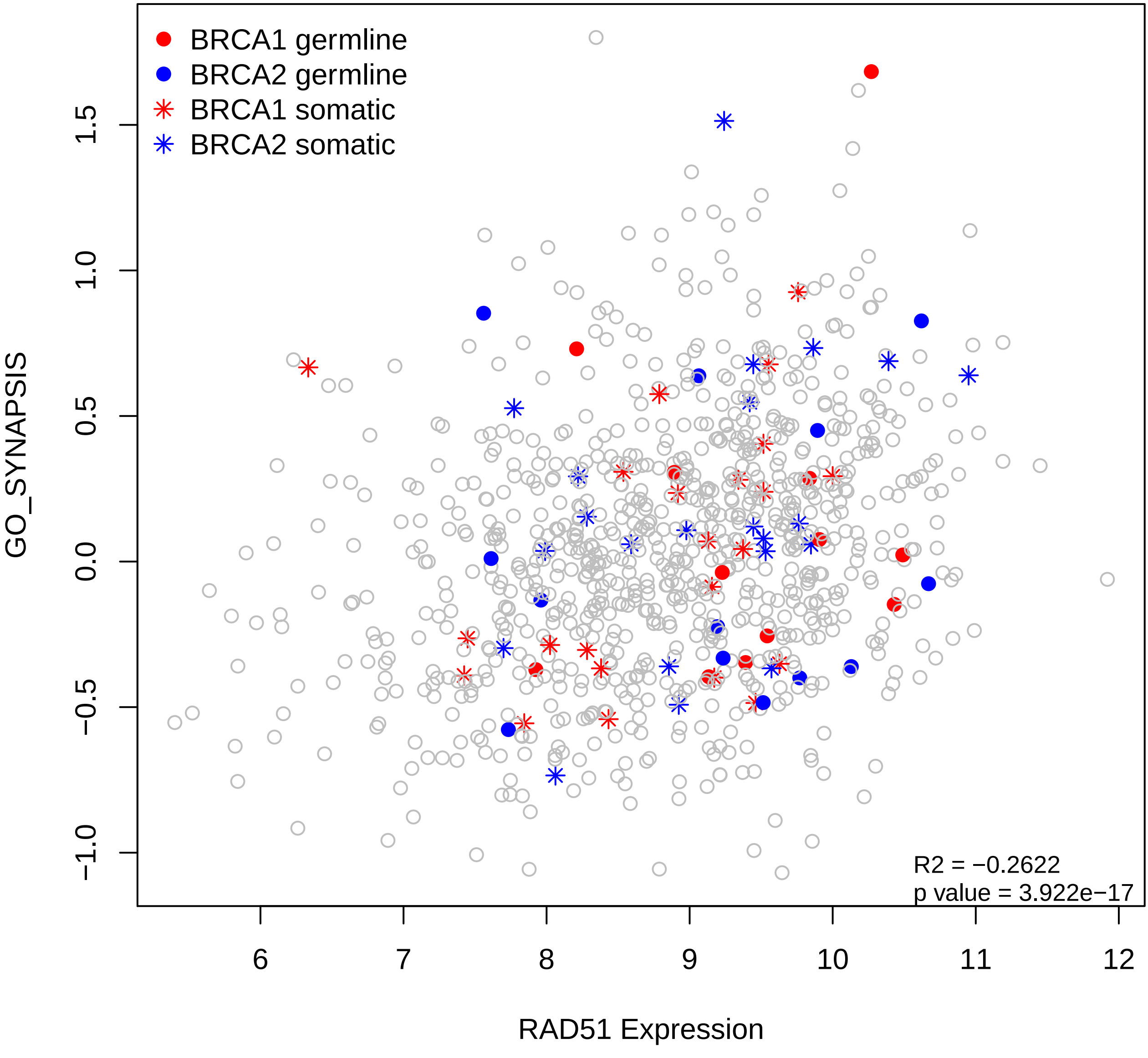

BRCA1 germline
BRCA2 germline
BRCA1 somatic
BRCA2 somatic
GO_SYNAPSIS
−1.0
−0.5
0.0
0.5
1.0
1.5
6
7
8
9
10
11
12
RAD51 Expression
R2 = −0.2622
p value = 3.922e−17

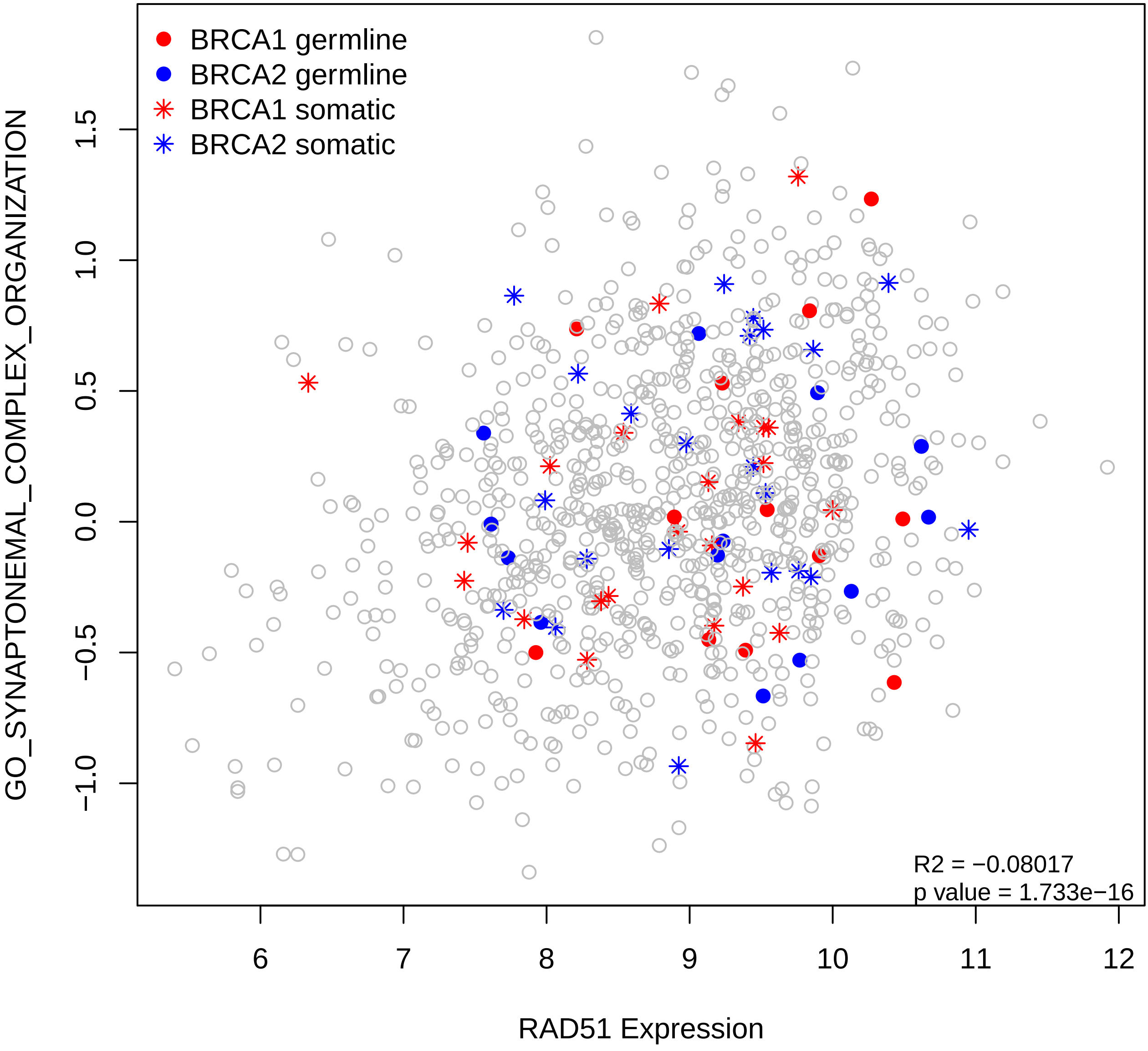

BRCA1 germline
BRCA2 germline
BRCA1 somatic
BRCA2 somatic
GO_SYNAPTONEMAL_COMPLEX_ORGANIZATION
RAD51 Expression
R2 = −0.08017
p value = 1.733e−16
−1.0
−0.5
0.0
0.5
1.0
1.5
6
7
8
9
10
11
12

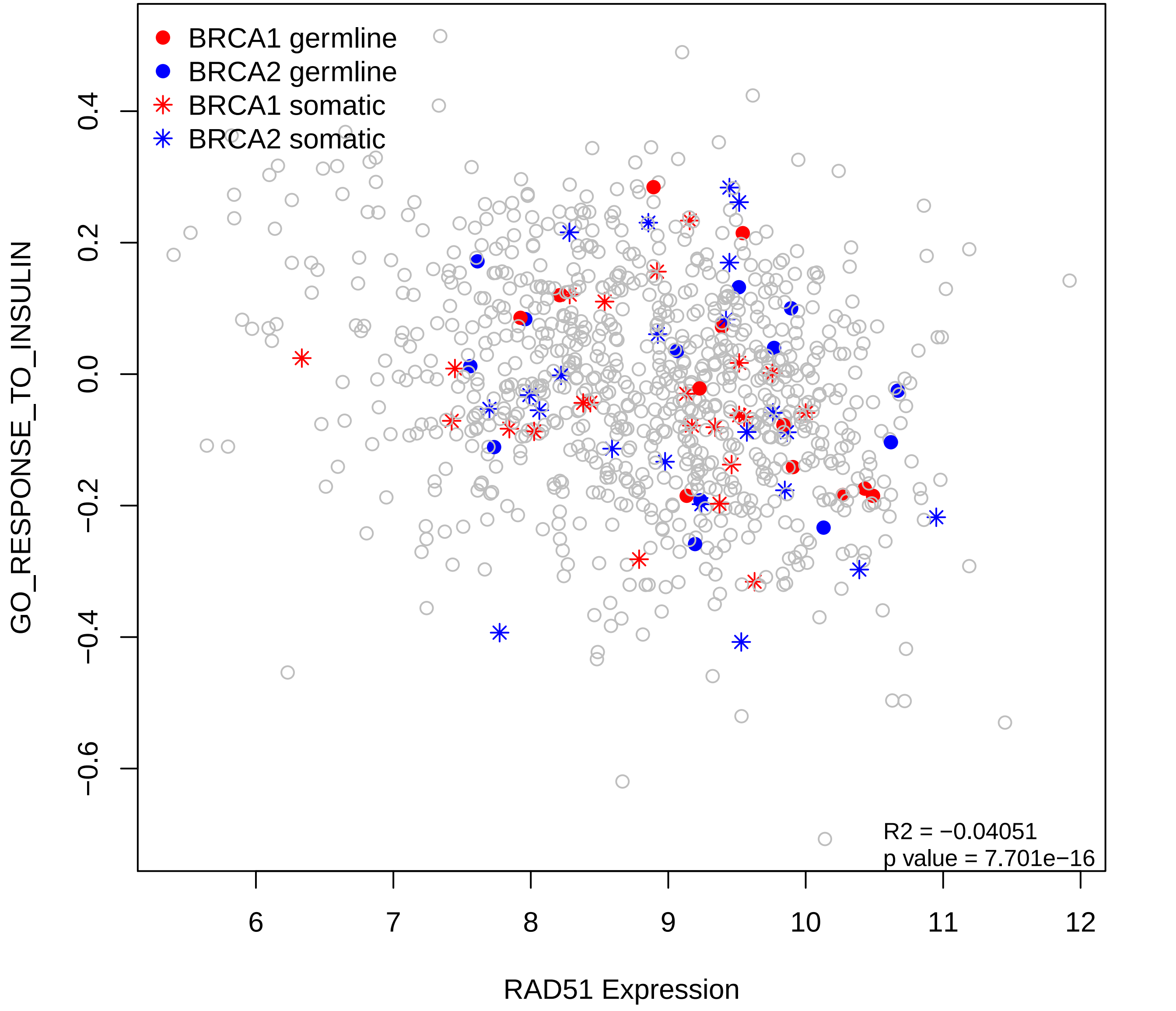
BRCA1 germline
BRCA2 germline
BRCA1 somatic
BRCA2 somatic
GO_RESPONSE_TO_INSULIN
RAD51 Expression
R2 = −0.04051
p value = 7.701e−16
0.4
0.2
0.0
−0.2
−0.4
−0.6
6
7
8
9
10
11
12

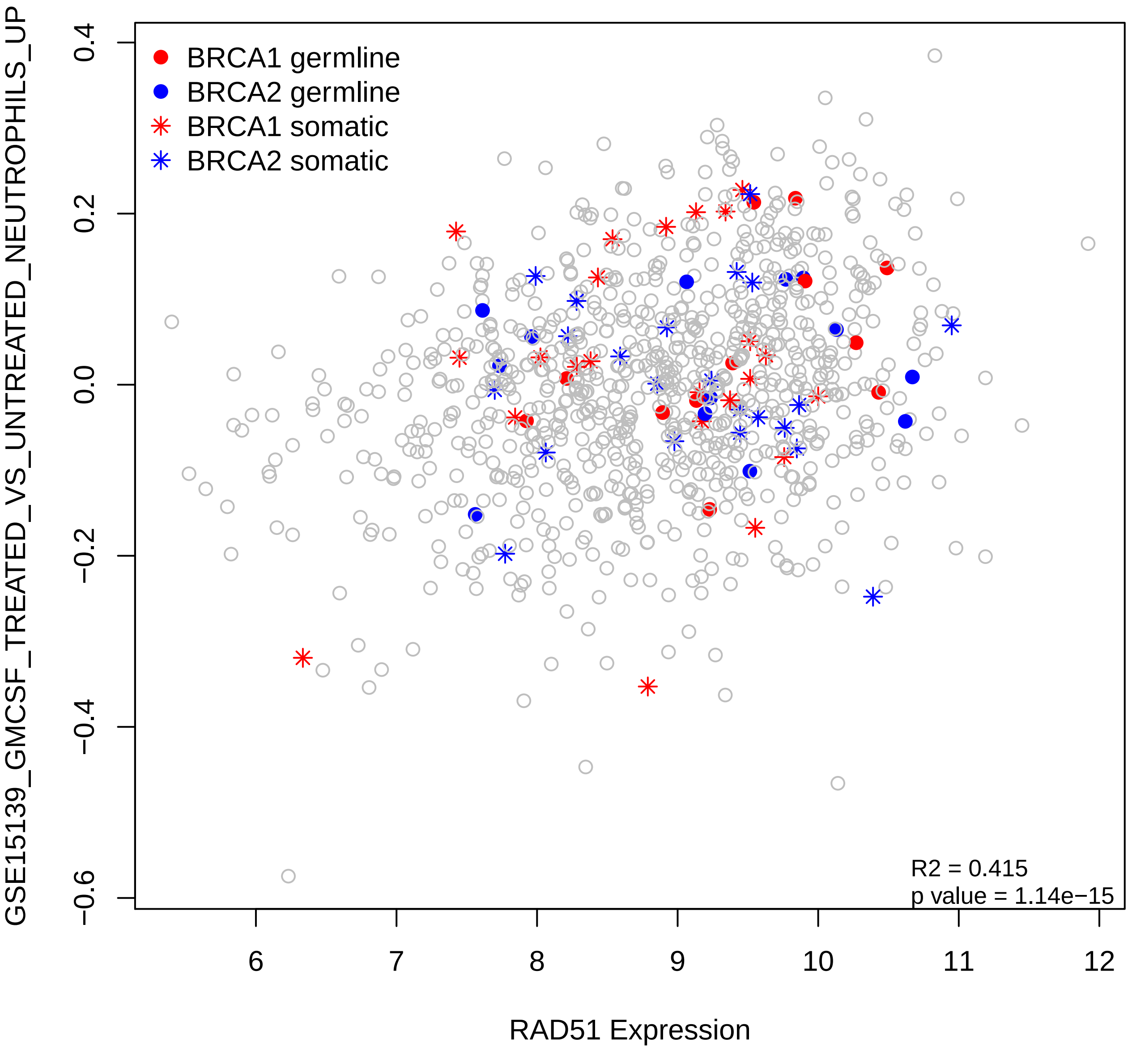

BRCA1 germline
BRCA2 germline
BRCA1 somatic
BRCA2 somatic
R2 = 0.415
p value = 1.14e−15
GSE15139_GMCSF_TREATED_VS_UNTREATED_NEUTROPHILS_UP
RAD51 Expression
0.4
0.2
0.0
−0.2
−0.4
−0.6
6
7
8
9
10
11
12

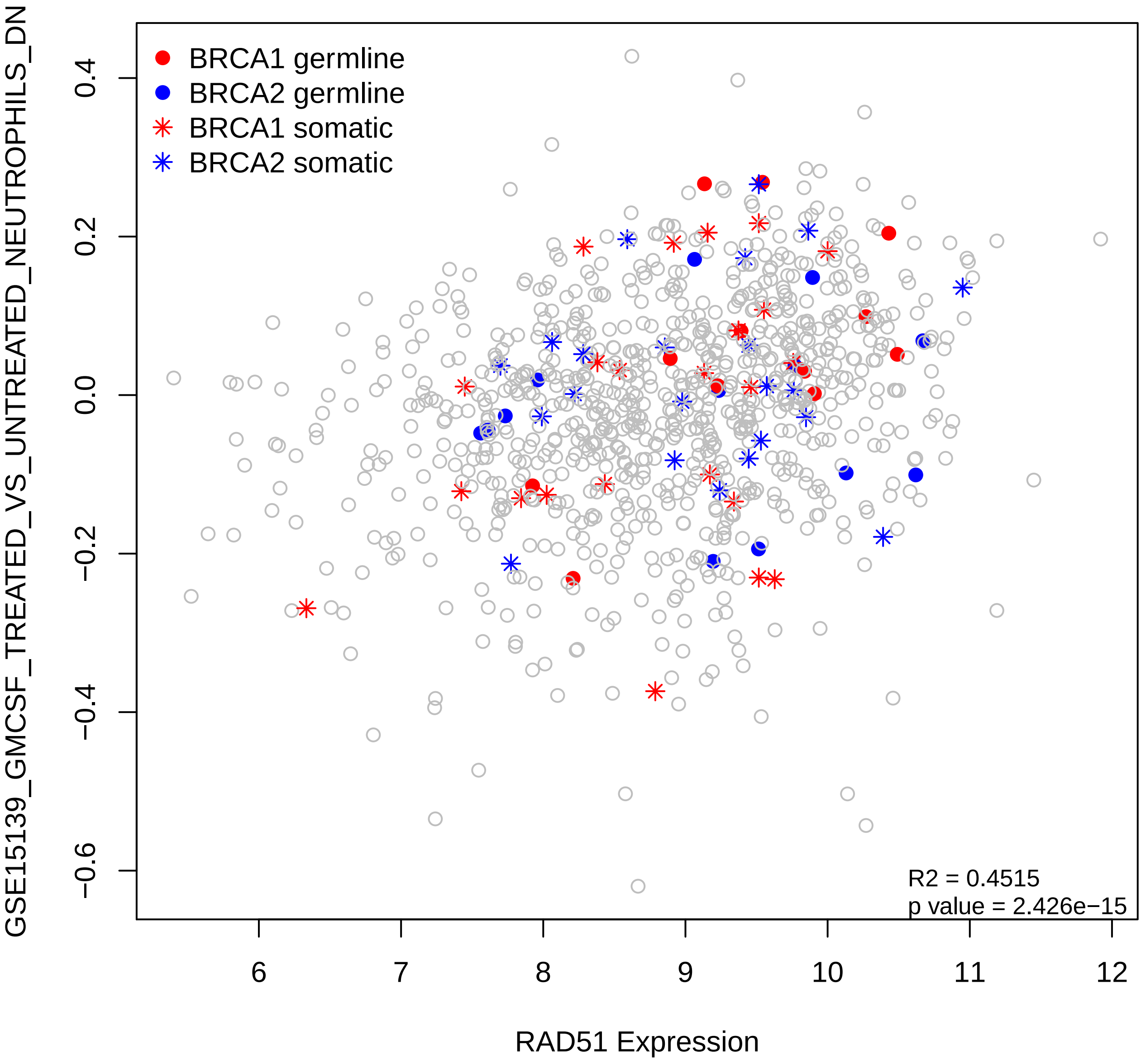

BRCA1 germline
BRCA2 germline
BRCA1 somatic
BRCA2 somatic
R2 = 0.4515
p value = 2.426e−15
GSE15139_GMCSF_TREATED_VS_UNTREATED_NEUTROPHILS_DN
0.4
0.2
0.0
−0.2
−0.4
−0.6
6
7
8
9
10
11
12
RAD51 Expression

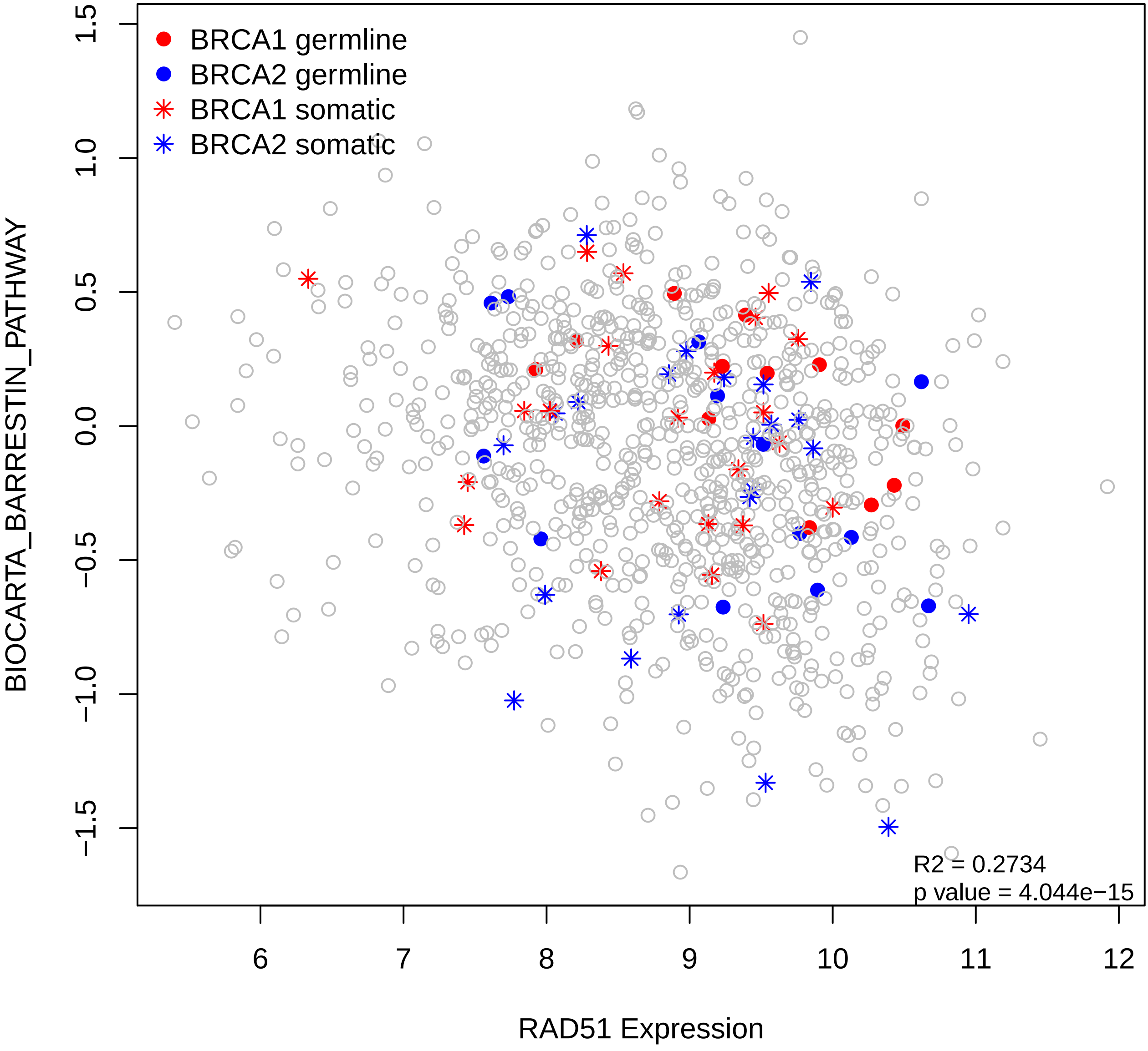
BRCA1 germline
BRCA2 germline
BRCA1 somatic
BRCA2 somatic
R2 = 0.2734
p value = 4.044e−15
BIOCARTA_BARRESTIN_PATHWAY
RAD51 Expression
1.5
1.0
0.5
0.0
−0.5
−1.0
−1.5
6
7
8
9
10
11
12

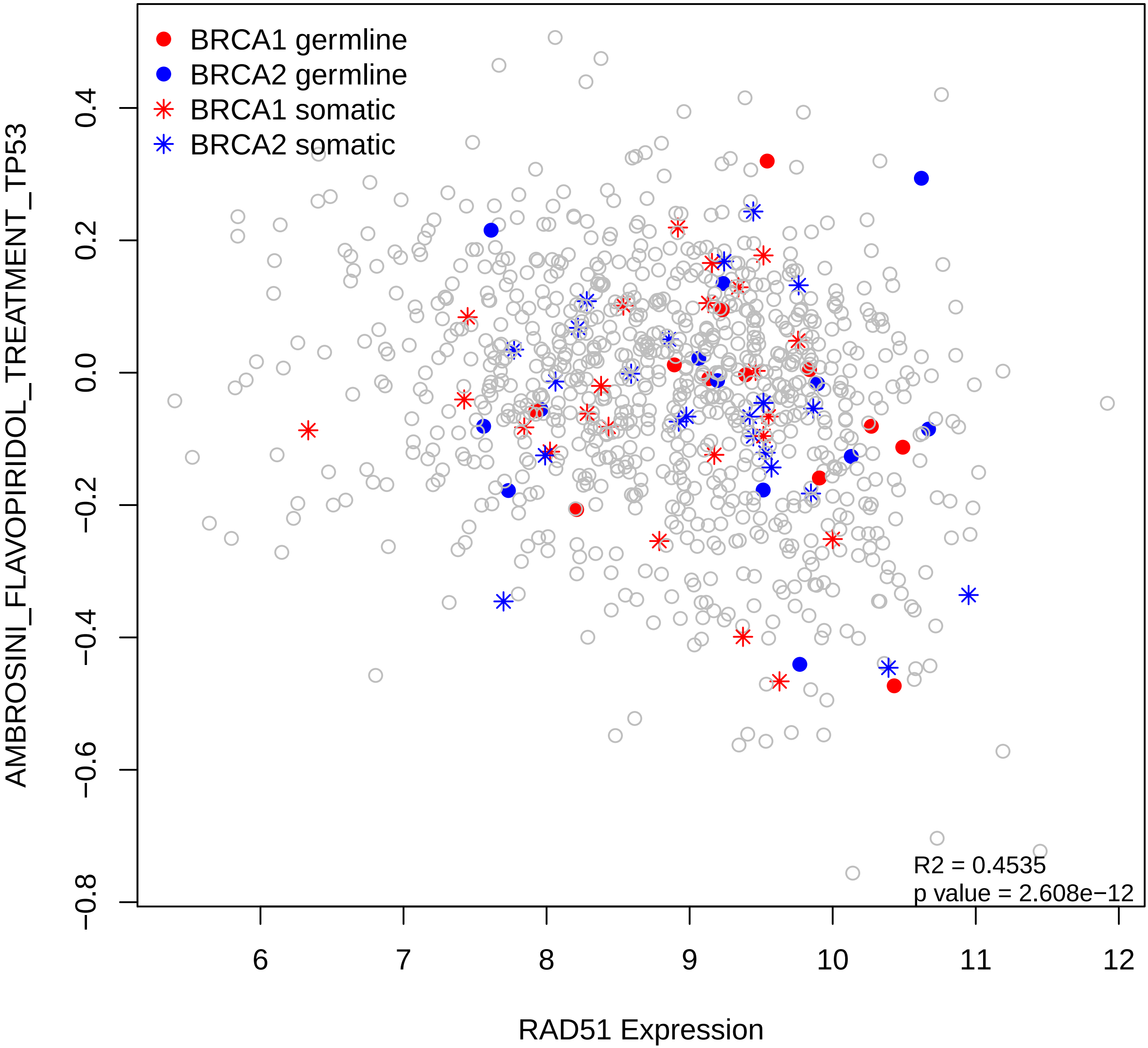

BRCA1 germline
BRCA2 germline
BRCA1 somatic
BRCA2 somatic
R2 = 0.4535
p value = 2.608e−12
AMBROSINI_FLAVOPIRIDOL_TREATMENT_TP53
RAD51 Expression
0.4
0.2
0.0
−0.2
−0.4
−0.6
−0.8
6
7
8
9
10
11
12

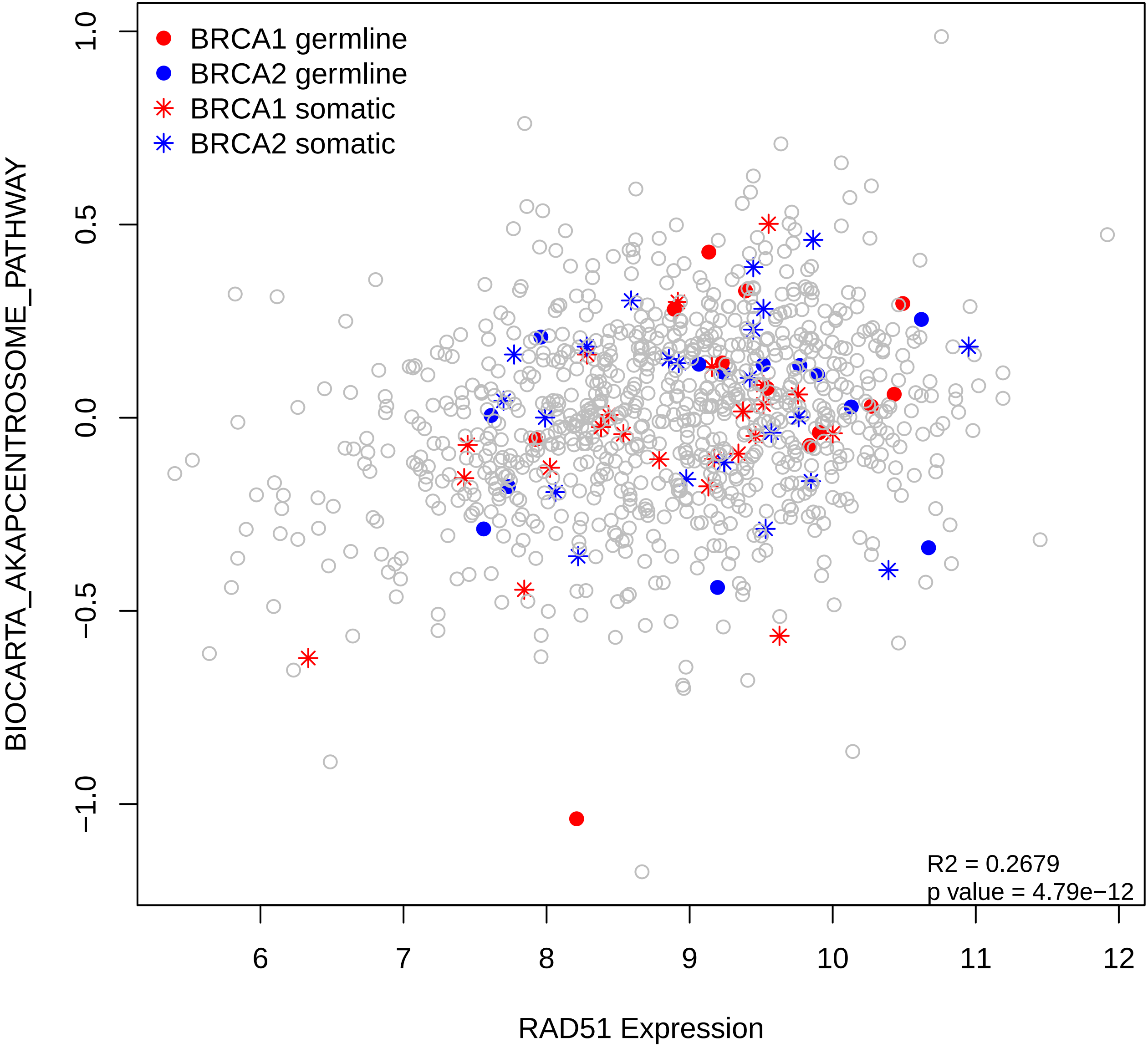
BRCA1 germline
BRCA2 germline
BRCA1 somatic
BRCA2 somatic
BIOCARTA_AKAPCENTROSOME_PATHWAY
RAD51 Expression
R2 = 0.2679
p value = 4.79e−12
1.0
0.5
0.0
−0.5
−1.0
6
7
8
9
10
11
12

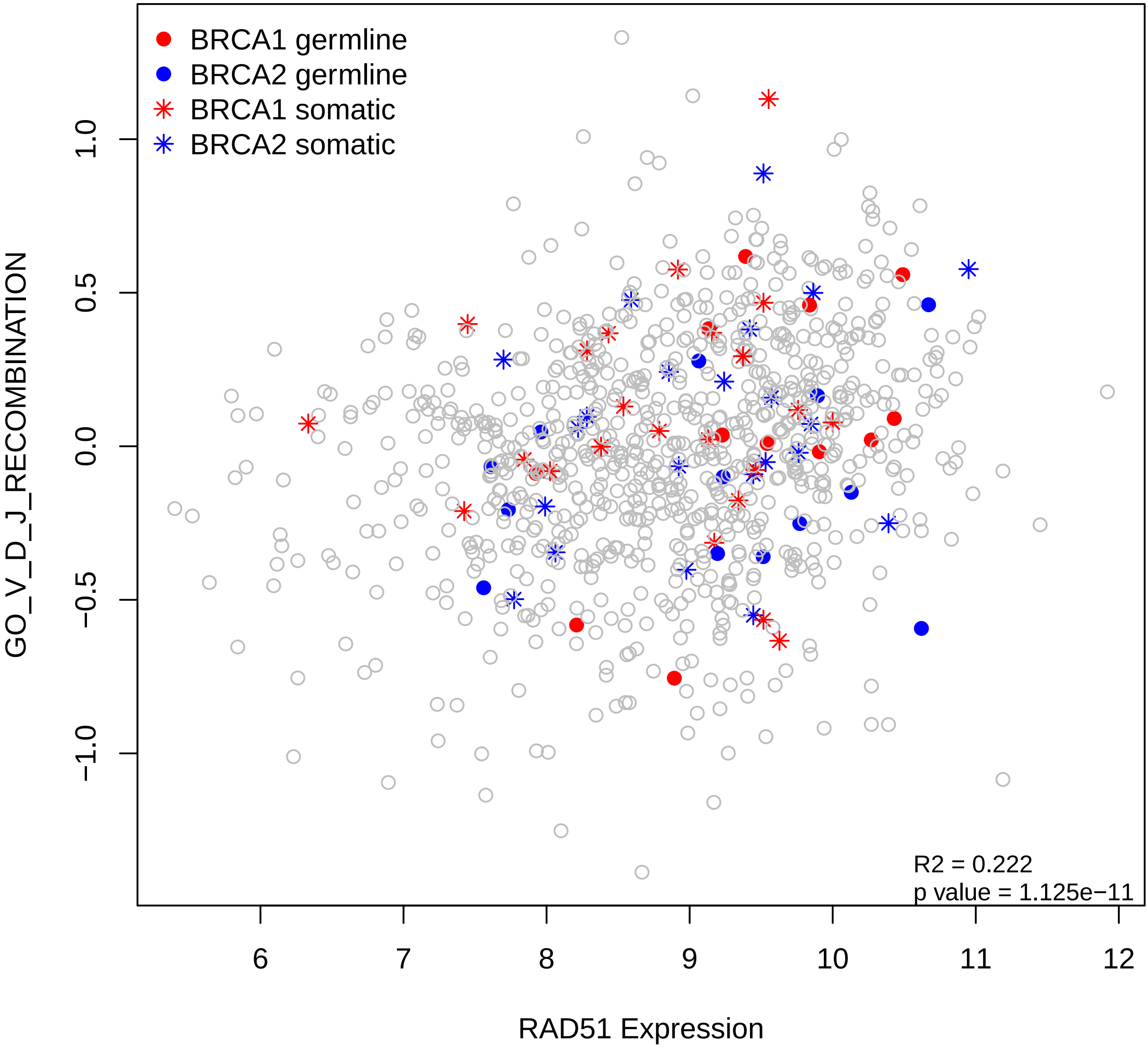
BRCA1 germline
BRCA2 germline
BRCA1 somatic
BRCA2 somatic
R2 = 0.222
p value = 1.125e−11
GO_V_D_J_RECOMBINATION
RAD51 Expression
1.0
0.5
0.0
−0.5
−1.0
6
7
8
9
10
11
12

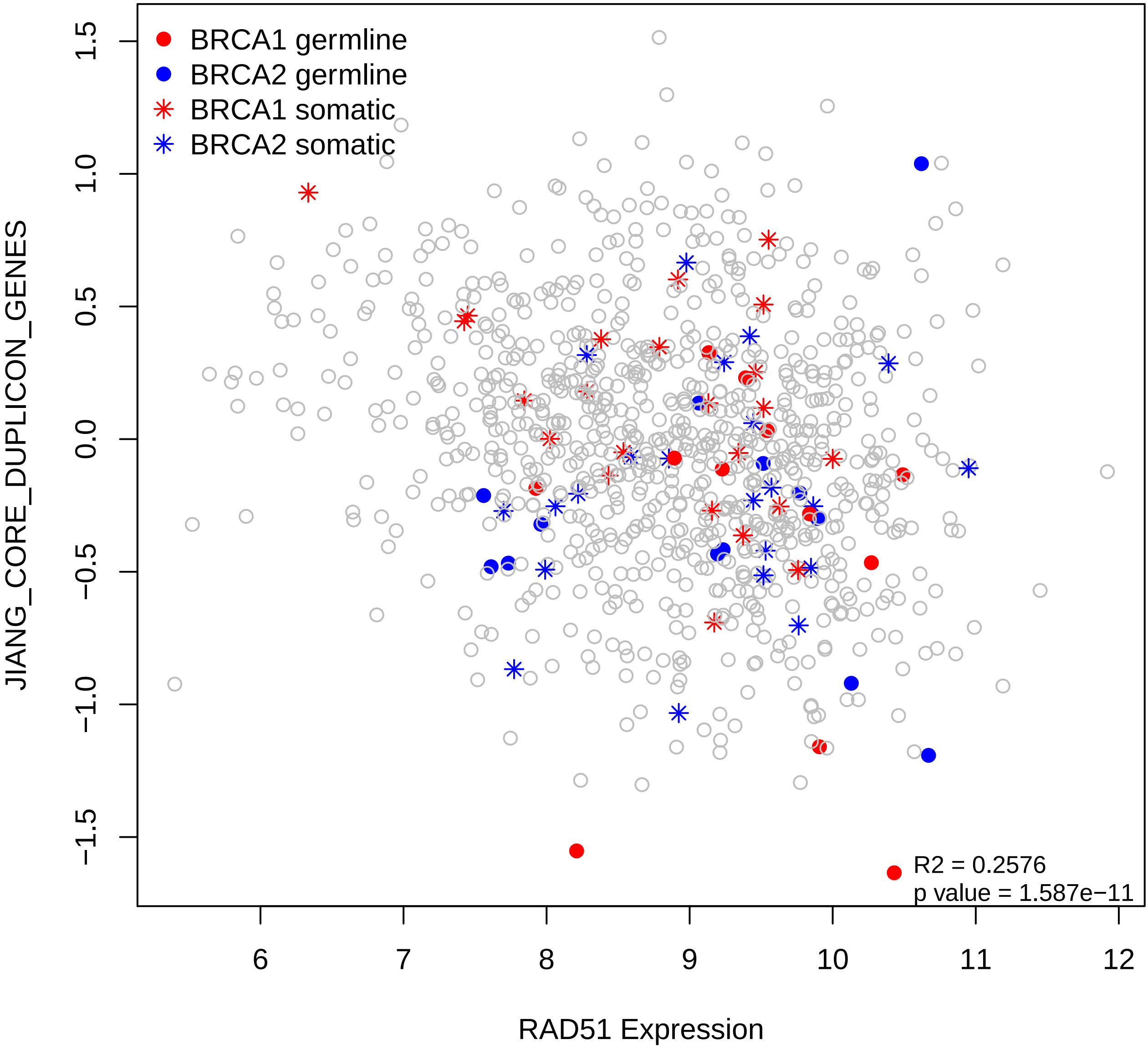

BRCA1 germline
BRCA2 germline
BRCA1 somatic
BRCA2 somatic
JIANG_CORE_DUPLICON_GENES
RAD51 Expression
R2 = 0.2576
p value = 1.587e−11
1.5
1.0
0.5
0.0
−0.5
−1.0
−1.5
6
7
8
9
10
11
12

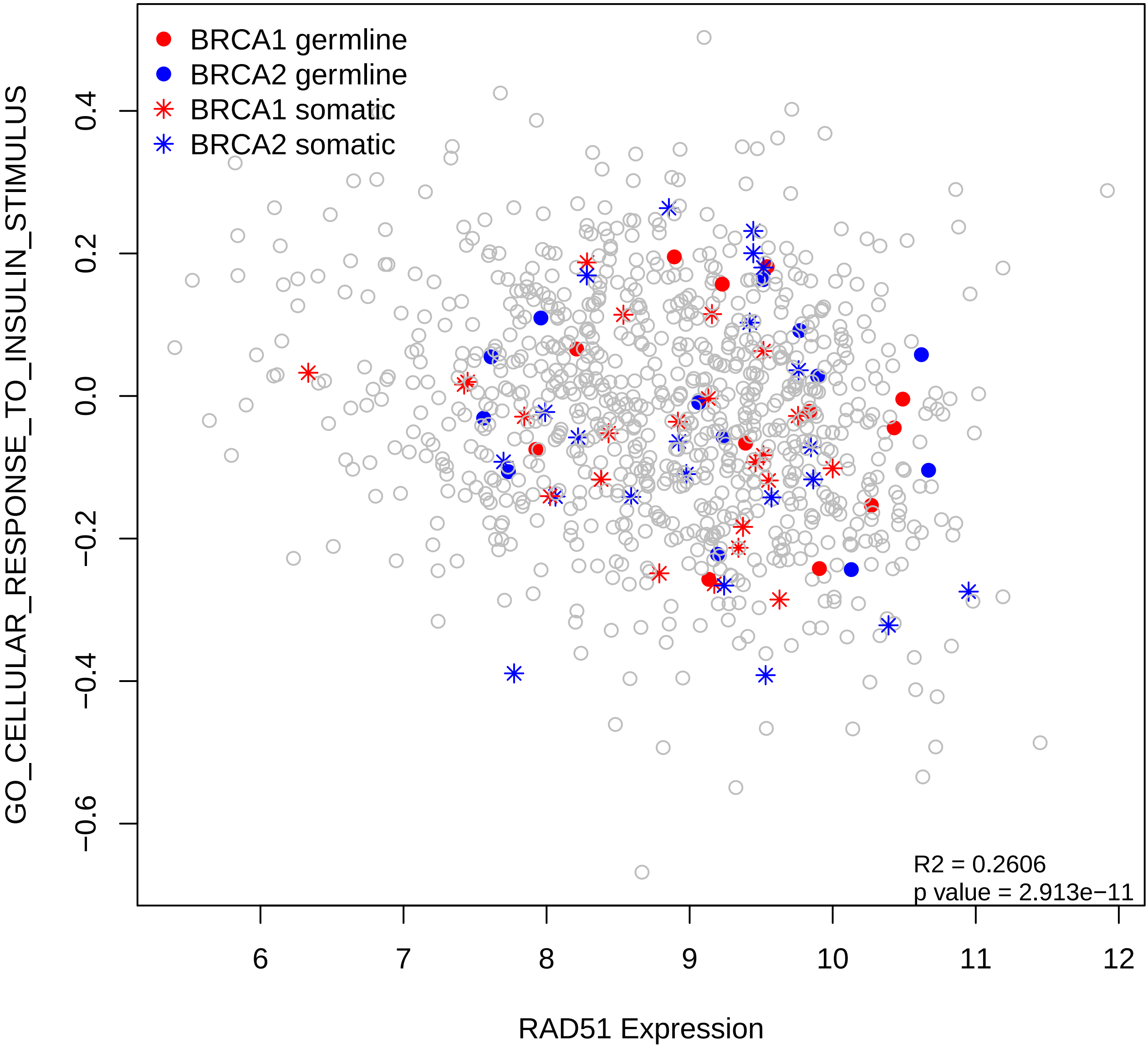

BRCA1 germline
BRCA2 germline
BRCA1 somatic
BRCA2 somatic
R2 = 0.2606
p value = 2.913e−11
GO_CELLULAR_RESPONSE_TO_INSULIN_STIMULUS
RAD51 Expression
0.4
0.2
0.0
−0.2
−0.4
−0.6
6
7
8
9
10
11
12

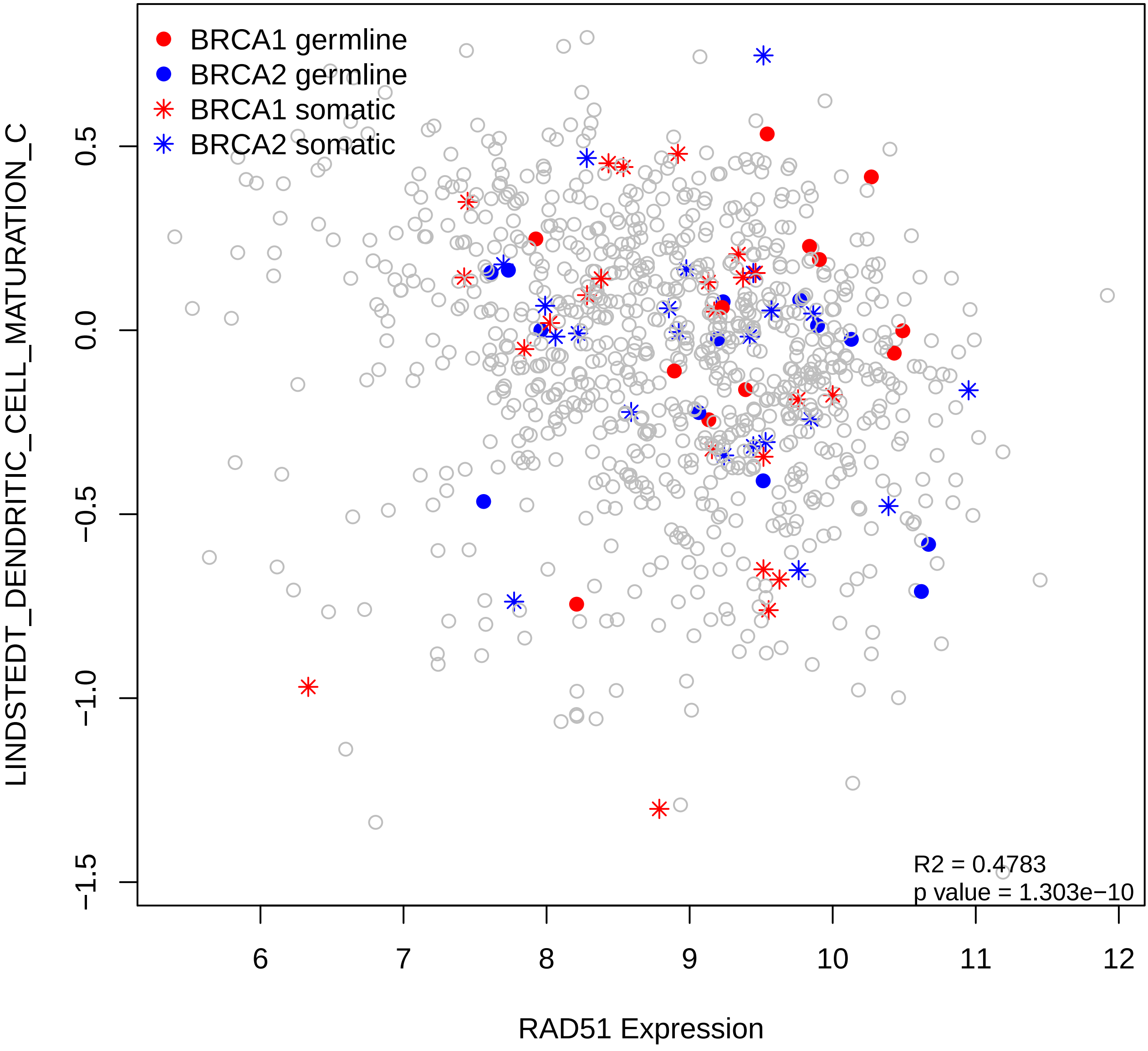

BRCA1 germline
BRCA2 germline
BRCA1 somatic
BRCA2 somatic
LINDSTEDT_DENDRITIC_CELL_MATURATION_C
0.5
0.0
−0.5
−1.0
−1.5
6
7
8
9
10
11
12
RAD51 Expression
R2 = 0.4783
p value = 1.303e−10

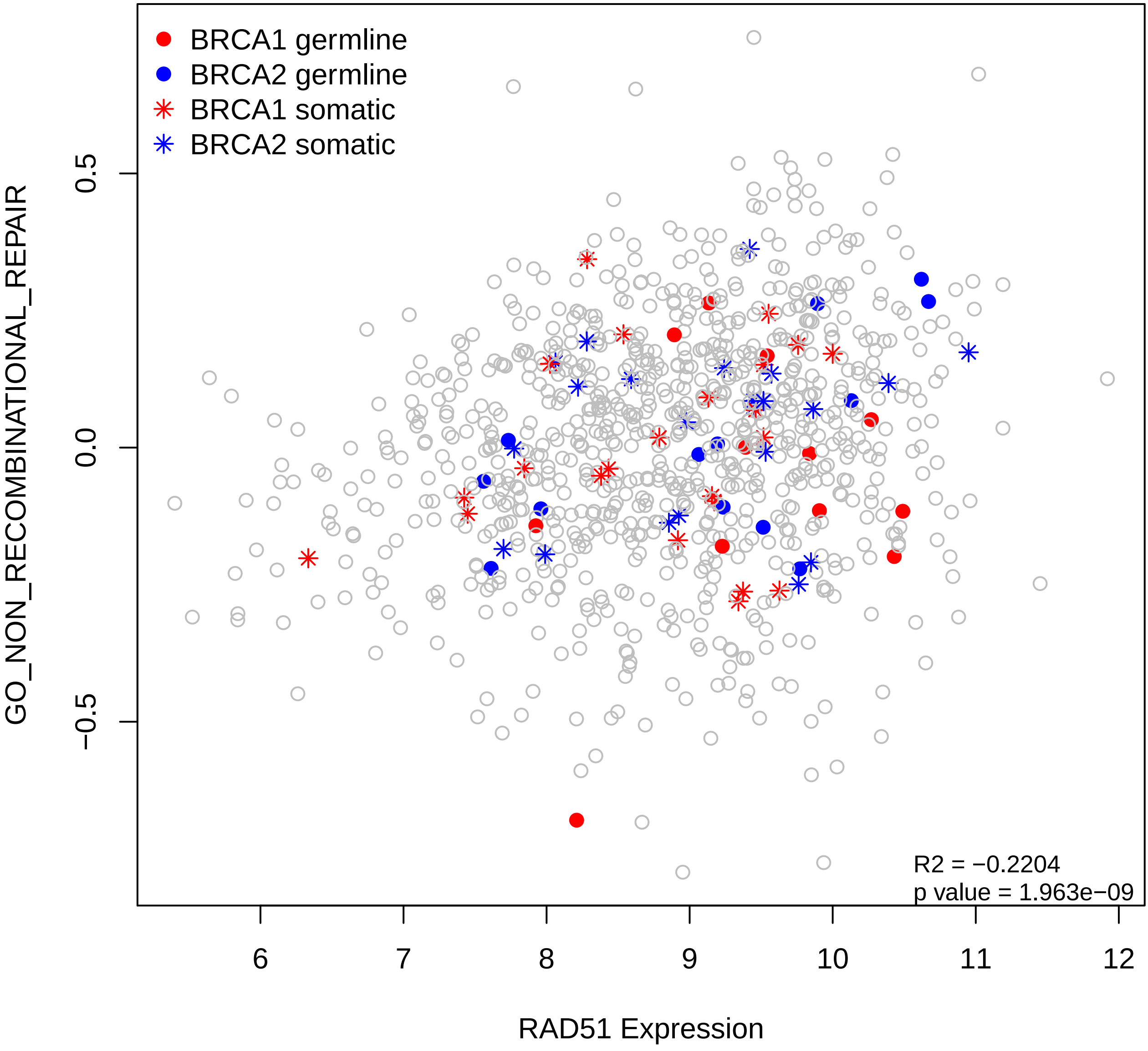

BRCA1 germline
BRCA2 germline
BRCA1 somatic
BRCA2 somatic
GO_NON_RECOMBINATIONAL_REPAIR
0.5
0.0
−0.5
6
7
8
9
10
11
12
RAD51 Expression
R2 = −0.2204
p value = 1.963e−09

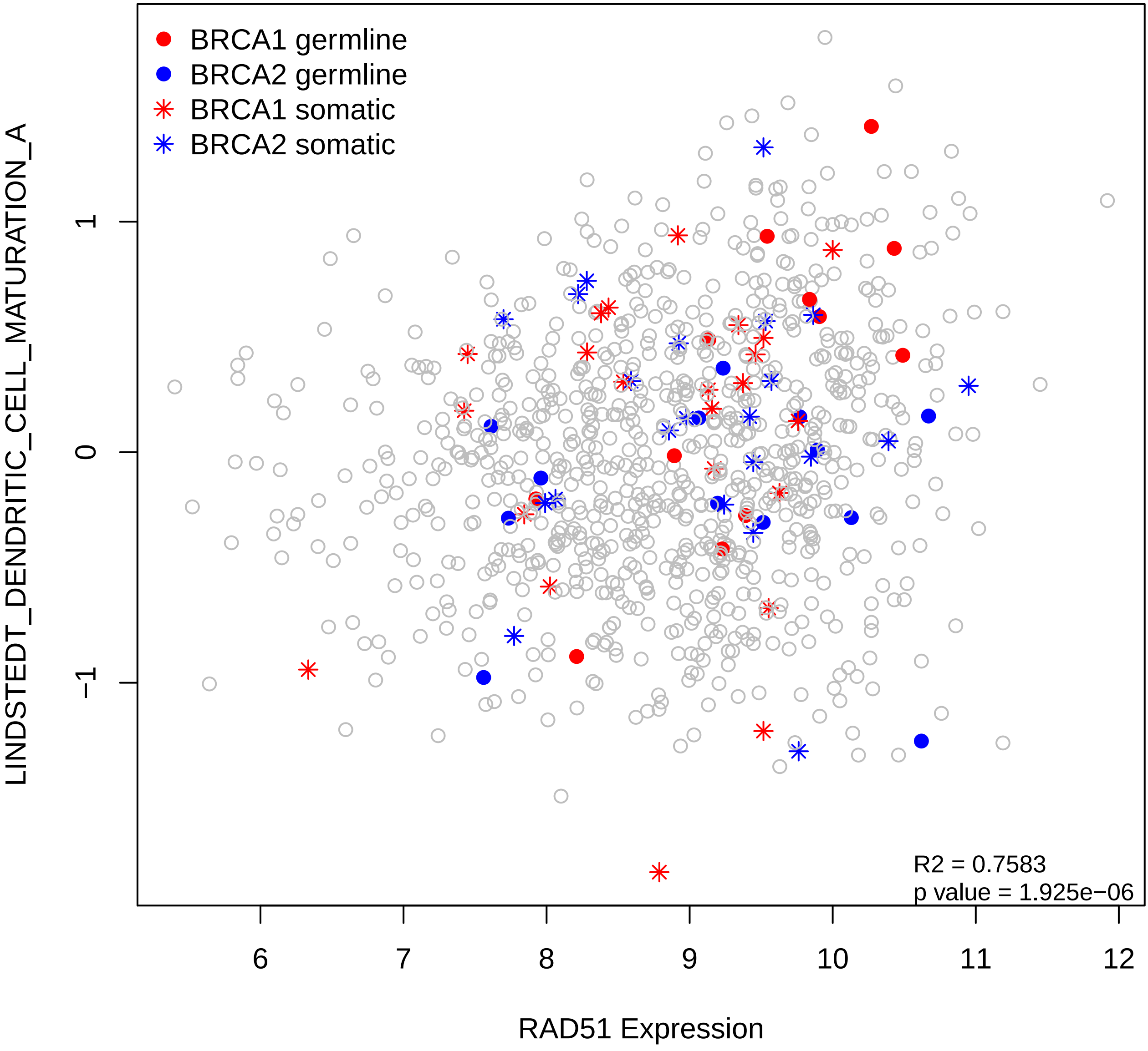

BRCA1 germline
BRCA2 germline
BRCA1 somatic
BRCA2 somatic
R2 = 0.7583
p value = 1.925e−06
LINDSTEDT_DENDRITIC_CELL_MATURATION_A
−1
0
1
6
7
8
9
10
11
12
RAD51 Expression

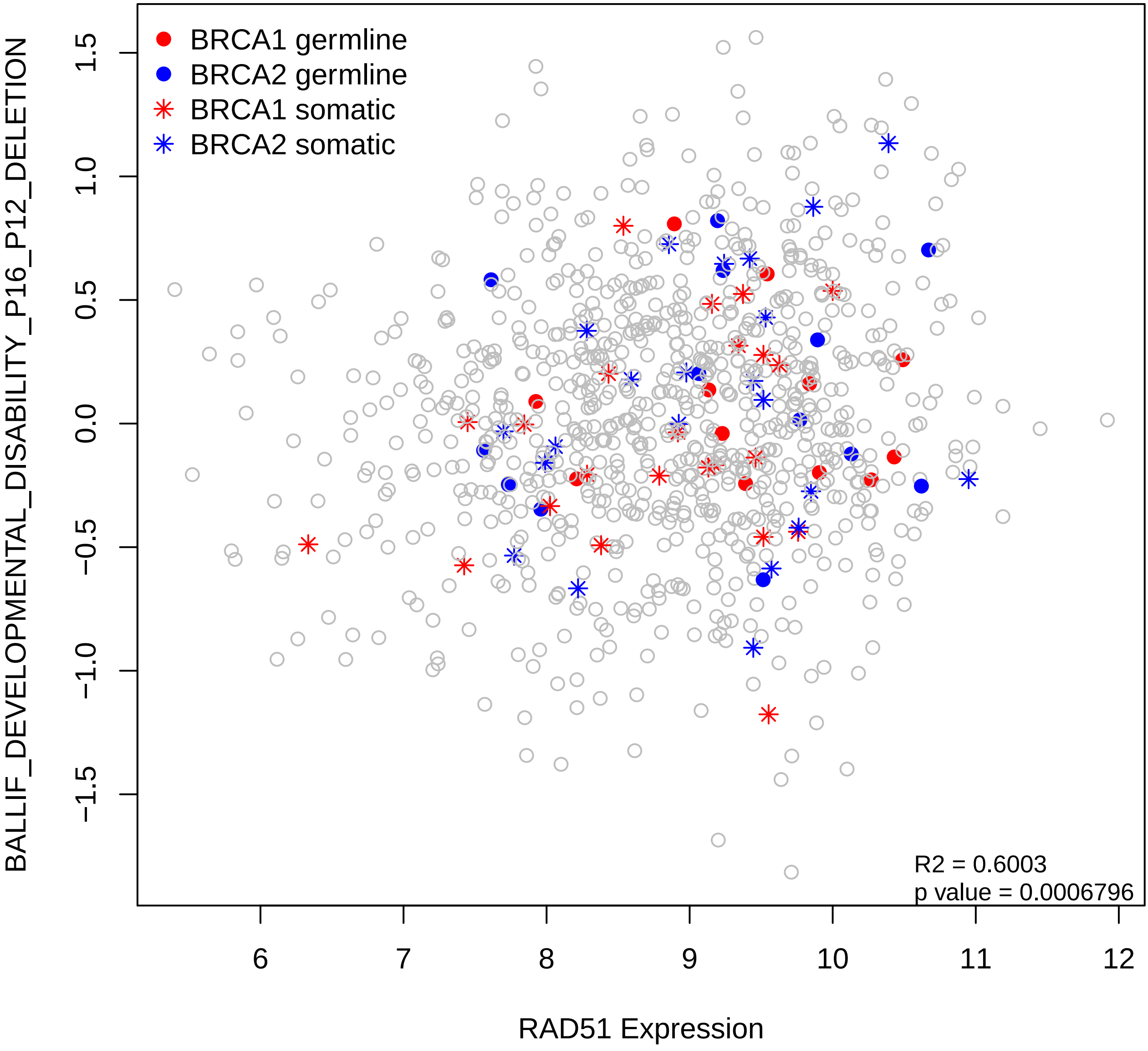
BRCA1 germline
BRCA2 germline
BRCA1 somatic
BRCA2 somatic
R2 = 0.6003
p value = 0.0006796
BALLIF_DEVELOPMENTAL_DISABILITY_P16_P12_DELETION
RAD51 Expression
−1.5
−1.0
−0.5
0.0
0.5
1.0
1.5
6
7
8
9
10
11
12

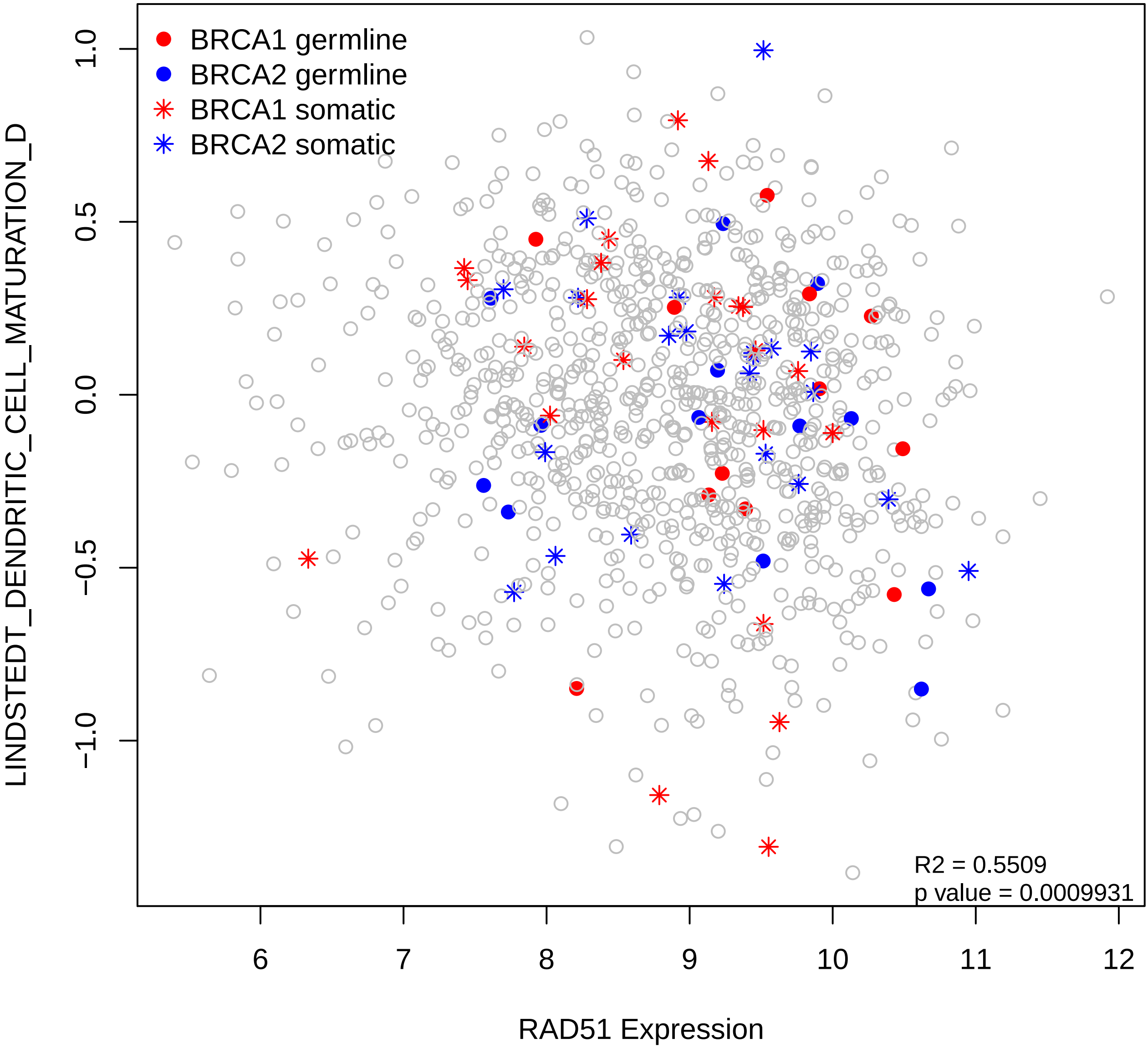

BRCA1 germline
BRCA2 germline
BRCA1 somatic
BRCA2 somatic
R2 = 0.5509
p value = 0.0009931
LINDSTEDT_DENDRITIC_CELL_MATURATION_D
RAD51 Expression
1.0
0.5
0.0
−0.5
−1.0
6
7
8
9
10
11
12

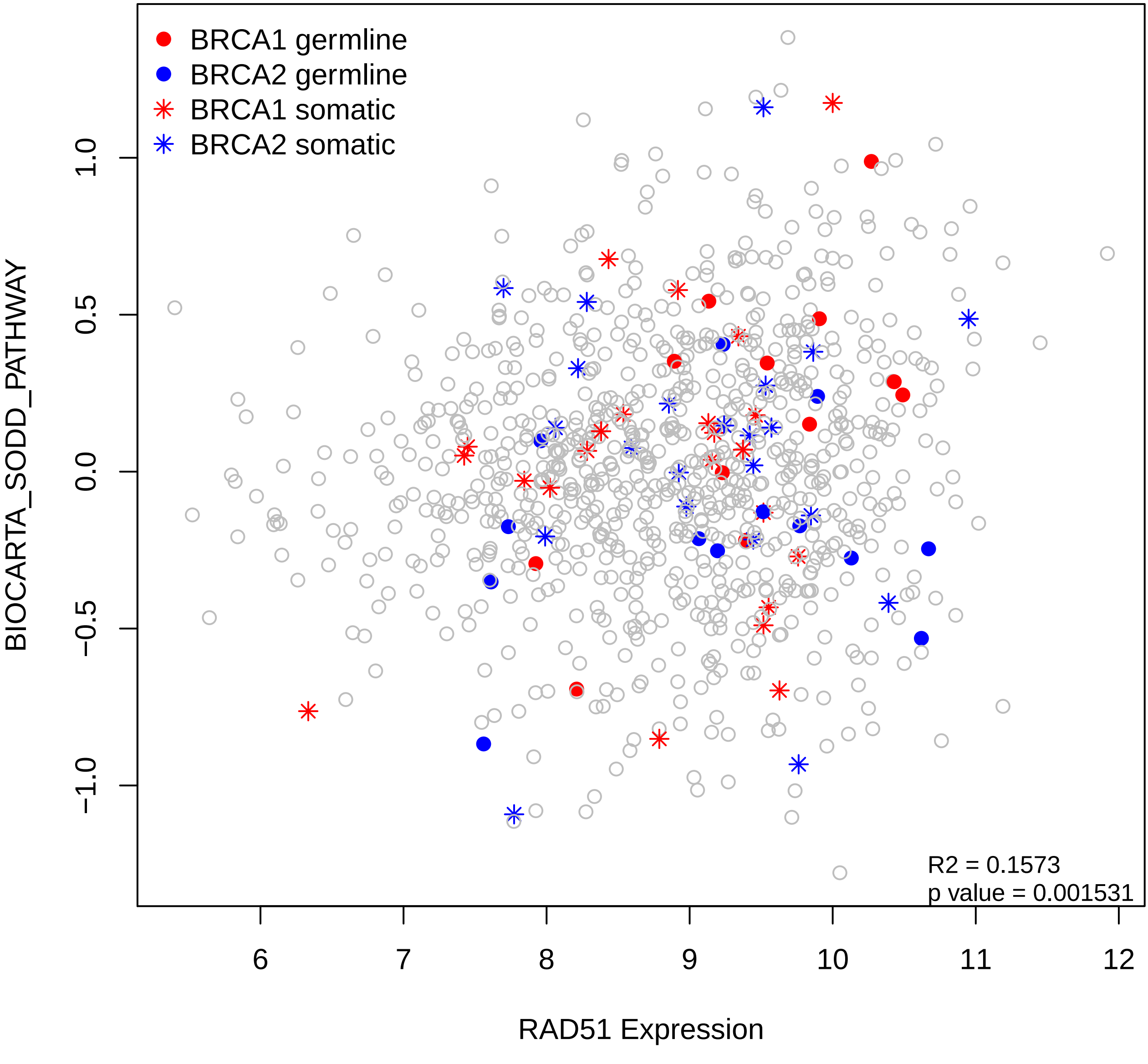
BRCA1 germline
BRCA2 germline
BRCA1 somatic
BRCA2 somatic
R2 = 0.1573
p value = 0.001531
BIOCARTA_SODD_PATHWAY
1.0
0.5
0.0
−0.5
−1.0
6
7
8
9
10
11
12
RAD51 Expression

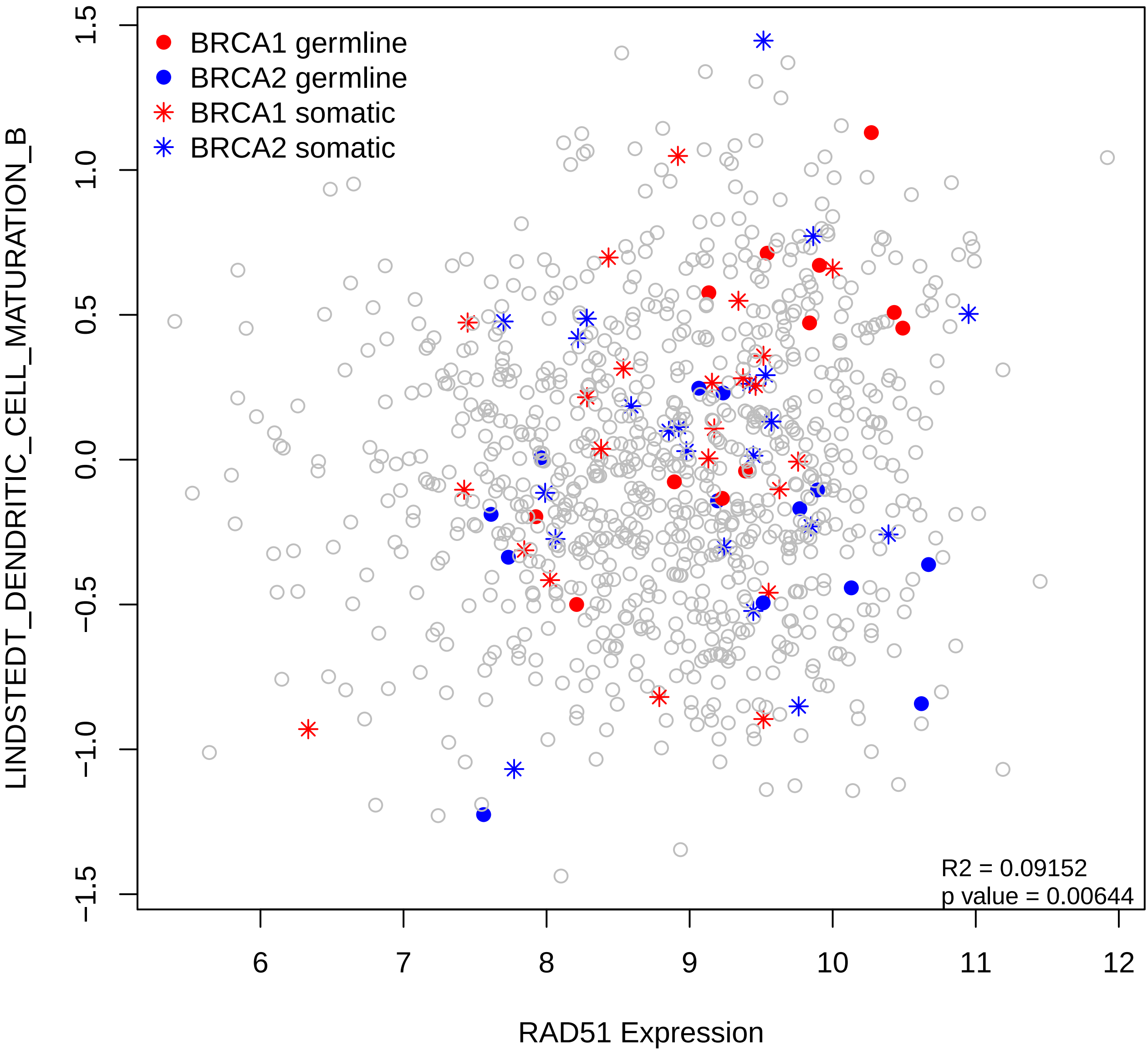
BRCA1 germline
BRCA2 germline
BRCA1 somatic
BRCA2 somatic
R2 = 0.09152
p value = 0.00644
LINDSTEDT_DENDRITIC_CELL_MATURATION_B
RAD51 Expression
1.5
1.0
0.5
0.0
−0.5
−1.0
−1.5
6
7
8
9
10
11
12

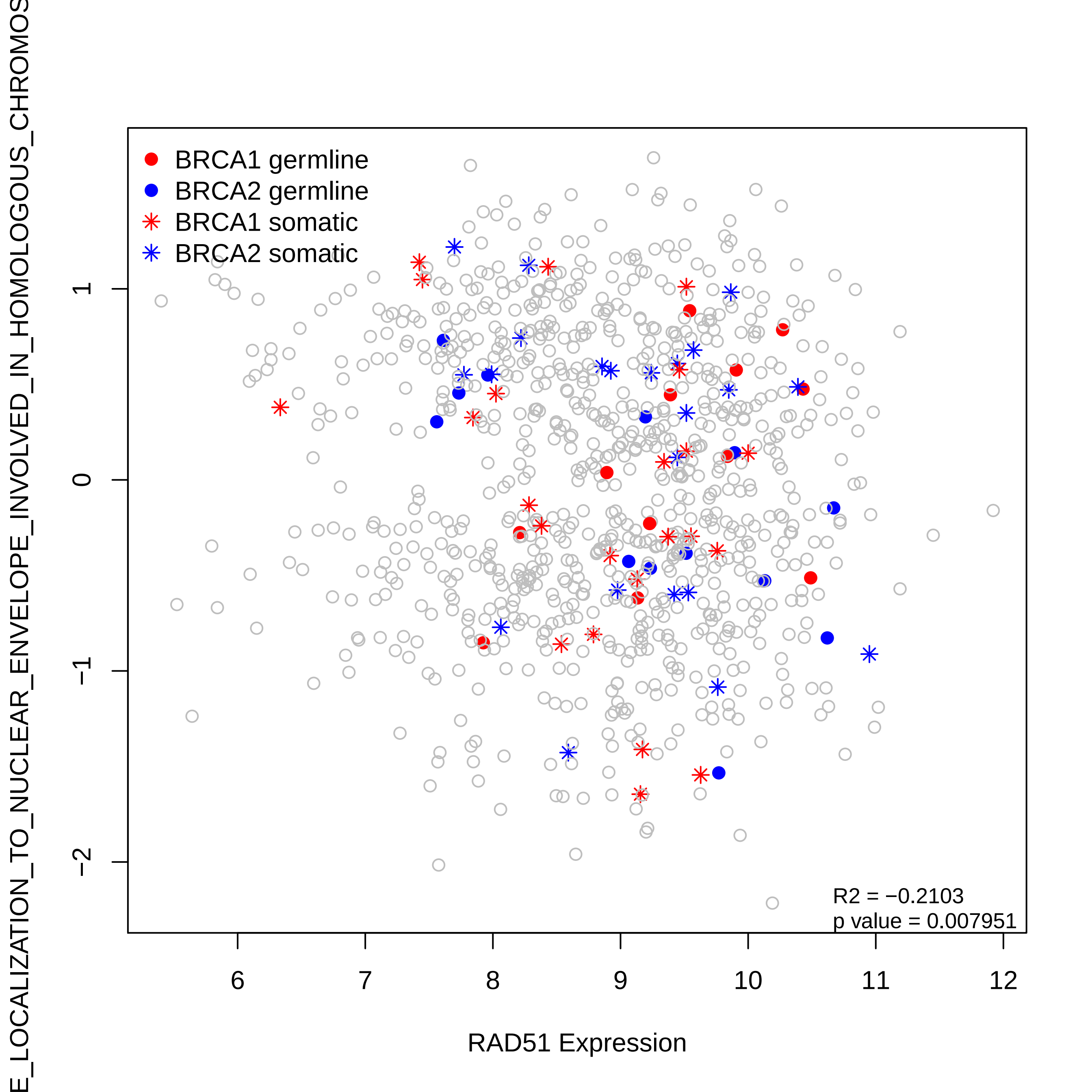

BRCA1 germline
BRCA2 germline
BRCA1 somatic
BRCA2 somatic
R2 = −0.2103
p value = 0.007951
6
7
8
9
10
11
12
RAD51 Expression
−2
−1
0
1
E_LOCALIZATION_TO_NUCLEAR_ENVELOPE_INVOLVED_IN_HOMOLOGOUS_CHROMOS

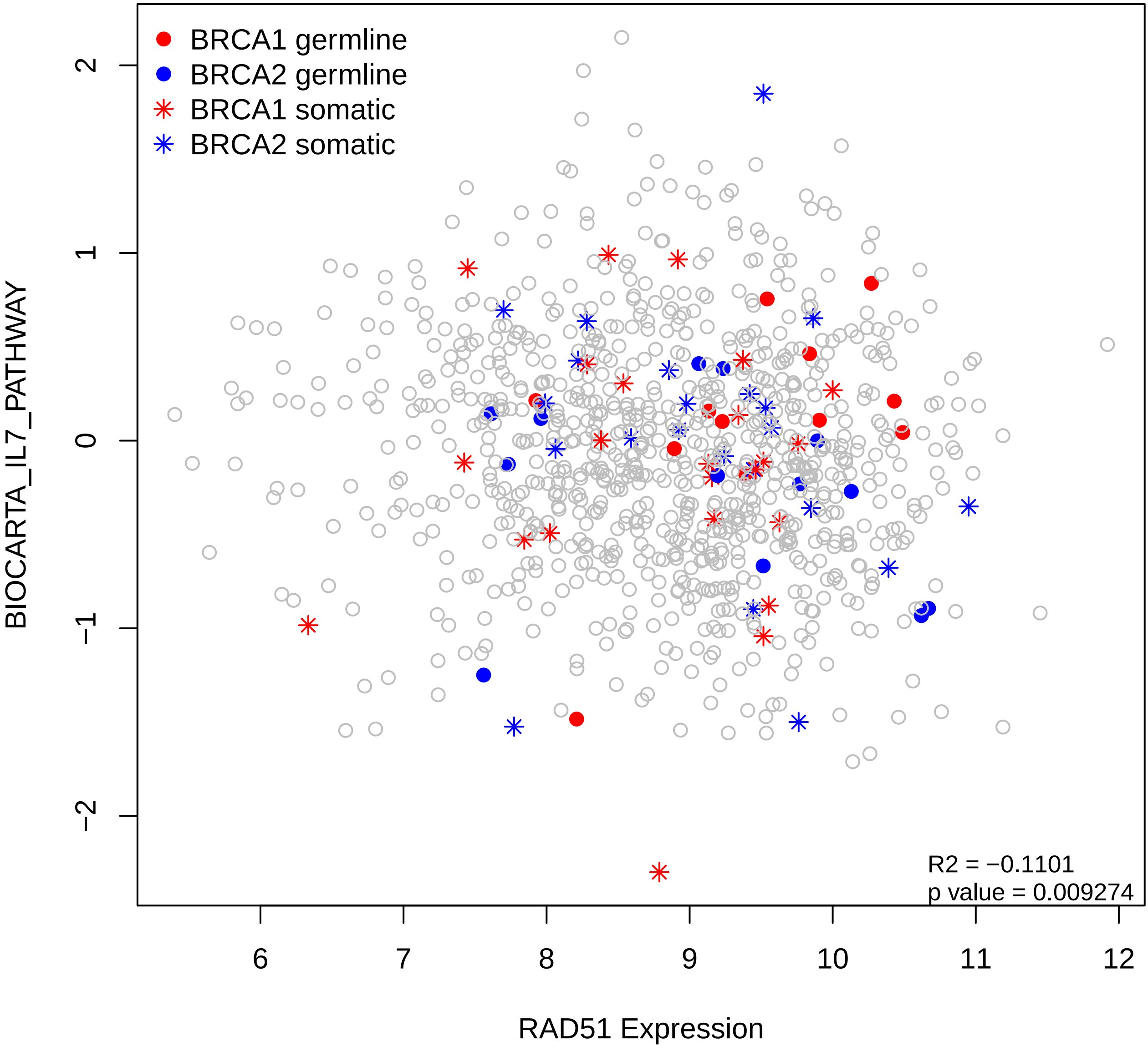

BRCA1 germline
BRCA2 germline
BRCA1 somatic
BRCA2 somatic
BIOCARTA_IL7_PATHWAY
RAD51 Expression
R2 = −0.1101
p value = 0.009274

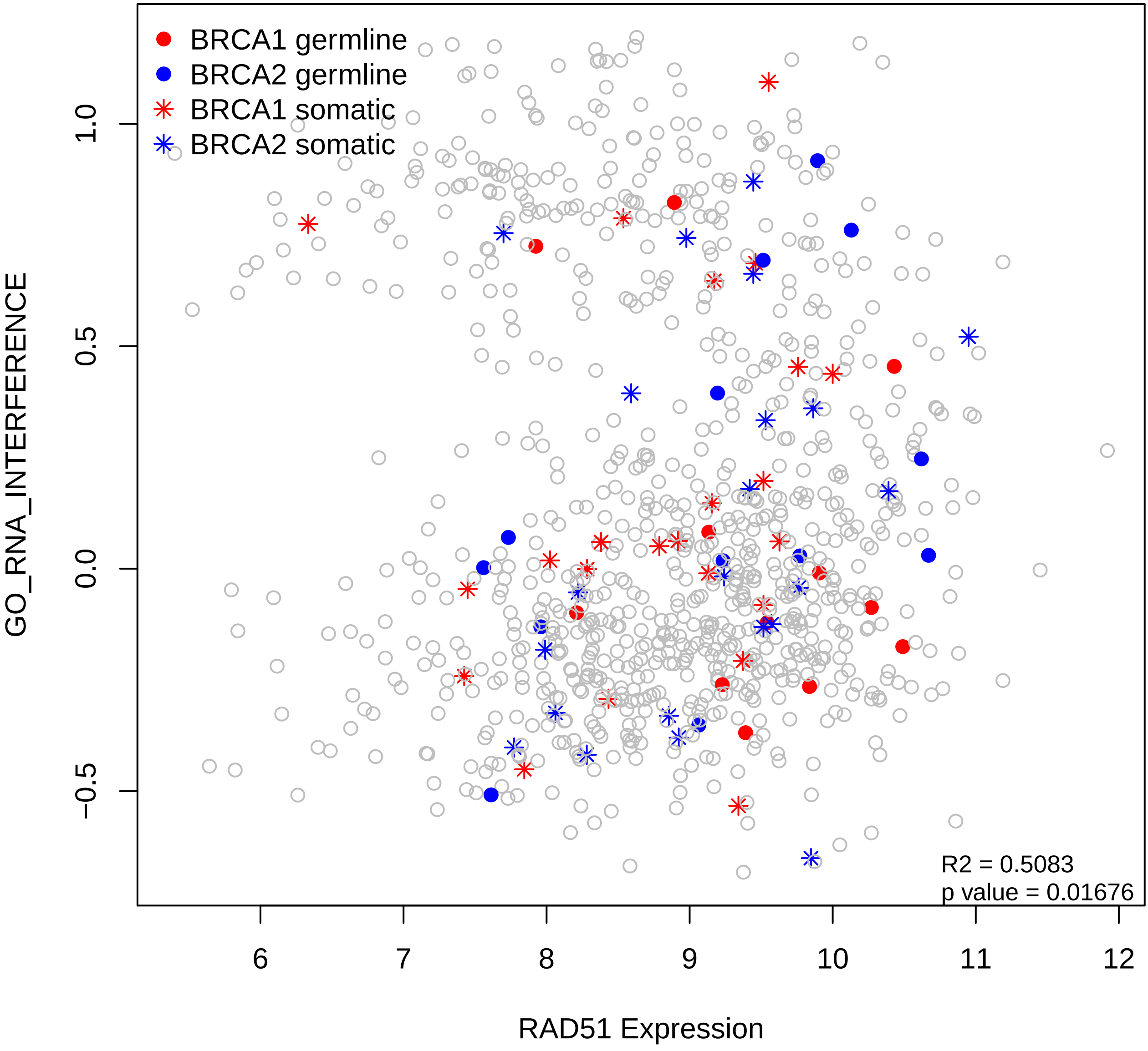

BRCA1 germline
BRCA2 germline
BRCA1 somatic
BRCA2 somatic
R2 = 0.5083
p value = 0.01676
GO_RNA_INTERFERENCE
RAD51 Expression
−0.5
0.0
0.5
1.0
6
7
8
9
10
11
12

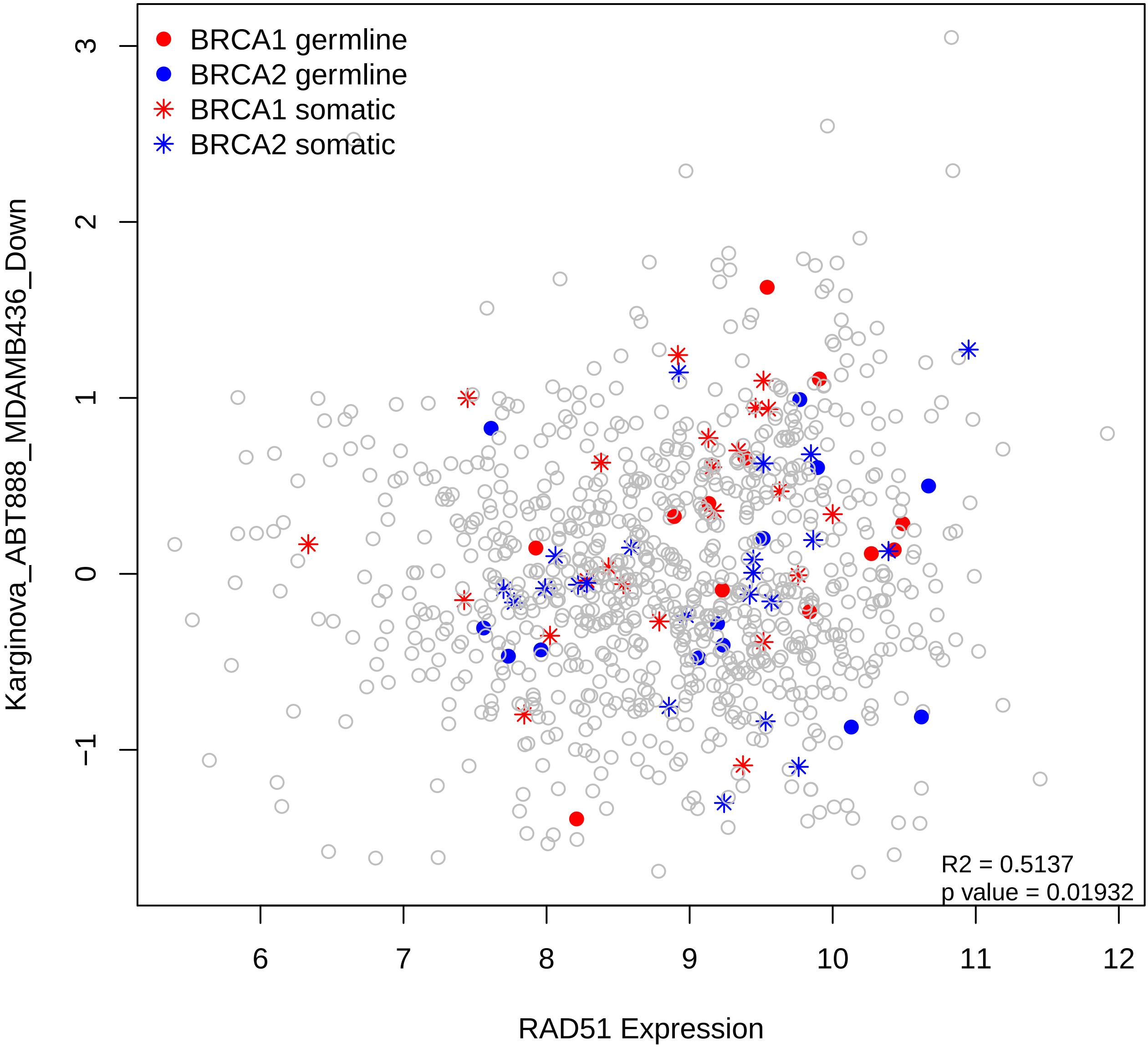

BRCA1 germline
BRCA2 germline
BRCA1 somatic
BRCA2 somatic
R2 = 0.5137
p value = 0.01932
Karginova_ABT888_MDAMB436_Down
RAD51 Expression
−1
0
1
2
3
6
7
8
9
10
11
12

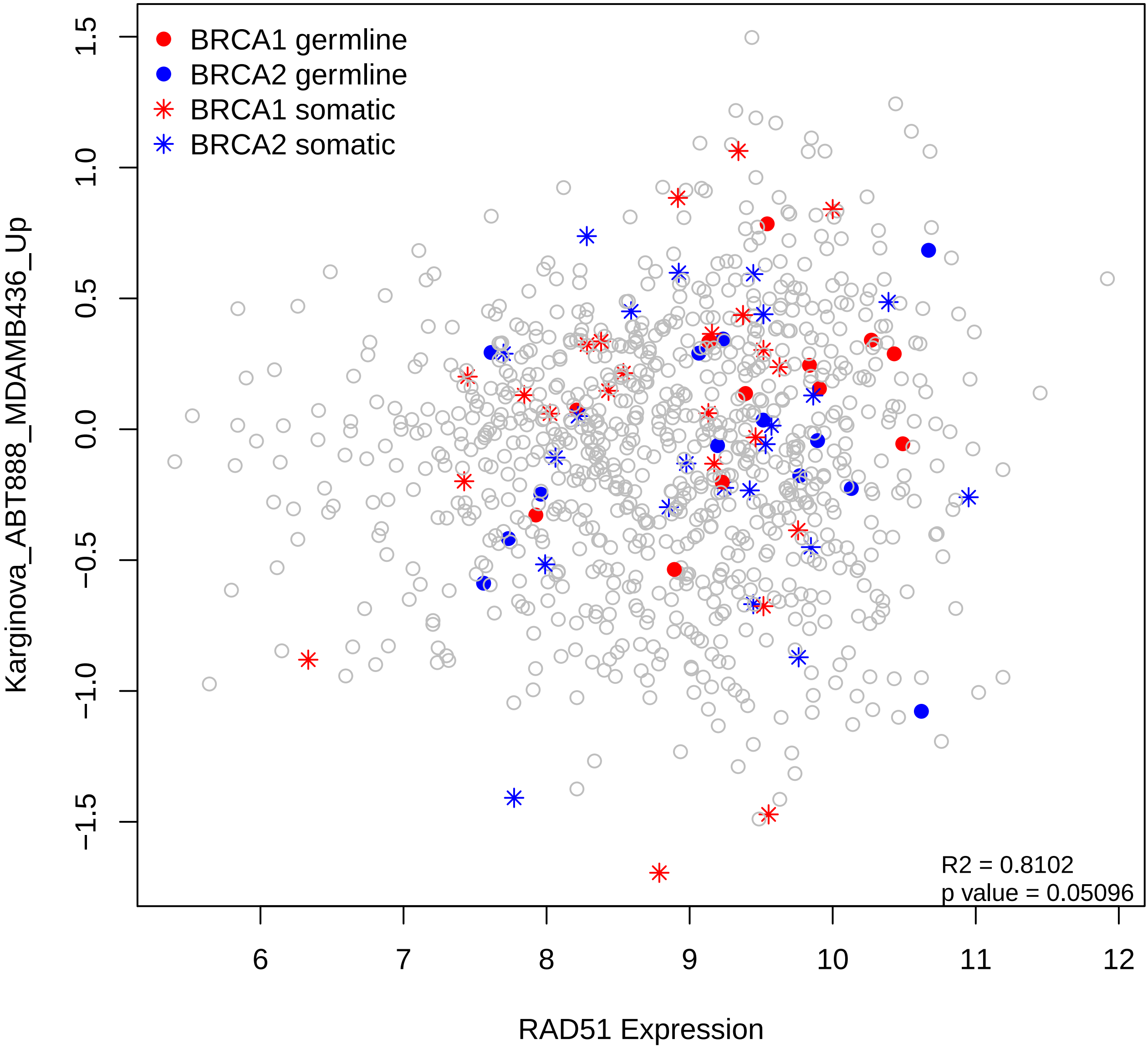

BRCA1 germline
BRCA2 germline
BRCA1 somatic
BRCA2 somatic
R2 = 0.8102
p value = 0.05096
Karginova_ABT888_MDAMB436_Up
RAD51 Expression
1.5
1.0
0.5
0.0
−0.5
−1.0
−1.5
6
7
8
9
10
11
12

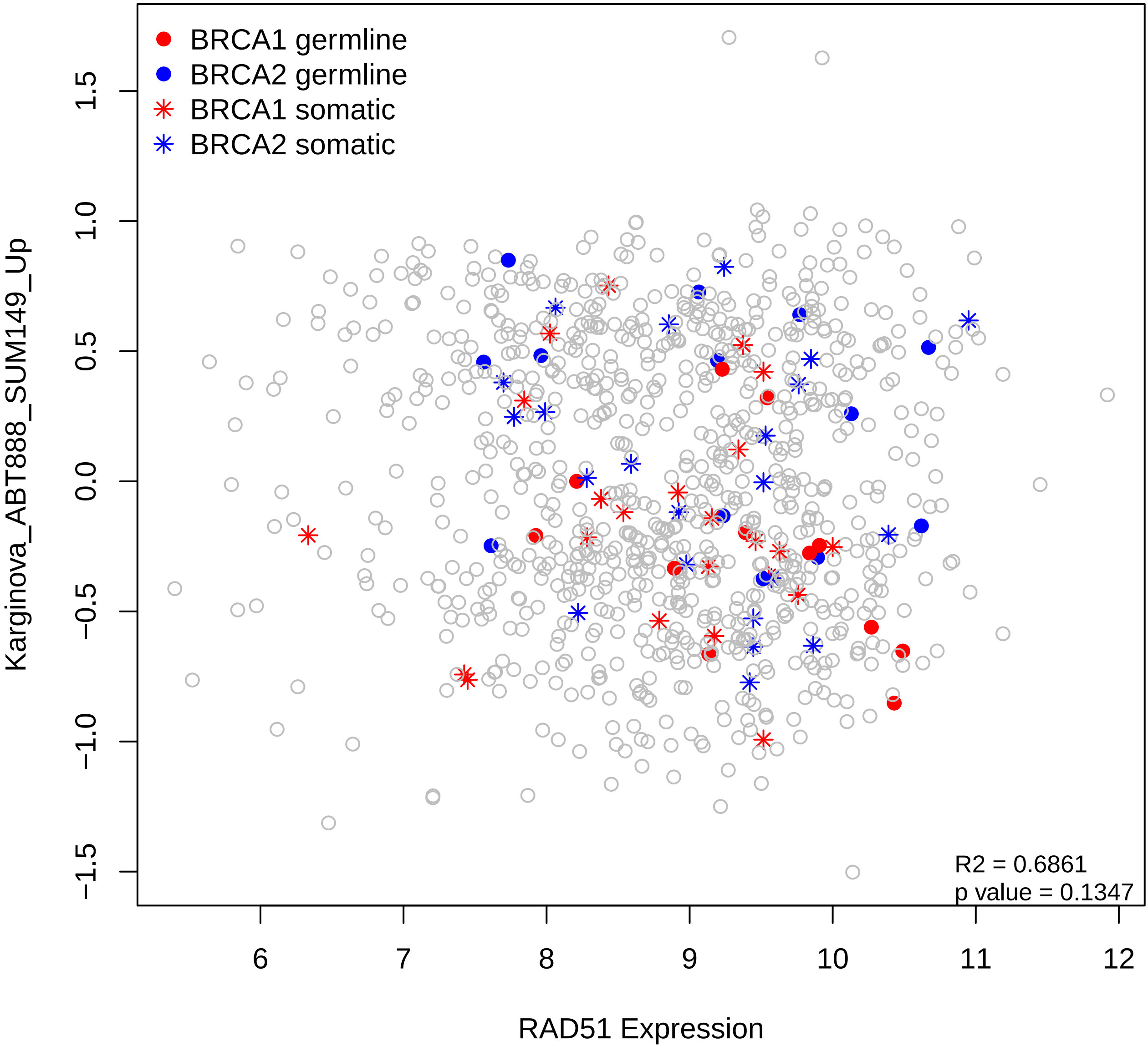

BRCA1 germline
BRCA2 germline
BRCA1 somatic
BRCA2 somatic
R2 = 0.6861
p value = 0.1347
Karginova_ABT888_SUM149_Up
RAD51 Expression
1.5
1.0
0.5
0.0
−0.5
−1.0
−1.5
6
7
8
9
10
11
12

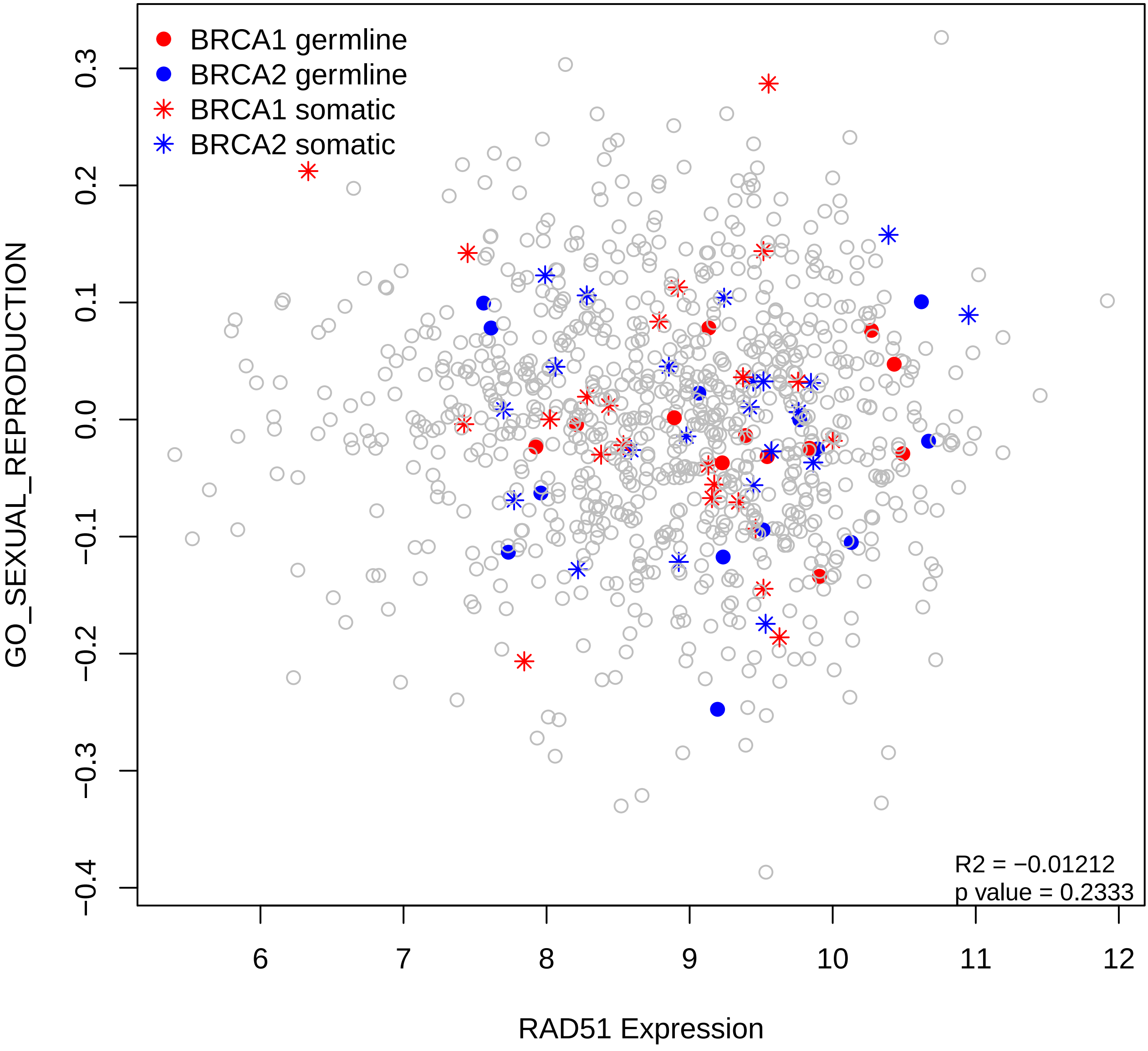

BRCA1 germline
BRCA2 germline
BRCA1 somatic
BRCA2 somatic
R2 = −0.01212
p value = 0.2333
GO_SEXUAL_REPRODUCTION
RAD51 Expression
0.3
0.2
0.1
0.0
−0.1
−0.2
−0.3
−0.4
6
7
8
9
10
11
12

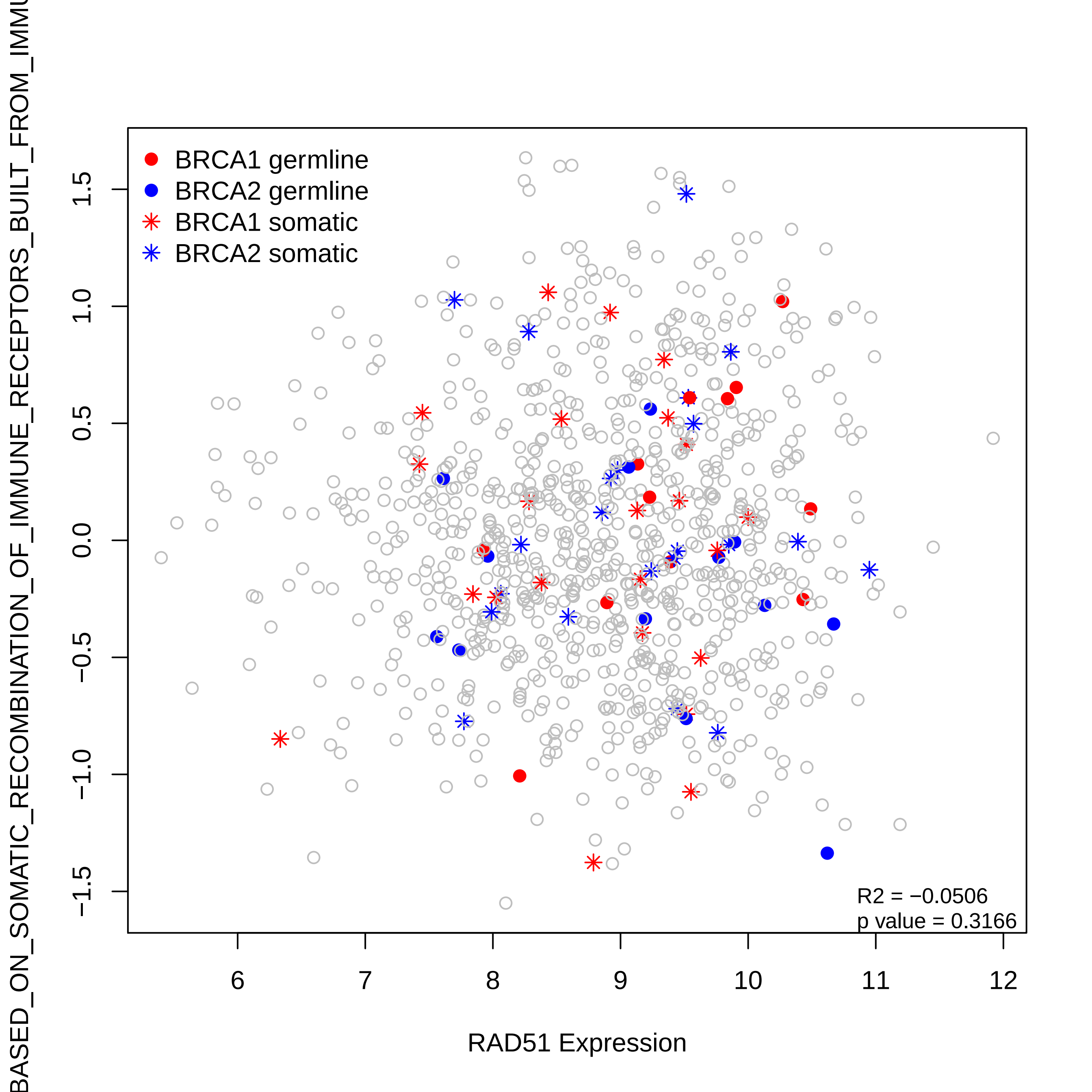
BRCA1 germline
BRCA2 germline
BRCA1 somatic
BRCA2 somatic
R2 = −0.0506
p value = 0.3166
RAD51 Expression
BASED_ON_SOMATIC_RECOMBINATION_OF_IMMUNE_RECEPTORS_BUILT_FROM_IMMU
6
7
8
9
10
11
12
−1.5
−1.0
−0.5
0.0
0.5
1.0
1.5

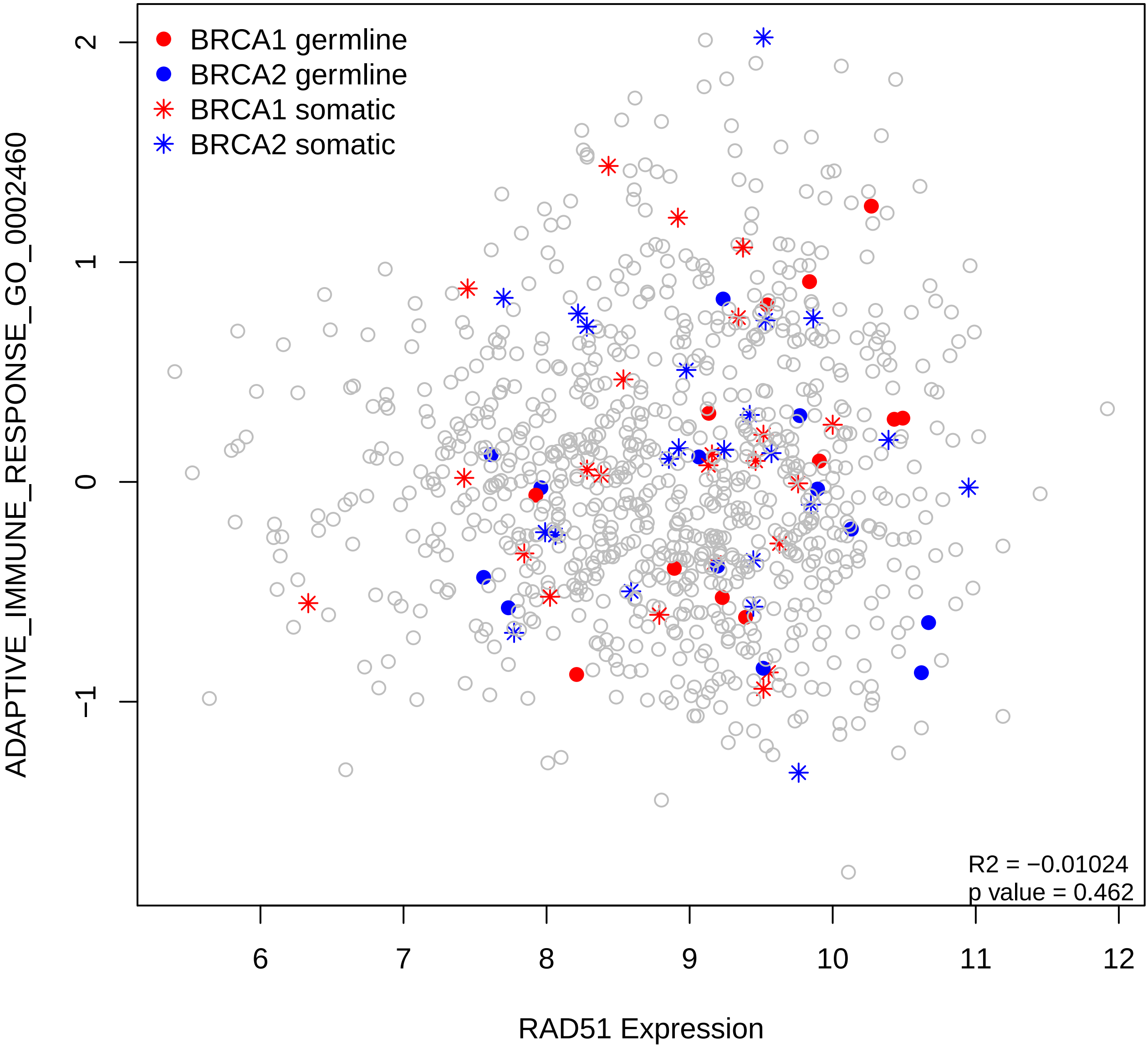
BRCA1 germline
BRCA2 germline
BRCA1 somatic
BRCA2 somatic
ADAPTIVE_IMMUNE_RESPONSE_GO_0002460
2
1
0
−1
6
7
8
9
10
11
12
RAD51 Expression
R2 = −0.01024
p value = 0.462

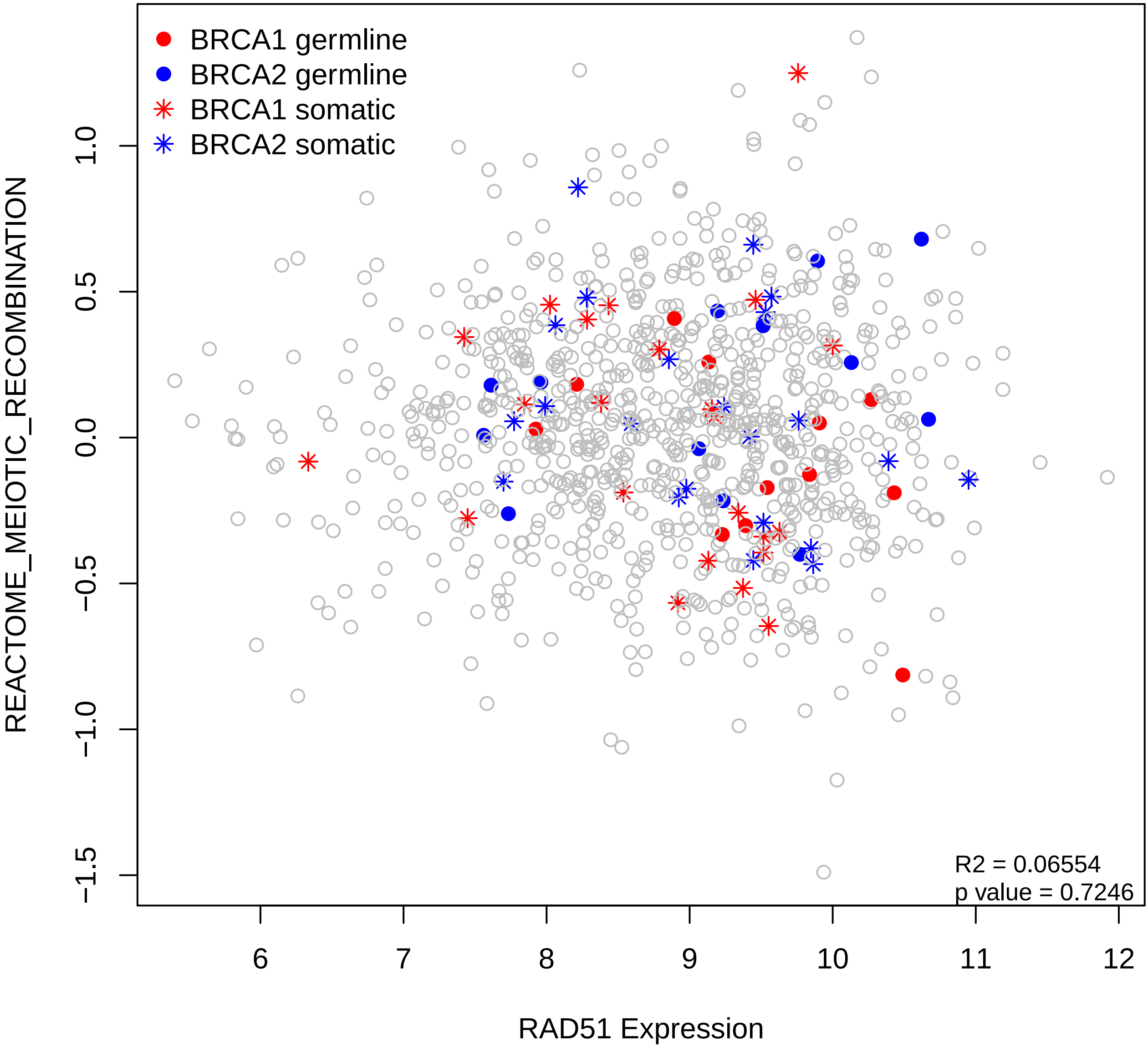
BRCA1 germline
BRCA2 germline
BRCA1 somatic
BRCA2 somatic
R2 = 0.06554
p value = 0.7246
REACTOME_MEIOTIC_RECOMBINATION
RAD51 Expression
1.0
0.5
0.0
−0.5
−1.0
−1.5
6
7
8
9
10
11
12

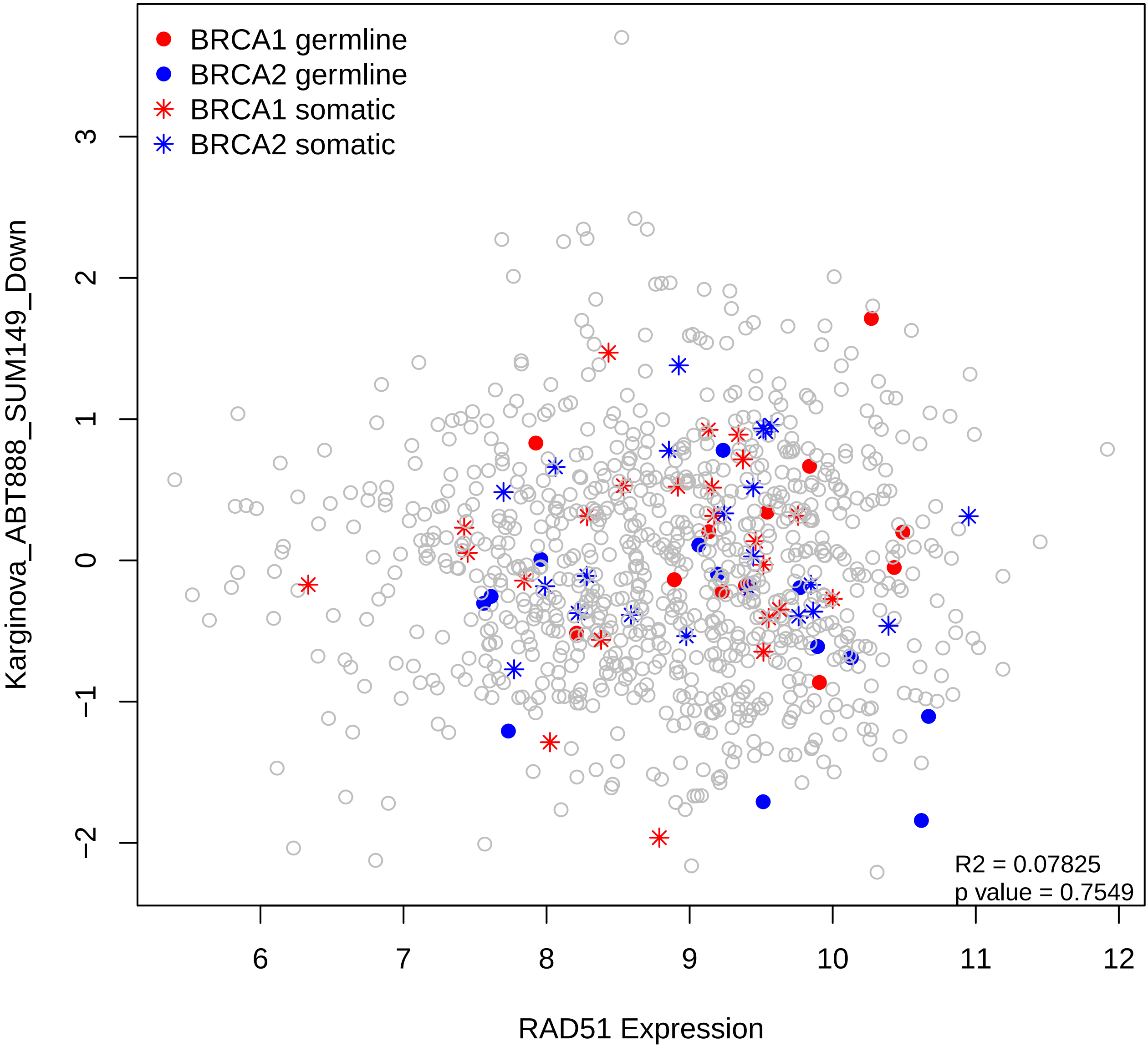
BRCA1 germline
BRCA2 germline
BRCA1 somatic
BRCA2 somatic
R2 = 0.07825
p value = 0.7549
Karginova_ABT888_SUM149_Down
RAD51 Expression
−2
−1
0
1
2
3
6
7
8
9
10
11
12
